# Engineering Smart Hydrogels Through Dynamic Polymer Networks for Controlled Drug Delivery

**DOI:** 10.3390/gels12100884

**Published:** 2026-09-30

**Authors:** Chanju Choi, Dongseong Seo, Taeho Kim, Jonghyun Park, Dongmin Yu, Sohyeon Yu, Jeongmin Shin, Simseok A. Yuk, Daekyung Sung, Hyungjun Kim

**Affiliations:** 1 Department of Chemistry and Bioscience, Kumoh National Institute of Technology, 61 Daehak-ro, Gumi 39177, Republic of Korea; ccj2001@kumoh.ac.kr (C.C.); antares913@kumoh.ac.kr (T.K.); sjqhek123@kumoh.ac.kr (J.P.); mustdoing2816@kumoh.ac.kr (D.Y.); 2 Center for Bio-Healthcare Materials, Bio-Convergence Materials R&D Division, Korea Institute of Ceramic Engineering and Technology, 202 Osongsaengmyeong 1-ro, Osong-eup, Heungdeok-gu, Cheongju 28160, Republic of Korea; ds950826@gmail.com (D.S.); aelnlouis@yonsei.ac.kr (S.Y.); minnn3926@gmail.com (J.S.); 3 Department of Applied Bioengineering, Graduate School of Convergence Science and Technology, Seoul National University, Seoul 08826, Republic of Korea; 4 Department of Chemical and Biomolecular Engineering, Yonsei University, 50 Yonsei-ro, Seodaemun-gu, Seoul 03722, Republic of Korea; 5 Department of Biomedical Engineering, Northwestern University, Evanston, IL 60208, USA; andrewsim89@gmail.com

**Keywords:** dynamic hydrogels, dynamic polymer networks, controlled drug delivery, stimuli-responsive release, molecular transport, therapeutic cargo delivery

## Abstract

Smart hydrogels are increasingly explored as adaptive platforms for controlled drug delivery because their polymer networks can respond dynamically to chemical, biological, and physical cues. Among these systems, dynamic polymer networks formed through reversible covalent and noncovalent interactions provide a unique means of coupling molecular-scale bond exchange with time-dependent changes in hydrogel structure, mechanics, degradation, and therapeutic transport. This review presents an integrated framework linking dynamic crosslink chemistry, exchange kinetics, network properties, and drug-release behavior. Dynamic covalent, supramolecular, metal–ligand, and hybrid network strategies are compared with particular emphasis on how crosslink lifetime, mesh accessibility, swelling, viscoelasticity, stress relaxation, and degradation govern therapeutic cargo loading, retention, diffusion, and release. Representative release mechanisms and kinetic models are discussed together with cargo-specific design considerations for small molecules, proteins and peptides, nucleic acids, and nanoparticle-based therapeutics. Biomedical applications are further considered according to therapeutic requirements and administration routes, including injectable local depots, topical and transdermal delivery, and regenerative systems. Finally, key translational challenges involving physiological complexity, biocompatibility, reproducibility, scale-up, sterilization, and storage stability are critically assessed. By connecting molecular interaction dynamics with network-level behavior and therapeutic performance, this review provides design principles for developing more predictable, programmable, and clinically translatable dynamic hydrogel drug-delivery systems.

## 1. Introduction

Hydrogels are three-dimensional, water-swollen polymer networks that combine high water content with tunable physicochemical and mechanical properties. Since their early development as biomaterials, hydrogels have evolved from relatively simple crosslinked polymer matrices into sophisticated functional materials capable of interacting with biological environments [1,2,3,4]. Their structural similarity to hydrated soft tissues, adjustable porosity, biocompatibility, and ability to accommodate diverse therapeutic molecules have made them particularly attractive for biomedical applications. Among these applications, controlled drug delivery has received considerable attention because hydrogels can serve as localized depots that retain therapeutic agents at the site of administration and regulate their release over prolonged periods [2,3]. Compared with conventional systemic administration, localized hydrogel-based delivery can potentially reduce off-target exposure, maintain therapeutically relevant local concentrations, and improve the stability and residence time of encapsulated drugs. Furthermore, the chemical versatility of natural and synthetic polymers enables the incorporation of small-molecule drugs, proteins, peptides, nucleic acids, and nanocarrier-based therapeutics within hydrogel matrices [3,5,6].

The release of therapeutic agents from hydrogels is fundamentally governed by interactions among the polymer network, water, therapeutic cargo, and surrounding biological environment. Diffusion through the aqueous pores of the network is one of the most common release mechanisms, but swelling, polymer degradation, network erosion, and reversible drug–polymer interactions can also substantially influence release kinetics [2,3,5]. Network parameters such as polymer concentration, crosslinking density, mesh size, and equilibrium swelling determine the accessibility and mobility of encapsulated molecules and therefore provide important design variables for controlled release [5,7]. For macromolecular therapeutics in particular, the relationship between cargo size and hydrogel mesh size becomes critical, because molecular diffusion may decrease substantially when the hydrodynamic dimensions of the cargo approach the characteristic dimensions of the polymer network [6,7]. These principles have enabled conventional hydrogels to achieve sustained drug delivery; however, in permanently crosslinked networks, most structural and transport characteristics are largely fixed during material fabrication. As a result, the release profile is often determined by static parameters established before administration rather than by adaptive changes occurring during therapeutic use.

The development of stimuli-responsive or “smart” hydrogels has expanded this conventional paradigm by introducing polymer networks capable of changing their physicochemical properties in response to environmental signals [8,9,10,11]. Temperature, pH, ionic strength, redox conditions, enzymes, light, and other endogenous or externally applied stimuli can alter polymer conformation, swelling, intermolecular association, degradation, or network permeability, thereby providing mechanisms for triggered or on-demand therapeutic release [8,9]. Such responsiveness is particularly attractive for disease-associated microenvironments, where abnormal pH, oxidative stress, enzymatic activity, or local biochemical signals can potentially be exploited to initiate therapeutic delivery. Recent developments have therefore shifted hydrogel design from passive sustained-release matrices toward materials that can actively interact with their surroundings and modify their behavior over time [10,11]. Nevertheless, describing smart hydrogels solely according to the type of external stimulus does not fully capture an important molecular feature underlying many of these systems. The response of a hydrogel ultimately depends on how its polymer chains and crosslinks reorganize at the molecular and network levels.

Conventional chemically crosslinked hydrogels are typically stabilized by permanent covalent bonds that provide structural integrity but permit limited rearrangement of the network after gel formation. In contrast, dynamic polymer networks incorporate reversible crosslinks that can dissociate, exchange, and reform over experimentally or biologically relevant timescales [12,13,14,15,16]. These crosslinks can arise from dynamic covalent reactions, including imine, hydrazone, boronic ester, disulfide, and other exchangeable covalent bonds, or from reversible noncovalent interactions such as hydrogen bonding, host–guest recognition, electrostatic interactions, hydrophobic association, and metal–ligand coordination. Dynamic covalent networks combine aspects of the mechanical stability associated with covalent crosslinking with the adaptability produced by reversible bond exchange, whereas supramolecular networks rely on transient molecular associations to generate highly reversible structures [12,13,14,15,16]. Because the lifetime, association strength, and exchange kinetics of these interactions can be chemically engineered, dynamic networks provide molecular-level control over time-dependent hydrogel behavior that is fundamentally different from that of conventional static networks.

The reversibility of dynamic crosslinks enables stress relaxation, shear thinning, self-healing, and structural recovery [17,18,19,20,21]. More importantly for drug delivery, crosslink lifetime, exchange kinetics, network architecture, and environmental conditions can alter mesh accessibility, relaxation, erosion, and cargo transport [22,23,24,25,26,27]. Dynamic hydrogels should therefore be considered temporally evolving transport environments rather than static drug reservoirs. Previous reviews have extensively addressed hydrogel-based controlled drug delivery, stimuli-responsive systems, dynamic covalent hydrogels, and supramolecular hydrogel platforms. However, these studies have generally focused on specific material classes, stimuli, or application areas. In contrast, the present review integrates dynamic crosslink chemistry, exchange kinetics, time-dependent network properties, molecular transport, and therapeutic release within a unified framework, emphasizing how molecular-scale dynamics govern drug-delivery performance across multiple length and time scales.

From this perspective, rational engineering of dynamic polymer networks provides an opportunity to control therapeutic delivery across multiple length and time scales. At the molecular scale, bond association strength, dissociation kinetics, crosslinker valency, and polymer–cargo interactions determine the mobility and retention of therapeutic molecules. At the network scale, these molecular interactions collectively regulate crosslink density, mesh size, swelling, viscoelasticity, stress relaxation, degradation, and network rearrangement. These properties subsequently govern macroscopic release behaviors ranging from rapid diffusion and burst release to sustained, stimuli-triggered, pulsatile, or sequential delivery. Establishing quantitative and mechanistic relationships between these scales is therefore essential for transforming dynamic hydrogels from empirically optimized formulations into predictable and programmable drug delivery systems.

Accordingly, this review discusses recent progress in engineering smart hydrogels through dynamic polymer networks for controlled drug delivery. The molecular principles underlying dynamic covalent, supramolecular, metal–ligand, and hybrid crosslinking strategies are first described, followed by an examination of how these interactions determine hydrogel network architecture and time-dependent physicochemical properties. Particular emphasis is placed on the relationships among crosslinking dynamics, mesh size, swelling, viscoelasticity, stress relaxation, degradation, and therapeutic cargo transport. The mechanisms by which dynamic networks regulate drug loading, diffusion, retention, and controlled release are subsequently discussed, together with strategies for sustained and stimuli-responsive delivery. Representative systems for the delivery of small-molecule drugs, proteins, nucleic acids, and nanoparticle-based therapeutics are highlighted. Finally, the remaining challenges associated with release predictability, mechanical stability, biocompatibility, manufacturing, and clinical translation are considered, along with emerging opportunities for the development of adaptive and programmable hydrogel-based therapeutic delivery platforms. Relevant literature was identified through Web of Science, PubMed, and Google Scholar using keywords such as “dynamic hydrogel,” “dynamic polymer network,” “controlled drug delivery,” “dynamic covalent hydrogel,” “supramolecular hydrogel,” and “stimuli-responsive hydrogel.” Studies published primarily within the past decade were considered, together with earlier seminal reports providing key mechanistic or conceptual foundations. Original research articles directly addressing dynamic crosslinking, network properties, therapeutic transport, and drug release were prioritized, while redundant or less directly relevant studies were not emphasized. The overall design logic and scope of this review are summarized in Figure 1, which organizes dynamic hydrogel systems according to molecular design, network properties, delivery behavior, and therapeutic implementation.

## 2. Molecular Design of Dynamic Polymer Networks

Dynamic polymer networks rely on reversible covalent, supramolecular, metal–ligand, or hybrid interactions that permit time-dependent network reorganization [12,16]. Their drug-delivery behavior depends on interaction strength, exchange kinetics, polymer architecture, environmental sensitivity, and cargo compatibility. Rapid exchange generally favors injectability and recovery, whereas slower dynamics support structural persistence and cargo retention [23,24]. The following subsections compare these interaction classes and their implications for therapeutic transport.

### 2.1. Dynamic Covalent Networks

Dynamic covalent networks are constructed through covalent bonds that can reversibly dissociate, reform, or undergo exchange under appropriate conditions. Unlike permanently crosslinked hydrogels, these networks can reorganize over time while maintaining overall structural integrity, thereby combining covalent stability with molecular adaptability [12]. Such dynamic behavior is particularly advantageous for controlled drug delivery because the kinetics and equilibrium of reversible bonds can simultaneously influence gelation, stress relaxation, self-healing, degradation, and molecular transport. Importantly, macroscopic hydrogel properties are determined not only by bond strength but also by crosslinker structure, valency, reaction kinetics, and environmental conditions. Recent studies on hydrazone-based networks have demonstrated that variations in crosslinker length and functionality can markedly alter network formation and mechanical behavior, highlighting the importance of molecular-level reaction parameters in hydrogel design [24]. Among the dynamic covalent reactions employed in smart hydrogels, Schiff base/imine, hydrazone, boronate ester, disulfide, and reversible Diels–Alder chemistries have received particular attention.

Schiff base reactions between primary amines and aldehydes or ketones are widely used to construct injectable and self-healing hydrogels because imine bonds can form rapidly under mild aqueous conditions without radical initiators or harsh reaction conditions. Polysaccharides are particularly suitable for this strategy because aldehyde functionalities can be introduced into alginate, hyaluronic acid, dextran, and related polymers, whereas chitosan and its derivatives provide abundant primary amines. Wei et al. developed N-carboxyethyl chitosan/oxidized sodium alginate hydrogels in which reversible imine bonds enabled self-healing and recovery following injection [28]. Similarly, Lü et al. prepared injectable carbohydrate-based hydrogels using aldehyde-functionalized chondroitin sulfate and N-succinyl chitosan, demonstrating that dynamic Schiff base formation could provide rapid gelation together with self-healing behavior [29]. Ding et al. also reported a self-crosslinked polysaccharide hydrogel whose autonomous healing behavior was associated with reversible imine formation [30]. These studies demonstrate that functional-group density, polymer concentration, and stoichiometric balance between aldehyde and amine groups provide practical means of regulating network formation and mechanical recovery. For drug-delivery applications, imine bonds additionally offer pH-dependent hydrolysis, which can facilitate accelerated network relaxation or degradation in acidic environments. However, excessive imine lability may cause rapid swelling, network erosion, and premature cargo release; therefore, the stability of imine networks must be carefully balanced against the desired responsiveness.

Hydrazone bonds represent a related dynamic covalent chemistry but generally provide greater hydrolytic stability than simple imines. They are formed through reactions between aldehydes or ketones and hydrazine- or hydrazide-containing groups, allowing reversible network formation while maintaining relatively prolonged structural integrity. Patenaude et al. showed that the structure and density of carbonyl groups strongly affected the gelation time, morphology, degradation, and mechanical properties of ketone–hydrazide-crosslinked injectable hydrogels [31]. Hydrazone chemistry has also been directly applied to controlled drug release. Sharma et al. developed self-healing xanthan–poly(ethylene glycol) hydrogels in which dynamic hydrazone linkages provided injectability, pH-dependent degradation, and controlled doxorubicin release [19]. Faster drug release under acidic conditions demonstrated how reversible bond chemistry could couple environmental changes to therapeutic transport. Molecular characteristics of the crosslinker are also critical, as demonstrated by Morgan et al., who showed that crosslinker length, valency, and reaction kinetics collectively governed the formation and mechanical properties of hydrazone networks [24]. These findings indicate that hydrazone chemistry provides a useful balance between network stability and dynamic exchange, particularly when sustained cargo retention is required.

Boronate ester bonds, formed reversibly between boronic acids and cis-diol-containing molecules, provide another versatile dynamic covalent platform. Their association and dissociation can occur rapidly in aqueous environments, and the equilibrium is strongly affected by pH, boronic acid structure, and diol configuration. These properties enable dynamic network rearrangement together with responsiveness to physiologically relevant signals such as glucose and changes in local pH. Figueiredo et al. developed injectable self-healing hyaluronic acid hydrogels using benzoxaborin–saccharide interactions, allowing dynamic boronate ester formation near physiological pH [32]. Hong et al. similarly demonstrated that reversible interactions between boronic acid and alginate generated hydrogels with self-healing, stretchable, remoldable, and stimuli-responsive properties [33]. These studies highlight the ability of boronate ester chemistry to translate molecular association–dissociation processes into macroscopic dynamic behavior.

The relevance of boronate ester dynamics to drug transport has been demonstrated more directly in controlled-release systems. Marco-Dufort et al. showed that biomolecule release from boronic ester-based dynamic covalent hydrogels depended strongly on network properties and environmental conditions that shifted the equilibrium of reversible crosslinks [23]. In their system, surface erosion played a major role in biomolecule release, demonstrating that transport from dynamic hydrogels cannot always be interpreted solely as diffusion through a fixed mesh. More recently, Oyarzún et al. developed boronic-acid-functionalized alginate/PVA hydrogels for metoclopramide delivery and showed that changes in pH and applied shear altered hydrogel rheology and drug release [34]. These findings illustrate an important feature of dynamic networks: the transport environment experienced by an encapsulated therapeutic can evolve in response to external chemical or mechanical conditions. Nevertheless, boronate ester systems require careful optimization because conventional phenylboronic acid derivatives may show limited complexation at physiological pH, while endogenous sugars and other competing diols can influence network stability.

Disulfide chemistry provides dynamic and redox-responsive behavior through thiol–disulfide exchange or reductive cleavage. These two mechanisms are related but should be distinguished when evaluating dynamic hydrogel networks. Thiol–disulfide exchange can enable reversible rearrangement of covalent junctions and thereby facilitate self-healing. Yu et al. developed injectable hydrogels using thiol-functionalized F127 and dithiolane-modified PEG, in which thiol–disulfide exchange allowed network reconstruction after mechanical disruption [35]. In contrast, many drug-delivery systems primarily exploit disulfide cleavage under reducing conditions. Kılıç Boz et al. developed redox-responsive PEG-based hydrogels capable of sustained biomacromolecule release followed by accelerated, on-demand release in the presence of reducing agents [36]. Because intracellular and pathological environments may contain elevated concentrations of glutathione or other reducing species, disulfide-containing networks provide an effective means of coupling the redox microenvironment to network degradation and cargo release. However, systems relying mainly on irreversible reduction should more accurately be regarded as redox-degradable networks unless thiol–disulfide exchange actively contributes to network reorganization.

Diels–Alder chemistry provides a complementary dynamic covalent strategy with generally slower kinetics and greater structural persistence. The cycloaddition between a diene and dienophile can proceed without radical initiators or metal catalysts, while suitable Diels–Alder adducts can undergo retro-Diels–Alder dissociation. Koehler et al. used reversible furan–maleimide chemistry to tether peptides within PEG hydrogels and demonstrated that the release rate could be regulated by temperature and the density of available Diels–Alder binding sites [37]. The same principle was subsequently applied to sustained dexamethasone delivery, linking reaction-controlled release to osteogenic differentiation of human mesenchymal stem cells [38]. Reversible Diels–Alder reactions have also been used directly to construct self-healing networks. Wei et al. prepared dextran-based hydrogels that recovered through reversible Diels–Alder crosslinking under physiological conditions [39], whereas Yu et al. combined Diels–Alder crosslinks with dynamic acylhydrazone bonds to obtain hydrogels with structural integrity, self-healing, and tissue adhesion [40].

The comparatively slow reversibility of some Diels–Alder systems can be advantageous when long-term drug retention is required. Ilochonwu et al. prepared hyaluronic acid/PEG hydrogels by reacting furan-functionalized hyaluronic acid with four-arm PEG–maleimide [41]. The networks underwent gradual degradation associated with retro-Diels–Alder chemistry, and one formulation sustained bevacizumab release for more than 400 days through a combination of diffusion, swelling, and hydrogel degradation [41]. However, Diels–Alder reactions should not automatically be classified as rapidly dynamic under physiological conditions. Classical furan–maleimide systems may exhibit relatively slow retro-Diels–Alder kinetics at 37 °C and can therefore behave as slowly degradable covalent networks rather than rapidly exchanging self-healing materials. Likewise, bioorthogonal reactions such as inverse-electron-demand Diels–Alder cycloadditions are highly useful for in situ hydrogel formation but are typically effectively irreversible under physiological conditions.

Overall, dynamic covalent chemistry provides a broad spectrum of bond lifetimes and environmental sensitivities for controlled drug-delivery hydrogels. Imine bonds favor rapid in situ formation and pH-dependent hydrolysis, whereas hydrazone and acylhydrazone bonds generally provide greater aqueous stability while maintaining reversible behavior. Boronate ester networks offer rapid exchange and responsiveness to pH, glucose, and competing diols, while disulfide chemistry enables redox-dependent network rearrangement or cleavage. Diels–Alder chemistry provides comparatively stable networks suitable for slower network evolution and prolonged cargo retention. Therefore, the optimal dynamic covalent bond should be selected according to its exchange kinetics and environmental sensitivity rather than bond strength alone. Matching these molecular timescales with network relaxation, therapeutic cargo properties, and the desired duration of release is essential for translating dynamic covalent chemistry into predictable and controllable drug-delivery behavior.

### 2.2. Supramolecular and Noncovalent Networks

Supramolecular hydrogels are assembled through reversible noncovalent interactions that collectively generate stable three-dimensional networks despite their relatively weak individual association energies [16]. Because these interactions continuously associate and dissociate, supramolecular networks can reorganize in response to mechanical deformation or environmental changes, giving rise to shear thinning, self-healing, stress relaxation, and injectability. From a drug-delivery perspective, noncovalent interactions are particularly attractive because they can serve both as dynamic network junctions and as binding sites for therapeutic cargos. Thus, hydrogen bonding, host–guest recognition, hydrophobic association, π–π stacking, and ionic interactions can regulate not only hydrogel mechanics but also drug partitioning, retention, diffusion, and release.

Hydrogen bonding is one of the most fundamental mechanisms for forming supramolecular hydrogel networks. Although individual hydrogen bonds are relatively weak in aqueous environments, cooperative interactions among multiple hydrogen-bonding motifs can produce stable yet reversible crosslinks. Guo et al. developed supramolecular hydrogels containing 2-ureido-4[1H]-pyrimidinone (UPy) groups, which form self-complementary dimers through quadruple hydrogen bonding [42]. Localization of the UPy units within hydrophobic domains protected these interactions from water and generated mechanically robust networks. Similarly, Lu et al. combined hydrophobic association with quadruple UPy hydrogen bonding to construct injectable hydrogels with improved mechanical strength and self-healing behavior [43]. These studies demonstrate that hydrogen bonding often acts cooperatively with other noncovalent interactions, allowing local molecular organization to strengthen otherwise weak reversible associations. Such cooperative networks are advantageous for drug delivery because they can recover following injection while retaining sufficient structural stability to restrict rapid cargo diffusion.

Host–guest interactions offer greater molecular specificity because network formation is based on complementary recognition between a host cavity and a guest molecule. Cyclodextrins are among the most extensively used host molecules in biomedical hydrogels, particularly in combination with hydrophobic guests such as adamantane. Guest–host hydrogels based on β-cyclodextrin- and adamantane-modified hyaluronic acid exhibit reversible network assembly, shear-thinning behavior, and rapid recovery following deformation [17]. Importantly, host–guest interactions can also directly control cargo retention. Mealy et al. showed that β-cyclodextrin-containing hyaluronic acid hydrogels slowed the diffusion of hydrophobic small molecules through affinity-mediated interactions, extending drug retention for up to approximately three weeks [44]. By varying cyclodextrin concentration and drug–host affinity, release of doxycycline and doxorubicin could be systematically controlled, demonstrating that molecular affinity provides an additional design parameter beyond network mesh size.

Host–guest networks have also been adapted for the localized delivery of macromolecular therapeutics. Wang et al. developed an injectable hydrogel formed between adamantane-modified PEG and β-cyclodextrin-modified polyethylenimine for local siRNA delivery [45]. The system combined reversible supramolecular network formation with the nucleic-acid-complexing ability of polyethylenimine, illustrating how network assembly and therapeutic binding can be incorporated within the same material. Other macrocyclic hosts can provide even stronger and more tunable interactions. Chen et al. demonstrated that the gelation kinetics of cucurbit[7]uril–adamantane-crosslinked hydrogels could be adjusted from seconds to hours using competing guest molecules [46]. This finding emphasizes that the association and dissociation kinetics of host–guest pairs, rather than equilibrium affinity alone, can be exploited to regulate the temporal behavior of dynamic hydrogel networks. As illustrated in Figure 2, competitive guest binding provides a simple molecular strategy for regulating the gelation kinetics of CB[7]–adamantane supramolecular hydrogels by transiently occupying the CB[7] cavities before formation of the stronger host–guest crosslinks.

Hydrophobic association provides another important mechanism for generating transient physical crosslinks in aqueous environments. Amphiphilic polymers containing hydrophobic segments can assemble into micellar or clustered domains that act as reversible network junctions. Mihajlovic et al. developed multiblock copolymer hydrogels in which strong hydrophobic association of fatty-acid-derived segments generated mechanically robust supramolecular networks [47]. In drug-delivery systems, such hydrophobic domains can simultaneously provide physical crosslinking and act as reservoirs for poorly water-soluble therapeutic molecules, thereby enhancing loading and reducing rapid diffusion into the surrounding aqueous medium.

Hydrophobic interactions frequently coexist with π–π stacking in peptide- and small-molecule-based hydrogels containing aromatic groups. Zheng et al. demonstrated that the natural small molecule rhein could directly self-assemble into nanofibrous hydrogels through π–π stacking, hydrogen bonding, and related noncovalent interactions [48]. The resulting carrier-free hydrogel exhibited sustained rhein release and prolonged anti-neuroinflammatory activity compared with the free drug. Peptide hydrogels provide another example of cooperative noncovalent assembly. Thota et al. reported an injectable ultrashort peptide hydrogel capable of encapsulating both hydrophilic and hydrophobic compounds and providing controlled release of antitumor agents [49].

Ionic and electrostatic interactions provide an additional strategy for controlling both network structure and cargo transport. Their strength depends strongly on polymer charge density, pH, ionic strength, and the charge of the therapeutic molecule. Dong et al. systematically investigated charged polymers within self-assembling peptide hydrogels and showed that attractive electrostatic interactions increased network crosslinking and mechanical stability while decreasing polymer diffusivity [50]. This study provides direct evidence that electrostatic interactions can couple network organization to the transport of macromolecular cargos. Drug–network electrostatic interactions can similarly regulate small-molecule release. Elsawy et al. investigated doxorubicin transport from β-sheet-forming peptide hydrogels and showed that changes in peptide charge and sequence significantly altered drug retention and release [51]. In addition to electrostatic attraction, hydrophobic and π-associated interactions contributed to drug–fiber association, demonstrating that therapeutic transport in supramolecular networks frequently results from multiple competing molecular interactions rather than a single mechanism.

Overall, supramolecular hydrogels provide a versatile platform in which reversible intermolecular interactions regulate both network dynamics and therapeutic transport. Hydrogen bonding and hydrophobic association can generate reversible physical junctions, host–guest recognition provides molecularly selective network formation and cargo retention, π–π interactions promote self-assembly and drug association, and electrostatic interactions enable charge-dependent control over macromolecular diffusion and small-molecule release. Importantly, these interactions rarely operate independently; instead, their cooperative effects determine network relaxation, cargo affinity, and effective diffusivity. Strong or multivalent interactions may improve structural stability and therapeutic retention but can also delay release, whereas excessively weak interactions may result in rapid network dissociation and burst release. Rational design of supramolecular drug-delivery hydrogels therefore requires balancing the strength and lifetime of network-forming interactions with cargo-specific affinity to achieve the desired duration and mechanism of therapeutic release.

### 2.3. Metal–Ligand Coordination Networks

Metal–ligand coordination provides a versatile strategy for constructing dynamic hydrogel networks because metal ions can reversibly associate with polymer-bound ligands while maintaining relatively high interaction strength. Depending on the metal ion, ligand structure, coordination number, and environmental conditions, these interactions can range from rapidly exchanging transient junctions to comparatively stable crosslinks. Their reversible nature enables hydrogel networks to dissipate mechanical stress, undergo self-healing, and recover following deformation. At the same time, coordination equilibria can respond to pH, competing ligands, metal-ion concentration, and redox conditions, allowing network connectivity and transport properties to be regulated by changes in the local environment.

Among metal-coordinated hydrogels, catechol–metal interactions are particularly well established because they mimic the coordination chemistry found in mussel adhesive proteins. Catechol groups such as 3,4-dihydroxyphenylalanine (DOPA) can coordinate transition-metal ions, especially Fe^3+^, to generate mono-, bis-, or tris-catechol complexes depending strongly on pH and stoichiometry. Holten-Andersen et al. demonstrated that Fe^3+^ coordination with catechol-functionalized polymers produced self-healing networks with elastic moduli approaching those of covalently crosslinked gels [51]. The transition from mono- to bis- and tris-catechol coordination with increasing pH markedly altered network mechanics, demonstrating that coordination state provides a direct molecular mechanism for tuning hydrogel stiffness and relaxation. These findings established catechol–Fe^3+^ interactions as a model system for dynamic metal-coordinated polymer networks.

The kinetics of metal–ligand exchange are equally important because they determine how rapidly the network can rearrange. Grindy et al. systematically varied the identity of metal ions coordinated to catechol-functionalized polymers and showed that metal-dependent bond dynamics could be used to control hydrogel relaxation over multiple timescales [52]. Networks containing different metal–catechol complexes exhibited distinct relaxation behavior even when their overall network structures were similar, demonstrating that the lifetime of coordination bonds can be adjusted independently from network architecture. This concept is particularly relevant to controlled drug delivery because bond lifetime can influence network recovery after injection as well as the time-dependent mobility of encapsulated therapeutics.

Metal coordination is not restricted to catechol ligands. Histidine and other nitrogen-containing ligands can also form reversible complexes with divalent transition-metal ions such as Zn^2+^, Ni^2+^, and Co^2+^. Fullenkamp et al. developed transient hydrogels using histidine-functionalized star PEG polymers coordinated with metal ions and showed that the viscoelastic properties depended strongly on metal identity, pH, polymer concentration, and ionic strength [53]. The reversible nature of histidine–metal coordination enabled transient network behavior and provided a useful means of tuning mechanical relaxation without introducing permanent covalent crosslinks. More recently, Rammal et al. incorporated mussel-inspired metal-binding peptides into microgels and demonstrated reversible aggregation through Zn^2+^ coordination to form self-healing granular hydrogels [54]. Because histidine protonation is pH dependent, the resulting coordination network could be reversibly assembled and disassembled through changes in pH, providing an additional route for environmentally responsive network control.

Metal-ion-induced peptide folding can further couple molecular recognition to hydrogel assembly. Selegård et al. designed a hyaluronic acid–peptide conjugate in which Zn^2+^ coordination induced folding and dimerization of a helix–loop–helix peptide, producing a supramolecular hydrogel [55]. Removal of Zn^2+^ using a chelator caused peptide unfolding and hydrogel disassembly, while changes in Zn^2+^ concentration allowed the mechanical properties and network stability to be modulated. Importantly, the authors also demonstrated release of encapsulated nanoparticles during hydrogel dissociation, illustrating how metal-dependent network assembly can directly influence cargo retention and release [55]. This system highlights the potential of coordination chemistry to function simultaneously as a structural crosslinking mechanism and as a stimulus-responsive regulator of transport.

Catechol–metal coordination has also been directly applied to therapeutic delivery. Yavvari et al. developed an injectable self-healing hydrogel through Fe^3+^ coordination of catechol-containing amphiphilic molecules and used the resulting network for localized combination cancer therapy [56]. The amphiphilic hydrogel accommodated both hydrophilic doxorubicin and hydrophobic docetaxel while retaining self-healing properties after drug loading. Following subcutaneous administration, the hydrogel persisted locally for an extended period, supporting its function as a sustained therapeutic depot. This study demonstrates an important advantage of coordination-driven dynamic networks: reversible metal–ligand crosslinks can provide sufficient cohesion for long-term retention while still allowing injection and structural recovery.

A key design consideration in metal-coordinated hydrogels is the balance between network dynamics and structural persistence. If metal–ligand association is too weak or exchange is too rapid, the hydrogel may exhibit excessive relaxation, erosion, or premature cargo loss. Conversely, very stable coordination complexes may reduce injectability and self-healing and cause the network to behave more like a permanent crosslinked material. Charlet et al. addressed this challenge by designing metal-coordinated hydrogels in which the combination of dynamic coordination and polymer architecture enabled self-healing while preserving macroscopic shape [57]. Such studies emphasize that mechanical stability depends not only on coordination strength but also on ligand density, polymer topology, and the spatial organization of reversible junctions.

The choice of metal ion is additionally important for biomedical applications. Metal identity affects coordination geometry, ligand affinity, exchange kinetics, and responsiveness, while excessive concentrations of transition metals may raise concerns regarding cytotoxicity and long-term tissue accumulation. Consequently, coordination-driven drug-delivery hydrogels require careful optimization of metal concentration, ligand density, and release of free ions. When appropriately designed, however, metal–ligand coordination provides a highly tunable connection between molecular bond dynamics and macroscopic hydrogel behavior. Catechol–Fe^3+^ and histidine–Zn^2+^ systems illustrate how reversible coordination can regulate gelation, relaxation, self-healing, environmental responsiveness, and therapeutic retention. These features make coordination chemistry particularly valuable for smart hydrogel platforms in which dynamic structural adaptation must be balanced with sustained and predictable drug delivery.

### 2.4. Hybrid Dynamic Networks

Hybrid dynamic networks integrate two or more crosslinking mechanisms within a single hydrogel to overcome the limitations associated with individual dynamic interactions. Although rapidly exchanging bonds can provide injectability, self-healing, and environmental responsiveness, they may also result in excessive network relaxation, weak mechanical properties, or premature cargo loss. Conversely, more stable crosslinks can improve structural integrity and therapeutic retention but may restrict network rearrangement. Combining interactions with different strengths and characteristic lifetimes therefore provides a strategy for independently tuning short-term adaptability and long-term stability. Hybrid designs may involve covalent–noncovalent dual crosslinking, interpenetrating polymer networks (IPNs), or multiple dynamic crosslinks with distinct exchange mechanisms.

The combination of dynamic covalent and noncovalent interactions is particularly attractive because the two mechanisms can perform complementary functions. Han et al. developed a dual-crosslinked hydrogel based on methacrylated chitosan and oxidized hyaluronic acid, in which rapid electrostatic attraction between oppositely charged polymer groups cooperated with dynamic Schiff base formation [58]. The resulting network exhibited rapid gelation, shear-induced flow, and recovery after deformation. Subsequent photopolymerization further reinforced the hydrogel, illustrating how sequential dynamic and permanent crosslinking can separate the requirements for injectability during administration from those for mechanical stability after placement. Such multistage network formation is relevant to therapeutic delivery because an initially dynamic network can facilitate minimally invasive injection, whereas secondary stabilization may reduce erosion and improve retention at the target site.

A related strategy combines multiple reversible bonds rather than introducing a permanent secondary network. As discussed above, Liu et al. constructed a doubly dynamic hydrogel using boronate ester and acylhydrazone bonds [59]. The relatively rapid exchange of boronate ester crosslinks supported gelation and self-healing, whereas the more persistent acylhydrazone linkages contributed to network stability. By incorporating crosslinks with different exchange kinetics, such systems can generate multiple relaxation timescales within the same material. This concept is particularly useful for controlled drug delivery, where rapid structural recovery after injection must often be reconciled with slower network remodeling during prolonged therapeutic release.

Interpenetrating polymer networks provide another approach to integrating distinct material functions. In an IPN, two polymer networks are formed within the same physical space and become topologically interlaced, allowing each network to retain characteristic properties while contributing cooperatively to the bulk material. Gilbert et al. developed fully injectable IPN hydrogels through simultaneous formation of hydrazone-crosslinked poly(N-isopropylacrylamide) and thiosuccinimide-crosslinked poly(N-vinylpyrrolidone) networks using kinetically orthogonal reactions [60]. The resulting IPNs exhibited synergistically enhanced mechanical properties and distinct degradation, pore structure, and thermal swelling behavior compared with the corresponding individual networks. This study demonstrates that the sequence and kinetics of network formation are important design parameters because independently reacting networks can generate structural properties that cannot be achieved simply by increasing the crosslinking density of a single polymer.

The potential of IPN architectures for drug delivery was demonstrated by Zhang et al. using sericin and alginate double networks [61]. Compared with alginate alone, the sericin–alginate IPNs exhibited improved mechanical strength and degradation stability while supporting sustained drug release. Importantly, variation in the composition altered network properties and release behavior, illustrating how interpenetrating architectures can be used to decouple mechanical reinforcement from molecular transport. In such systems, therapeutic release is influenced not only by the chemistry of individual crosslinks but also by the spatial organization, porosity, and interactions between the interpenetrated networks.

Hybrid networks can also be created by combining polymeric matrices with self-assembled supramolecular structures. Nakamura et al. investigated composite hydrogels consisting of polymer networks and supramolecular nanofibers and identified several distinct network arrangements, including orthogonal and interacting architectures [62]. The resulting structures were determined by the order of network formation and the interactions between the constituent fibers, and some systems exhibited time-dependent network remodeling. These observations emphasize that hybrid hydrogel behavior depends not only on the chemical identity of individual crosslinks but also on how multiple networks are spatially organized. For drug delivery, this principle is important because one network may primarily provide mechanical support, whereas another may contribute stimuli responsiveness or affinity-based cargo retention.

Nanostructured components can similarly function as dynamic crosslinking elements while serving as therapeutic carriers. Liang and Kiick developed liposome-crosslinked hybrid hydrogels in which liposomes acted as multifunctional junctions within a PEG-based polymer network [63]. The liposomes simultaneously contributed to network formation and served as reservoirs for encapsulated cargos, while glutathione-responsive chemistry enabled triggered network degradation and release. This architecture illustrates how hybrid networks can integrate structural and delivery functions at different length scales, providing opportunities to control the release of multiple therapeutic agents through both carrier destabilization and hydrogel-network remodeling.

More complex dynamic systems may incorporate several independently addressable interactions to enable programmable transitions between network states. Yue et al. developed DNA-based constitutional dynamic network hydrogels in which the relative populations of different crosslinking constituents could be reversibly reconfigured using molecular triggers [64]. The hydrogels could be switched among low-, intermediate-, and high-stiffness states, and these structural transitions were used to regulate the release of doxorubicin and nanoparticle cargos. Increased network stiffness reduced cargo release, whereas trigger-induced conversion to a low-stiffness state promoted release [64]. This study demonstrates a more advanced form of hybrid dynamic design in which therapeutic transport is controlled through programmed reorganization of the network rather than through simple cleavage or degradation of a single responsive bond.

Overall, hybrid dynamic networks provide a means of integrating complementary molecular interactions across different kinetic and structural scales. Covalent–noncovalent combinations can balance rapid recovery with structural persistence, IPNs can combine mechanically and chemically distinct polymer networks, and multiple dynamic crosslinks can introduce hierarchical or independently controllable relaxation processes. For controlled drug delivery, the principal advantage of these architectures is the possibility of separating functions that are difficult to optimize simultaneously within a single network, including injectability, mechanical stability, stimuli responsiveness, cargo affinity, degradation, and sustained release. However, increasing network complexity also introduces challenges in predicting crosslink kinetics, spatial organization, and transport pathways. Understanding how these interacting networks collectively determine hydrogel structure and dynamics is therefore essential for establishing the structure–property relationships discussed in the following section. The major dynamic crosslinking strategies discussed above and their key characteristics relevant to controlled drug delivery are summarized in Table 1. Taken together, no single dynamic interaction provides an optimal solution for all drug-delivery requirements. Dynamic covalent networks generally offer greater structural persistence and tunable chemical responsiveness, but slower exchange or reaction-dependent conditions can limit rapid adaptation. Supramolecular networks provide excellent injectability and self-recovery but may be more sensitive to dilution, competing molecules, or weak cargo retention. Metal–ligand systems offer highly tunable bond lifetimes, although metal-ion release and coordination stability require careful control. Hybrid networks can combine complementary advantages, but increasing compositional and structural complexity may reduce predictability and reproducibility. Thus, selection of a dynamic crosslinking strategy should be based on the required balance among network stability, relaxation, cargo retention, responsiveness, and practical formulation complexity rather than dynamic behavior alone.

## 3. Structure–Property Relationships in Dynamic Hydrogels

Dynamic crosslinks translate molecular exchange kinetics into time-dependent network properties, including effective crosslink density, mesh accessibility, swelling, viscoelasticity, and permeability [7,24,25]. These evolving properties govern the physical constraints experienced by therapeutic cargos and connect molecular bond dynamics with transport behavior [27]. The following subsections examine the key structure–property relationships relevant to controlled drug delivery.

### 3.1. Crosslinking Density and Network Architecture

Crosslinking density is one of the primary structural parameters governing the mechanical and transport properties of hydrogel networks. Increasing the number of elastically active crosslinks generally shortens the average polymer strand between junctions, restricts chain motion, increases network stiffness, and limits swelling and molecular transport [7,25]. In conventional permanently crosslinked hydrogels, crosslinking density is largely established during network formation and remains relatively fixed unless the network undergoes degradation. In dynamic hydrogels, however, the effective crosslinking density can evolve with time because reversible junctions continuously dissociate and reform. Therefore, the macroscopic behavior of a dynamic network depends not only on the number of crosslinks but also on their lifetimes, exchange kinetics, and probability of reformation. This distinction is important because two hydrogels with similar initial moduli may exhibit different relaxation, recovery, and transport properties when the underlying crosslinks exchange at different rates [24].

Dynamic covalent systems provide clear examples of how crosslink density and bond kinetics can be coupled. FitzSimons et al. investigated tetra-PEG hydrogels crosslinked through reversible thia-Michael addition and demonstrated that changes in pH altered both the forward and reverse reaction rates as well as the equilibrium of the dynamic crosslinks [66]. Increasing pH accelerated bond exchange and produced faster stress relaxation and lower hydrogel stiffness, illustrating that the effective connectivity of a dynamic network cannot be described solely by the nominal concentration of reactive groups. Similarly, Morgan et al. showed that crosslinker length, valency, and reaction kinetics strongly affected gel formation and mechanical properties in hydrazone-based dynamic networks [24]. Increasing crosslink functionality enhanced network formation, whereas changes in molecular architecture altered the efficiency with which potential crosslinking sites became mechanically active. These findings indicate that the effective crosslink density of dynamic hydrogels reflects both chemical stoichiometry and the kinetics of reversible bond formation.

Network connectivity can also be regulated after or during gel formation through competitive dynamic interactions. Gilchrist et al. introduced transient molecular competitors into dynamically crosslinked recombinant protein hydrogels and demonstrated that competition for reactive binding sites could reversibly modify network formation, stiffness, and stress-relaxation behavior [67]. Rather than permanently changing polymer composition, this strategy altered the fraction of crosslinks participating in the percolated network. Such approaches are particularly relevant to smart hydrogels because they provide a means of adjusting network connectivity through molecular cues without requiring irreversible cleavage of the polymer backbone. From a drug-delivery perspective, stimulus-dependent changes in effective crosslink density could therefore alter both mechanical stability and the accessibility of transport pathways within the same material.

An ideal hydrogel is often represented as a uniform network in which crosslinks are evenly distributed and all polymer strands contribute equally to elasticity. Real polymer networks, however, contain structural heterogeneities such as loops, dangling chains, locally over-crosslinked regions, and areas of reduced connectivity. These defects can cause the experimentally effective crosslink density to differ substantially from that predicted from the amount of crosslinker used during synthesis. Zhong et al. quantified loop defects in model polymer hydrogels and demonstrated that even well-defined networks contained topological defects that significantly reduced bulk elasticity [68]. Their findings established that network architecture, rather than crosslink concentration alone, must be considered when relating molecular structure to macroscopic properties. Although this study involved predominantly permanent networks, the principle is particularly relevant to dynamic hydrogels, where reversible association and dissociation can continuously redistribute network connectivity.

Structural heterogeneity also has direct consequences for molecular transport. Rose et al. investigated nanoparticle dynamics in tetra-PEG hydrogels containing systematically introduced network defects [69]. In nearly homogeneous networks, nanoparticles whose dimensions approached the characteristic mesh size were largely immobilized. As defects were introduced, the proportion of mobile particles and their displacement increased, indicating that locally enlarged transport pathways can markedly affect cargo mobility even when the average network structure changes modestly. Importantly, particle size determined how strongly the probes responded to network defects, emphasizing that the influence of heterogeneity depends on the length scale of the transported species [69]. For therapeutic delivery, this means that an apparently similar bulk hydrogel may exhibit substantially different transport behavior for small molecules, proteins, nanoparticles, or other macromolecular cargos depending on the spatial distribution of local network defects.

The relationship between connectivity and local transport becomes even more complex in transient networks. Nakamura et al. used model tetra-PEG transient networks with precisely controlled connectivity to investigate particle dynamics near the sol–gel transition [70]. As network connectivity approached the percolation threshold, the distribution of particle mobility became increasingly heterogeneous, reflecting the coexistence of locally connected network regions and relatively mobile regions. Above the percolation threshold, probe particles increasingly exhibited restricted or subdiffusive motion. These observations demonstrate that dynamic network architecture can generate spatially heterogeneous microenvironments even when the constituent polymers and crosslink chemistry remain unchanged. Thus, average parameters such as bulk modulus or nominal crosslink density may not fully describe the local environment experienced by therapeutic molecules.

Polymer-chain mobility provides the molecular connection between network architecture and time-dependent material behavior. Increasing effective crosslink density generally restricts segmental and large-scale chain motion, whereas reversible crosslinks permit chains to rearrange once junctions dissociate. The characteristic timescale of this rearrangement depends on both crosslink lifetime and polymer architecture. Rijns et al. compared supramolecular hydrogels assembled from molecular motifs with different exchange dynamics and demonstrated that molecular-scale monomer exchange was translated into differences in bulk stress relaxation [71]. Slow molecular exchange produced more persistent networks, whereas rapidly exchanging supramolecular structures generated more dynamic bulk materials. These observations reinforce the concept that chain mobility in dynamic hydrogels is governed not simply by how many crosslinks are present, but by how long individual junctions constrain polymer motion.

Crosslink density, network heterogeneity, and chain mobility should therefore be regarded as interdependent rather than independent structural parameters. A highly connected network generally provides greater mechanical stability and stronger cargo confinement, but excessive connectivity can suppress polymer rearrangement and reduce the adaptive behavior required for self-healing or stimulus responsiveness. Conversely, low or rapidly exchanging connectivity increases chain mobility and network relaxation but may promote excessive swelling, erosion, or premature cargo loss. Spatial heterogeneity can further create preferential transport pathways that are not captured by bulk measurements. For controlled drug delivery, rational design consequently requires optimization of both the average network connectivity and its spatial and temporal distribution. These structural features determine the physical framework within which mesh size, swelling, viscoelastic relaxation, and ultimately therapeutic transport are established.

### 3.2. Mesh Size, Swelling, and Water Content

Mesh size is a key structural parameter that determines the space available for molecular motion within a swollen hydrogel network. It is commonly interpreted as a characteristic distance associated with the polymer strands between neighboring network junctions and is influenced by polymer concentration, crosslinking density, chain length, network functionality, and swelling state [72]. As the network absorbs water, polymer chains separate and the effective mesh generally expands, whereas increased crosslinking or network contraction restricts chain separation and decreases the accessible space for solute transport. The relationship between mesh size and cargo dimensions is therefore particularly important for controlled drug delivery. Small molecules that are substantially smaller than the characteristic mesh can diffuse relatively freely through the aqueous phase, whereas proteins, polymers, and nanoparticles with dimensions approaching the mesh size experience increasing steric restriction. When cargo dimensions exceed the characteristic openings of the network, transport may require network relaxation, degradation, or the presence of larger local defects rather than simple Fickian diffusion [72].

Swelling provides an important connection between hydrogel composition and internal network dimensions. Water uptake is driven by the thermodynamic tendency of hydrophilic polymer chains to interact with the surrounding solvent, while elastic forces generated by network crosslinks oppose unlimited expansion. Consequently, equilibrium swelling reflects a balance between polymer–solvent mixing and elastic resistance of the network. Richbourg et al. systematically examined multiple synthetic hydrogel formulations and demonstrated quantitative relationships between synthesis variables and swelling behavior [73]. Changes in initial polymer concentration and the degree of polymerization between crosslinks altered the swollen polymer volume fraction and thereby affected predicted stiffness and solute diffusivity. These results demonstrate that swelling measurements can provide valuable information about network structure, particularly in well-defined synthetic hydrogels. However, such relationships become more complex in heterogeneous biopolymer or dynamic networks, where reversible crosslinking, chain association, and network defects may violate assumptions used in classical equilibrium-swelling models.

The influence of mesh size on molecular diffusion has been demonstrated directly using solutes with different dimensions. Richbourg and Peppas measured the diffusion of fluorescein, dextrans, and PEG molecules in structurally varied poly(vinyl alcohol) hydrogels using fluorescence recovery after photobleaching [74]. Increasing the hydrogel mesh radius generally increased solute diffusivity, confirming the importance of network dimensions for molecular transport. Nevertheless, solutes with similar hydrodynamic dimensions did not necessarily exhibit identical diffusion behavior, indicating that molecular size alone does not fully determine accessibility. Rehmann et al. reached a similar conclusion in PEG-based step-growth hydrogels designed for protein delivery [75]. Hydrogels with estimated mesh sizes of approximately 5–15 nm showed predictable retention of proteins substantially larger than the mesh and relatively rapid release of smaller proteins, whereas proteins whose dimensions were comparable to the mesh exhibited hindered diffusion that required experimental characterization. Thus, the ratio between cargo dimensions and network mesh provides a useful first approximation of transport behavior, but it cannot completely account for molecular shape, flexibility, and polymer–cargo interactions.

More detailed studies have further shown that mesh size alone may oversimplify the three-dimensional geometry of transport pathways. Richbourg and Peppas investigated solute diffusion and partitioning across a library of multi-arm PEG hydrogels in which several network structural parameters were varied independently [76]. Their results demonstrated that junction functionality influenced solute diffusivity even when conventional mesh-size descriptions predicted similar transport environments. The authors therefore emphasized the importance of network geometry and introduced mesh radius as a more representative descriptor of the accessible three-dimensional opening through which a solute must pass. Importantly, swelling ratio, diffusivity, and solute partitioning were not universally correlated. This observation cautions against using equilibrium swelling alone as a direct surrogate for drug transport and suggests that both network architecture and cargo-specific properties should be evaluated when predicting release.

The terms mesh size and porosity should also be distinguished when describing hydrogel structure. Mesh size generally refers to nanoscale dimensions created by molecular polymer-network constraints, whereas hydrogel porosity may additionally include larger voids or defects at the micro- to macroscale. These larger pores can originate from polymerization heterogeneity, phase separation, fabrication methods, or network defects and can create preferential pathways for molecular or particulate transport. Tolentino et al. recently compared native-state particle tracking with cryogenic scanning electron microscopy in PEG hydrogels and demonstrated the presence of micron-scale pores that were distinct from the nanoscale mesh sizes estimated from equilibrium swelling [77]. The study also highlighted that imaging methods involving freezing, drying, or other sample preparation may alter apparent pore morphology. Accordingly, characterization of hydrogel transport should differentiate molecular mesh dimensions from larger-scale porosity, particularly when therapeutic nanoparticles, cells, or macromolecular assemblies are involved.

Water content further affects solute accessibility by controlling both polymer volume fraction and the physical state of the aqueous phase within the network. Highly swollen hydrogels generally contain larger aqueous domains and reduced polymer obstruction, which can facilitate diffusion of freely soluble cargos. However, high water content does not necessarily result in proportionally rapid transport because solutes may interact with polymer chains or become sterically restricted by network geometry. This complexity was demonstrated recently in calcium-alginate hydrogels, where Liu et al. combined spatiotemporal fluorescence measurements with computational diffusion modeling [78]. Increasing alginate concentration decreased swelling and mesh size and correspondingly reduced dextran diffusivity. Experimental diffusion coefficients were nevertheless affected by network microheterogeneity and solute characteristics, again showing that molecular transport cannot be predicted from a single structural parameter.

In dynamic hydrogels, swelling and mesh accessibility can evolve as reversible crosslinks dissociate or environmental conditions shift the network equilibrium. pH-dependent boronate ester systems illustrate how these changes can simultaneously alter rheology and drug transport [34].

The practical importance of controlling water uptake and mesh structure is evident in drug-release systems. Christian et al. varied the crosslinker content of hydrogel-forming poly(HEMA-co-EGDMA) coatings and showed that increasing crosslink density reduced swelling and calculated mesh size while markedly altering indomethacin release [79]. Drug liberation could be extended from rapid dissolution to release over hours or days by modifying the hydrogel coating composition. Such findings illustrate how network design can translate structural changes in water uptake and molecular accessibility into therapeutically relevant release profiles. Overall, mesh size, porosity, swelling, and water content are closely connected but non-equivalent properties. Their combined consideration is necessary for understanding how therapeutic cargos access and migrate through dynamic hydrogel networks and provides the structural basis for the viscoelastic and time-dependent transport phenomena discussed in the following sections.

### 3.3. Viscoelasticity and Network Relaxation

Viscoelasticity is a defining characteristic of dynamic hydrogels because reversible crosslinks allow the network to display both elastic energy storage and time-dependent energy dissipation. Oscillatory rheology is commonly used to characterize these behaviors through the storage modulus (G′) and loss modulus (G″). G′ represents the elastic contribution of the network, whereas G″ reflects viscous dissipation associated with polymer motion, crosslink rearrangement, and other relaxation processes. In a predominantly solid-like hydrogel, G′ generally exceeds G″ over the experimentally relevant frequency range, while an increased G″ or a frequency-dependent crossover between G′ and G″ indicates greater network mobility and relaxation. Unlike permanently crosslinked networks, dynamic hydrogels may display pronounced frequency dependence because reversible junctions can appear effectively permanent at timescales shorter than their bond lifetime but relax when deformation is applied over longer timescales [25,71]. Thus, rheological measurements provide an important link between molecular exchange processes and bulk network dynamics.

The elastic and viscous components of dynamic hydrogels can often be tuned independently to some extent. In ionically crosslinked alginate hydrogels, Chaudhuri et al. demonstrated that stress relaxation could be varied while maintaining similar initial elastic moduli, establishing that stiffness and viscoelasticity represent distinct material properties [20]. Nam et al. subsequently modified alginate with PEG chains and showed that increasing PEG content accelerated stress relaxation and increased the loss modulus without substantially changing the initial elastic modulus [80]. These studies emphasize that G′ alone is insufficient to characterize dynamic hydrogel mechanics. Two networks with comparable initial stiffness may dissipate mechanical stress at very different rates because of differences in polymer-chain mobility, crosslink rearrangement, or network architecture.

Stress-relaxation measurements provide a more direct assessment of this time-dependent behavior. In a typical stress-relaxation experiment, a rapid deformation is imposed and maintained while the decrease in stress is monitored over time. Permanently crosslinked elastic networks retain a substantial fraction of the initial stress, whereas transient networks progressively dissipate stress as reversible junctions dissociate and polymer chains reorganize. The rate and extent of this decay depend on crosslink lifetime, polymer connectivity, entanglements, and the presence of multiple relaxation mechanisms. Consequently, relaxation time is frequently used as a characteristic measure of how rapidly the network reorganizes. Depending on the hydrogel and fitting method, it may be reported as the time required for stress to decrease to a defined fraction of its initial value, as a half-relaxation time, or as a characteristic time obtained from single- or multi-mode viscoelastic models. These definitions should therefore be specified when relaxation times are compared across different hydrogel systems.

Among the most important molecular determinants of stress relaxation is the exchange kinetics of reversible crosslinks. Lou et al. provided a quantitative demonstration of this relationship using hydrazone-crosslinked dynamic covalent hydrogels [81]. By varying catalyst concentration, they changed the hydrazone exchange rate while maintaining approximately constant crosslink density, allowing the effects of bond kinetics and network structure to be separated. The terminal relaxation time was governed primarily by the crosslink exchange rate and the number of effective crosslinks per polymer chain, and a quantitative correlation was established between molecular exchange kinetics and macroscopic stress relaxation. This study provides an important molecular basis for designing dynamic hydrogels with predictable viscoelastic behavior rather than adjusting relaxation empirically.

Environmental factors can similarly regulate relaxation by altering dynamic bond kinetics. FitzSimons et al. showed that pH modified both the equilibrium and kinetics of reversible thia-Michael crosslinks, producing corresponding changes in hydrogel stiffness and stress-relaxation behavior [66]. In metal–ligand networks, coordination kinetics provide another mechanism for controlling characteristic relaxation times. Grindy et al. studied tetra-arm PEG networks reversibly crosslinked through histidine–Ni^2+^ coordination and demonstrated that polymer-network structure primarily determined static elasticity, whereas coordination-bond rearrangement strongly influenced time-dependent relaxation [82]. Earlier work using different metal–catechol complexes likewise showed that changing metal identity could alter coordination dynamics and generate distinct hierarchical relaxation behavior [38]. These findings demonstrate that chemical selection of a dynamic crosslink provides a direct route for programming the timescale over which a hydrogel transitions from predominantly elastic to dissipative behavior.

Crosslink architecture also influences viscoelasticity even when the same dynamic chemistry is used. Lin et al. recently investigated dynamic covalent hydrogels with different crosslinker architectures and showed that the arrangement of reversible junctions affected both stiffness and stress relaxation [83]. Differences in crosslinker topology altered the effective number and distribution of elastically active junctions and therefore changed the relationship between bond exchange and macroscopic relaxation. Similar behavior has been observed in supramolecular systems, where molecular exchange dynamics can be translated into distinct bulk relaxation responses depending on polymer organization [71]. Thus, relaxation time cannot always be predicted from the lifetime of an isolated dynamic bond alone; network connectivity, polymer architecture, and cooperative association must also be considered.

The importance of matching molecular and macroscopic timescales becomes especially apparent under flow. Crowell et al. investigated injectable tetra-PEG hydrogels crosslinked by dynamic thia-Michael addition bonds and showed that bond-exchange kinetics and polymer concentration could be used to tune plateau moduli and relaxation times [84]. The hydrogels exhibited relaxation times on the order of seconds and showed shear-dependent behavior associated with the transient nature of the network. Such rapid relaxation is advantageous for injectable materials because temporary network rearrangement can permit flow through a needle, followed by recovery of the crosslinked structure after administration. However, excessively rapid relaxation may also increase polymer mobility and facilitate cargo escape, highlighting the need to balance injectability with therapeutic retention.

Dynamic control of viscoelasticity can also be introduced after hydrogel formation. Crandell and Stowers used light-triggered PEG conjugation within ionically crosslinked alginate hydrogels to alter stress-relaxation rates without substantially changing the elastic modulus [85]. This approach demonstrates that the relaxation behavior of a formed hydrogel can be modified spatially and temporally rather than being fixed during synthesis. More broadly, rapid energy dissipation can also arise from dynamic molecular motion other than conventional bond exchange. Xu et al. engineered hydrogels in which sliding molecular interactions generated strong molecular friction and rapid mechanical damping [86]. As illustrated in Figure 3, the engineered chain-walker architecture enables cyclodextrin rings to slide along PEG chains during deformation and subsequently recover their positions by diffusion, thereby coupling rapid energy dissipation with reversible network relaxation. These examples highlight the increasing ability to regulate viscoelastic properties through molecular processes that can be independently controlled in space and time.

For controlled drug delivery, viscoelastic relaxation is important because it governs how rapidly the polymer network can rearrange around an encapsulated therapeutic cargo. Slowly relaxing networks maintain persistent physical constraints and can favor prolonged retention, whereas faster relaxation increases polymer-chain mobility and may generate transient pathways for diffusion. Appel et al. demonstrated that crosslink dynamics can substantially alter cargo release even in physically crosslinked hydrogels with otherwise comparable structural properties [22]. More recently, evolving relaxation and transport properties in dynamic hydrogels were shown to affect both early-stage and long-term cargo release [27]. Accordingly, G′, G′′, stress-relaxation behavior, and characteristic relaxation times should be considered together when evaluating dynamic hydrogels for drug delivery. These parameters provide a mechanistic bridge between molecular crosslink kinetics and the time-dependent transport processes discussed in subsequent sections.

### 3.4. Self-Healing and Injectable Behavior

Self-healing and injectability are characteristic macroscopic consequences of reversible network dynamics and are particularly valuable for hydrogel-based drug delivery. When a dynamic hydrogel is mechanically disrupted, reversible crosslinks can dissociate and subsequently reform across the damaged interface, allowing the network to recover its structural and rheological properties. This self-recovery process distinguishes dynamic hydrogels from permanently crosslinked networks, in which mechanical damage generally results in irreversible fracture. The efficiency of self-healing depends on several interconnected parameters, including polymer-chain mobility, crosslink density, bond lifetime, and the rates of crosslink dissociation and reassociation. Consequently, rapid bond exchange generally promotes faster recovery, whereas highly persistent junctions can restrict chain rearrangement and slow interfacial healing [28].

Self-recovery is commonly evaluated using macroscopic cut-and-heal experiments together with cyclic rheological measurements in which the hydrogel is alternately exposed to low and high strains. During high-strain deformation, the transient network is disrupted and G′ may decrease below G′′, indicating a transition toward a more fluid-like state. When the strain is returned to the linear viscoelastic regime, reformation of reversible crosslinks restores G′ and the solid-like network. Sun et al. developed dynamically crosslinked protein hydrogels based on hetero-coiled-coil interactions that recovered their mechanical properties within seconds after shear deformation [87]. Similarly, Wang et al. demonstrated this behavior in oxaborole–diol-crosslinked hydrogels, where dynamic covalent exchange provided both self-healing and rapid recovery after repeated mechanical disruption [88]. These studies illustrate that self-healing is fundamentally governed by the ability of polymer chains and dynamic junctions to reorganize on experimentally relevant timescales.

Self-healing and shear thinning arise from reversible junction disruption under stress followed by reassociation after stress removal. This behavior has been demonstrated across guest–host, polymer–nanoparticle, hydrazone, oxaborole–diol, and thia-Michael networks [17,18,19,84,88].

Shear thinning provides the rheological basis for minimally invasive injection of preformed hydrogels. Rather than relying exclusively on low-viscosity precursors that undergo gelation after administration, dynamic hydrogels can exist as cohesive networks before delivery, temporarily flow through a needle or catheter under high shear, and subsequently recover after reaching the target site. This approach can reduce uncertainties associated with in situ polymerization and may improve spatial retention of encapsulated therapeutics. Pérez-San Vicente et al. demonstrated this concept using a dynamic hydrogel based on gold(I)–thiolate/disulfide exchange [89]. The preformed hydrogel could be injected while undergoing temporary network disruption and subsequently recovered its mechanical integrity and microstructure, enabling its evaluation as an injectable nucleus pulposus replacement. More recently, Tsai et al. developed benzaldehyde-functionalized chitosan hydrogels in which reversible Schiff base linkages produced pronounced shear-thinning, self-healing, and injectable behavior [90]. Together, these studies demonstrate that the characteristic timescale of crosslink reformation must be sufficiently short to restore network cohesion after injection.

Injectability nevertheless involves more than a decrease in viscosity under shear. The hydrogel must experience sufficient flow to pass through the selected needle diameter without requiring excessive injection force while retaining adequate recovery to avoid spreading or erosion after administration. These requirements can be competing: increasing crosslink density or bond lifetime generally improves post-injection stability but may increase the force required for delivery, whereas excessively rapid crosslink dissociation can facilitate injection but reduce retention. Thus, the rheological parameters controlling injectability should be considered together with the relaxation behavior described in Section 3.3. Ideally, the network relaxation time should permit rearrangement during the relatively short period of injection but enable sufficiently rapid reassembly after the shear field is removed. Dynamic crosslink concentration, polymer molecular weight, and association kinetics therefore provide important variables for balancing injection force and post-injection recovery.

An additional advantage of injectable dynamic hydrogels is their ability to conform to irregular anatomical spaces. Because transient networks can flow under applied stress and subsequently reconstruct, they can adapt to defects with complex geometries rather than retaining a predetermined macroscopic shape. This shape adaptability should be distinguished from conventional shape-memory behavior; it arises primarily from reversible flow and network reconstruction rather than recovery of a preprogrammed geometry. Guo et al. developed a multiple-dynamic-bond hydrogel containing reversible borate–diol interactions, hydrogen bonding, and Schiff base linkages that combined injectability, self-healing, tissue adhesion, and shape adaptability [91]. Such behavior is particularly useful for wound cavities, postsurgical spaces, joints, and other anatomically irregular sites where intimate contact between a therapeutic depot and surrounding tissue can improve local retention and reduce formation of unfilled spaces.

The relationship among dynamic network recovery, injectability, and therapeutic delivery has become increasingly evident in recent drug-delivery systems. Qin et al. recently developed an injectable hydrogel containing dynamic Schiff base and boronate ester bonds together with bevacizumab-loaded bioactive glass nanoparticles [92]. The network displayed rapid gelation, shear-thinning, injectability, and self-healing, while its pH- and ROS-responsive dynamic crosslinks enabled gradual therapeutic release in an osteoarthritis model. This example illustrates how the same reversible interactions can simultaneously determine administration behavior and therapeutic transport. Rather than considering injectability solely as a processing characteristic, dynamic network recovery should therefore be regarded as part of the overall drug-delivery mechanism because post-injection reconstruction determines cargo confinement, local residence, and subsequent release.

Overall, self-healing, shear-thinning, injectability, and shape adaptability arise from a common molecular principle: reversible junctions permit temporary network disruption while maintaining the capacity for subsequent reconstruction. Their performance is governed by the relationship between applied deformation and the characteristic timescales of bond exchange, polymer-chain motion, and network relaxation. Rapid rearrangement favors injection and conformability, whereas sufficient crosslink persistence is required to preserve structural integrity and therapeutic retention after administration. Rational design of injectable dynamic hydrogels therefore requires balancing these competing timescales so that the material flows when required, rapidly recovers at the target site, conforms to the surrounding geometry, and subsequently provides a stable environment for controlled therapeutic release.

### 3.5. Degradation and Erosion

Degradation and erosion are important time-dependent processes that determine the structural persistence and therapeutic-release behavior of dynamic hydrogels. Although the terms are sometimes used interchangeably, they describe distinct phenomena. Degradation refers to cleavage of polymer backbones, crosslinks, or other network-forming junctions, whereas erosion represents the actual loss of soluble polymer fragments from the hydrogel matrix. Bond cleavage can therefore occur without immediate macroscopic mass loss until network connectivity decreases sufficiently for polymer fragments to diffuse into the surrounding medium. In drug-delivery systems, these processes progressively alter crosslink density, mesh size, swelling, mechanical properties, and solute accessibility, thereby coupling network disassembly to cargo transport. The degradation rate must consequently be matched to the desired therapeutic timescale because rapid network breakdown can cause premature cargo loss, whereas excessively slow degradation may result in incomplete release or prolonged persistence of the implanted material.

Hydrolytic degradation is commonly introduced through water-sensitive linkages within polymer backbones or crosslinks. Ester-containing segments are particularly useful because their cleavage can gradually reduce molecular weight and network connectivity under physiological aqueous conditions. Pertici et al. developed an injectable thermoresponsive hydrogel containing hydrolyzable polyester segments and showed that polymer degradation was directly associated with progressive drug release [93]. Importantly, cargo release occurred without a pronounced initial burst and was correlated with polymer mass loss, demonstrating how hydrolysis can be engineered to provide gradual therapeutic delivery. Hydrolytic degradation rates can be controlled through the chemical structure and density of degradable groups, polymer hydrophobicity, crosslinking density, and water accessibility. As discussed in Section 3.2, network swelling and mesh structure are therefore closely connected to degradation because they determine how readily water penetrates and accesses hydrolytically labile linkages.

More precise control can be achieved by incorporating chemically programmable cleavable crosslinks. Ashley et al. developed PEG hydrogels containing β-eliminative linkers whose cleavage rates could be systematically tuned through linker structure [94]. The same chemistry was used both to attach therapeutic molecules and to regulate network degradation, allowing drug release and hydrogel erosion to be programmed over a wide range of timescales. This study demonstrated an important principle for degradable delivery systems: cargo liberation and bulk network disappearance do not necessarily need to occur simultaneously. By independently adjusting drug-linker and network-crosslinker cleavage kinetics, therapeutic release can be completed before substantial erosion of the hydrogel depot. Such temporal separation is particularly useful when prolonged delivery is required without premature loss of structural integrity.

Enzymatic degradation provides an alternative strategy in which biologically active enzymes selectively cleave peptide or polymer sequences incorporated into the network. Because enzyme concentrations often vary with tissue type, inflammation, remodeling, or disease progression, enzymatically degradable hydrogels can convert local biological activity into adaptive changes in network structure. Guo et al. developed PEG hydrogels crosslinked with an MMP-8-cleavable peptide sequence for localized intraoral drug delivery [95]. Exposure to MMP-8 promoted network degradation and enhanced release of minocycline, bovine serum albumin, and an antimicrobial peptide, demonstrating enzyme-dependent control over the release of cargos with different molecular characteristics. The system illustrates how disease-associated protease activity can serve as an endogenous trigger for on-demand hydrogel degradation and therapeutic delivery.

The relationship between enzymatic cleavage, bulk degradation, and molecular release depends strongly on both network structure and cargo dimensions. Wu and Schultz quantitatively investigated PEG–peptide hydrogels containing enzymatically degradable crosslinks and correlated bulk degradation with molecular release [96]. Their results demonstrated that degradation-mediated transport depended on the relative dimensions of the network mesh, enzyme, and released species. Cargo that was strongly confined within the initial network became increasingly mobile as enzymatic cleavage reduced network connectivity and enlarged accessible transport pathways. Tanimoto et al. similarly showed that the relationship among hydrogel mesh size, enzyme size, and cargo size controlled both enzymatic degradation and release behavior [97]. By varying the network structure, they obtained hydrogels with tunable degradation and dynamic mechanical properties, emphasizing that enzyme accessibility should be considered together with cleavage chemistry when designing responsive delivery matrices.

Dynamic bond dissociation can remodel a network without immediate irreversible degradation; erosion begins when dissociation and fragment escape exceed network reassociation. Boronate ester and slowly reversible Diels–Alder systems illustrate how changes in crosslink stability can couple network disassembly to therapeutic release [23,34,41].

Network erosion may occur predominantly throughout the hydrogel volume or near its external interface. During bulk erosion, cleavage proceeds throughout the hydrated network, and macroscopic integrity may be maintained until connectivity approaches a critical threshold, after which rapid mass loss can occur. In contrast, surface-dominated erosion progressively removes material from the hydrogel boundary while the interior remains relatively intact. Which regime dominates depends on the relative rates of water or enzyme penetration, bond cleavage, polymer-fragment diffusion, and network reformation. For dynamic hydrogels, these processes are further complicated by reversible exchange because transiently detached chains may either rejoin the network or escape into the surrounding medium. Consequently, erosion kinetics cannot be inferred solely from the intrinsic dissociation rate of an isolated dynamic bond.

Overall, degradation and erosion integrate several of the structure–property relationships described in the preceding sections. Crosslink density and heterogeneity determine the number and spatial distribution of cleavable junctions, mesh size and swelling govern access of water and enzymes, and network relaxation influences the ability of dissociated polymer chains to rearrange or escape. Dynamic bond cleavage further introduces competition between crosslink dissociation and reassociation. For controlled drug delivery, the optimal network should therefore maintain sufficient integrity during the required retention period while undergoing predictable degradation or erosion at a rate compatible with therapeutic release. Understanding these coupled processes is essential for designing dynamic hydrogels that transition from stable drug depots to safely cleared materials without uncontrolled burst release. A quantitative interpretation of dynamic hydrogel transport therefore requires consideration of the relative timescales of molecular bond exchange, network relaxation, and therapeutic cargo transport. When bond exchange and network relaxation occur on timescales comparable to cargo diffusion, transient network rearrangement can substantially influence molecular mobility, whereas much slower network dynamics cause the hydrogel to behave more like a quasi-static transport barrier. Representative quantitative benchmarks linking interaction dynamics to network behavior and practical drug-delivery performance are summarized in Table 2. The multiscale relationships linking dynamic crosslink chemistry, kinetic behavior, network properties, molecular transport, and therapeutic applications are summarized schematically in Figure 4.

## 4. Dynamic Networks Governing Drug Loading and Transport

Drug transport in dynamic hydrogels is governed by steric confinement, reversible cargo–network interactions, and time-dependent network rearrangement. Because reversible crosslinks continuously dissociate and reform, mesh accessibility and cargo mobility can evolve on timescales comparable to diffusion and release [7,17,18,19,24,25,27]. The relative timescales of bond exchange, network relaxation, cargo binding, and molecular diffusion therefore determine loading, retention, and transport. The following subsections examine these mechanisms individually.

### 4.1. Drug Loading Within Dynamic Polymer Networks

Drug loading within dynamic hydrogels is governed by a combination of physical confinement and reversible molecular interactions between therapeutic cargo and the polymer network. In its simplest form, a drug can be incorporated into the precursor solution before gelation and subsequently trapped within the hydrated network as reversible crosslinks form. Unlike permanent networks, however, dynamic hydrogels continuously undergo local bond dissociation and reassociation, so the degree of cargo confinement is not determined by mesh size alone. Network relaxation can transiently increase molecular accessibility, while reassociation of junctions can restore confinement around the encapsulated cargo. Consequently, loading efficiency and subsequent retention depend on the relationship among cargo dimensions, equilibrium mesh size, crosslink density, and the characteristic timescale of network rearrangement.

Physical encapsulation remains one of the most straightforward approaches because it avoids direct chemical modification of the therapeutic molecule and is therefore applicable to a broad range of small molecules, proteins, nanoparticles, and other biologically active cargos. Dynamic gelation can be particularly advantageous when drugs are mixed with polymer precursors under mild aqueous conditions and become confined as the reversible network develops. Electrostatic interactions provide another widely applicable mechanism for increasing drug loading and retention without forming permanent covalent linkages. Charged polymer chains can reversibly associate with oppositely charged small molecules, peptides, proteins, and nucleic acids, thereby decreasing their tendency to diffuse freely through the aqueous phase. The effectiveness of this mechanism depends on charge density, spatial distribution of charged groups, solution pH, and ionic strength because counterion screening can substantially weaken electrostatic association under physiological conditions. For protein therapeutics in particular, excessively strong nonspecific adsorption may also perturb protein conformation or reduce biological activity. Miller et al. addressed this problem using a negatively charged self-assembled peptide hydrogel together with engineered cationic guanidinium-containing interaction domains attached to protein cargos. Rather than relying on nonspecific adsorption of the globular protein to the hydrogel, the designed interaction domains selectively associated with the negatively charged fibrillar network. Variation in interaction-domain composition altered the strength of protein–network association and consequently controlled release over extended periods. This approach illustrates how electrostatic loading can be converted from nonspecific sequestration into a tunable affinity mechanism.

Hydrophobic interactions are particularly valuable because the high water content of conventional hydrogels often limits efficient incorporation of poorly water-soluble therapeutics. Dynamic networks containing hydrophobic polymer segments, nanoparticle surfaces, or supramolecular cavities can generate local environments into which such molecules preferentially partition. Because these domains are held together by reversible interactions, they can retain hydrophobic cargo while still allowing exchange with the surrounding aqueous environment. Host–guest hydrogels based on cyclodextrin provide a representative example. Feng et al. developed injectable gelatin hydrogels containing highly dynamic host–guest crosslinks involving β-cyclodextrin moieties. Unoccupied β-cyclodextrin cavities within the network served as binding sites for the hydrophobic small molecule icaritin, enabling its incorporation into the hydrated hydrogel and sustained release for up to approximately two weeks. Thus, the same supramolecular motif can contribute to network formation while providing additional affinity sites for therapeutic loading.

More generally, affinity-based loading provides a means of partially decoupling cargo retention from steric confinement. While physical entrapment becomes progressively ineffective when the mesh is large relative to the therapeutic molecule, affinity interactions can retain molecules that would otherwise diffuse rapidly from the network. Host–guest recognition, peptide–protein binding, electrostatic complexation, hydrogen bonding, and coordination interactions can all serve as transient anchors. The resulting loading capacity is determined by both the number of accessible binding sites and the equilibrium association between cargo and network. In dynamic hydrogels, however, these parameters may be coupled to the crosslinking equilibrium itself. When the same or chemically related functional groups participate in both network formation and cargo binding, drug molecules may compete with polymer chains for available sites. Increasing affinity can therefore improve cargo retention but may simultaneously alter crosslink density, network relaxation, swelling, or mechanical stability. This coupling distinguishes dynamic affinity matrices from conventional hydrogels containing independently immobilized binding ligands.

Reversible drug–polymer interactions can extend this concept further by allowing therapeutic molecules to participate directly in coordination or dynamic bonding equilibria. Such interactions behave as transient molecular tethers: the drug remains associated with the matrix while the bond is formed and becomes mobile following dissociation. Zhao et al. exploited this mechanism in an injectable hydrogel constructed from oxidized dextran and phenylboronic-acid-functionalized poly(ethylene imine), in which doxycycline and metformin were efficiently loaded through reversible B–N coordination [98]. The coordination interactions increased drug loading while remaining susceptible to disruption within a reactive-oxygen-species-rich environment, thereby coupling cargo association with subsequent stimulus-dependent release. This strategy demonstrates an important feature of dynamic drug loading: the interaction responsible for high initial retention can also function as the molecular switch governing later therapeutic mobilization.

The strength and lifetime of drug–polymer association must therefore be considered together rather than maximizing affinity alone. Weak interactions facilitate rapid equilibration and efficient exchange but may provide insufficient retention, particularly for small molecules in highly swollen networks. Conversely, long-lived or multivalent interactions can suppress burst release and increase local residence but may limit the fraction of drug that becomes therapeutically available. An optimal dynamic network should therefore establish a binding regime in which cargo association is sufficiently favorable for loading and storage while dissociation remains accessible on the desired therapeutic timescale. Polymer concentration, binding-site density, crosslink topology, local hydrophobicity, electrostatic environment, and reversible-bond kinetics collectively provide variables for tuning this balance.

Overall, drug loading in dynamic polymer networks should be viewed as an equilibrium between physical confinement, molecular partitioning, reversible binding, and network rearrangement rather than as simple entrapment within a fixed porous structure. Physical encapsulation provides broad applicability, whereas electrostatic, hydrophobic, affinity-based, and reversible coordination interactions introduce progressively greater control over cargo–matrix association. Importantly, these loading mechanisms are inherently linked to subsequent transport: interactions that enhance initial loading also influence the frequency with which a drug exists in a mobile state and therefore determine its effective diffusivity and release behavior. Understanding this coupling between reversible cargo association and network dynamics provides the basis for analyzing molecular transport through dynamic hydrogels in the following sections.

### 4.2. Diffusion Through Dynamic Hydrogel Networks

Following drug incorporation, molecular diffusion through the hydrated polymer network becomes a primary determinant of cargo mobility and subsequent release. For a relatively stable hydrogel in which swelling, degradation, and specific drug–polymer interactions are negligible, transport can often be approximated by Fickian diffusion, where molecular flux is driven by the concentration gradient between the drug-loaded matrix and the surrounding medium. Under these conditions, the hydrogel primarily acts as a physical barrier that reduces the effective diffusion coefficient relative to that in free solution. Experimental studies using crosslinked PEG hydrogels have shown that bulk release at early times can be described using Fickian diffusion models and that the resulting effective diffusivity depends on both hydrogel structure and solute characteristics [99]. Dynamic hydrogels retain this concentration-driven contribution, but the assumption of a constant transport environment becomes less appropriate because reversible crosslinks continuously reorganize the pathways available for molecular motion.

A central structural parameter governing this process is the relationship between hydrogel mesh size and the hydrodynamic dimensions of the diffusing molecule. When a solute is substantially smaller than the characteristic mesh size, it can move predominantly through the water-filled regions of the network with relatively limited obstruction. As the solute dimensions approach the mesh size, however, encounters with polymer chains become increasingly frequent and the available pathways become more tortuous, reducing effective diffusivity. Branco et al. demonstrated this relationship using self-assembled β-hairpin peptide hydrogels and FITC–dextran probes with different hydrodynamic diameters [100]. Increasing peptide concentration increased network crosslinking and decreased mesh size, thereby restricting macromolecular mobility. Importantly, altering the peptide sequence also changed network structure and enabled modulation of diffusion and release over timescales ranging from hours to more than a month. These observations illustrate that mesh-dependent transport is determined by the relative dimensions of the cargo and network rather than by either parameter independently.

Steric hindrance becomes particularly important when molecular dimensions approach or exceed the characteristic network openings. Polymer chains then act as physical obstacles that reduce the number of accessible transport pathways and increase the distance that molecules must traverse to move through the network. Zustiak et al. directly compared proteins of different sizes in PEG hydrogels and observed progressively lower diffusivity with increasing protein dimensions; smaller proteins such as lysozyme moved more rapidly than larger species such as γ-globulin [99]. Similar behavior was observed by Liew et al. in PEGDA-based nanogel networks, where decreasing mesh size increased confinement of encapsulated dextran and markedly reduced the mobile fraction of the macromolecular cargo [101]. Thus, when the mesh dimension becomes comparable to molecular size, relatively modest changes in crosslinking can produce substantial changes in cargo mobility.

The molecular-size effect becomes even more pronounced for macromolecular therapeutics such as proteins, nucleic acids, polysaccharides, and nanoparticle-associated cargos. Small-molecule drugs are frequently much smaller than the aqueous meshes of highly swollen hydrogels and may therefore undergo rapid diffusion unless additional affinity interactions are introduced, as discussed in Section 4.1. In contrast, macromolecules can occupy dimensions comparable to the network mesh and consequently experience substantial size exclusion. Branco et al. studied dextran probes with hydrodynamic diameters of approximately 6–12 nm in peptide hydrogels whose estimated mesh dimensions varied with peptide concentration and sequence, showing that molecular mobility became increasingly sensitive to network structure as the probe dimensions approached those of the mesh [100]. Molecular weight alone, however, is not sufficient to predict diffusion because the relevant parameter is the hydrated molecular dimension, which is additionally influenced by conformation, shape, aggregation state, and interactions with the surrounding polymer chains.

Dynamic networks introduce an additional degree of complexity because the effective steric environment is not permanently fixed. Braegelman et al. directly investigated macromolecular transport through supramolecular hydrogels constructed from cucurbit[7]uril-based host–guest crosslinks with identical network topology but markedly different crosslink dynamics [102]. FRAP measurements using FITC–dextran probes ranging from 20 to 250 kDa revealed strong size-dependent differences in molecular mobility. For smaller probes whose hydrodynamic dimensions were below or near the estimated network mesh size, diffusion proceeded more readily, whereas larger probes approaching or exceeding the mesh dimensions experienced substantial restriction. Notably, transport behavior could not be explained solely by equilibrium mesh size because differences in host–guest crosslink dynamics altered the local environment experienced by the diffusing macromolecules. This finding demonstrates an important distinction between permanent and dynamic hydrogels: molecular accessibility depends not only on the average dimensions of the network but also on how rapidly those dimensions can fluctuate or reorganize.

For molecules that are nominally larger than the equilibrium mesh size, reversible bond exchange may permit transport through transient network openings. Dissociation of a crosslink temporarily reduces local connectivity and allows adjacent polymer chains to rearrange; subsequent reassociation restores network integrity after the solute has moved. Transport under these conditions can therefore be viewed as a coupled process involving molecular diffusion and network rearrangement rather than passive migration through fixed pores. The probability of such movement depends on the relative timescales of solute displacement and junction exchange. If crosslinks reform much faster than a large molecule can traverse the transient opening, steric confinement remains strong. Conversely, sufficiently rapid or persistent network relaxation can increase molecular accessibility even when the equilibrium mesh appears too restrictive for conventional diffusion.

This principle is particularly evident in responsive dynamic networks in which bond exchange can be externally regulated. Gosecka et al. constructed hydrogels based on reversible boronic ester crosslinks and demonstrated that changes in network dynamics produced measurable changes in the diffusion of encapsulated niacin [103]. Upon heating from 25 to 41 °C, the diffusion coefficient of niacin in a hydrogel formed with the shorter crosslinking polymer increased, whereas a network stabilized by greater polymer entanglement exhibited a considerably smaller change. Incorporation of graphene oxide further enabled near-infrared-induced local heating, providing external control over network diffusivity. These results illustrate that dynamic crosslinks can transform diffusivity from a largely fixed material parameter into an actively tunable property.

The relative contributions of mesh restriction and network dynamics nevertheless depend strongly on cargo size. Braegelman et al. found that 20 and 70 kDa dextran probes, whose dimensions were below or close to the estimated mesh size, displayed diffusion trends consistent with differences in network dynamics, whereas 150 and 250 kDa probes exceeding the mesh dimension exhibited much stronger confinement and more complex transport behavior [102]. Therefore, increasing bond-exchange kinetics does not necessarily eliminate steric restrictions for very large molecules. Rather, dynamic relaxation modifies the energetic and structural barriers encountered by the solute, while the underlying size ratio between the cargo and network remains a fundamental constraint.

Another important consequence is that diffusion in dynamic hydrogels may depart from ideal Fickian behavior. Fickian models assume that the effective diffusion coefficient and transport geometry remain approximately constant over the timescale of measurement. In reversible networks, however, temporal changes in mesh accessibility, local crosslink density, swelling, and cargo–network association can cause the effective diffusivity itself to evolve. Apparent non-Fickian transport may therefore emerge when the characteristic timescale of network relaxation becomes comparable to that of molecular diffusion. This does not mean that concentration gradients cease to drive transport; instead, molecular diffusion becomes coupled to a time-dependent polymer environment. Distinguishing these contributions is essential when interpreting release profiles from dynamic hydrogels because an observed change in release rate may originate from altered steric accessibility rather than from changes in drug concentration alone.

Overall, diffusion through dynamic hydrogel networks is controlled by the interplay between concentration-driven molecular motion, mesh size, steric hindrance, molecular dimensions, and network rearrangement. When the cargo is much smaller than the mesh, transport may approach conventional Fickian diffusion, whereas molecules approaching the mesh dimension become increasingly sensitive to physical obstruction. For larger macromolecules, transient opening of reversible junctions can provide additional transport pathways, but the efficiency of this process depends on the kinetics of bond exchange and polymer relaxation. Thus, the mesh size of a dynamic hydrogel should be regarded not as a rigid molecular sieve but as a time-dependent transport constraint whose accessibility evolves with network dynamics. This coupling between solute dimensions and reversible network motion provides an important basis for controlling therapeutic mobility and ultimately the rate and duration of drug release.

### 4.3. Polymer–Cargo Interactions

The diffusion behavior described in Section 4.2 can be substantially modified when therapeutic molecules interact reversibly with the polymer network. In the absence of appreciable affinity, cargo mobility is governed primarily by concentration gradients and steric restrictions imposed by the hydrogel mesh. When reversible polymer–cargo interactions are present, however, molecules repeatedly alternate between relatively mobile and network-associated states. The resulting effective transport rate therefore depends not only on molecular size and mesh accessibility but also on the strength, lifetime, and spatial density of these transient interactions. Electrostatic attraction, hydrogen bonding, hydrophobic association, and host–guest recognition are particularly relevant because they are sufficiently reversible to retard molecular transport without permanently immobilizing the therapeutic cargo. Their dynamic nature also allows changes in pH, ionic strength, temperature, or competing molecular species to alter cargo retention over time.

Electrostatic interactions provide one of the most direct mechanisms for regulating cargo mobility because many therapeutic molecules and biological macromolecules carry ionizable groups under physiological conditions. Oppositely charged polymer chains and cargo molecules can associate reversibly, thereby decreasing the fraction of freely diffusing solute and slowing its escape from the hydrogel. The magnitude of this effect depends on polymer charge density, cargo valency, pH, and ionic strength. Elsawy et al. demonstrated this relationship using β-sheet-forming peptide hydrogels with systematically varied charge distributions and doxorubicin as a model drug [50]. In negatively charged FE peptide hydrogels, substantial doxorubicin retention was attributed to attraction between the anionic peptide fibers and positively charged drug molecules, and the amount retained in mixed F8/FE networks increased with the fraction of negatively charged fibers. This observation shows that cargo transport can be regulated by modifying the chemical identity of the polymer network without necessarily changing the overall dimensions of the hydrogel mesh.

Electrostatic affinity is particularly useful for macromolecular therapeutics because multiple charged residues can engage the network simultaneously. Such multivalent interactions may produce substantially longer residence times than would be expected from individual ionic contacts. As described in Section 4.1, Miller et al. used cationic guanidinium-containing interaction domains to reversibly associate protein therapeutics with negatively charged peptide nanofibers. By controlling the composition of these domains, the strength of protein–hydrogel association and subsequent release could be adjusted. This strategy also illustrates an important limitation of electrostatic retention: physiological salts screen ionic interactions, while changes in protonation state can alter both cargo and polymer charge. Electrostatic control is therefore inherently coupled to the local chemical environment and can provide either sustained retention or pH-/ionic-strength-dependent mobilization.

Hydrogen bonding offers a second mechanism for transient cargo association. Hydroxyl, carboxyl, amide, amine, carbonyl, and ether groups commonly present in natural and synthetic hydrogel polymers can function as hydrogen-bond donors or acceptors, while many drugs contain complementary functionalities. Individual hydrogen bonds are relatively weak and continuously exchange in aqueous environments, but multiple cooperative contacts can significantly increase cargo residence within the polymer matrix. The resulting interaction is especially sensitive to molecular orientation and local functional-group density because effective association requires appropriate spatial alignment of donor and acceptor groups. Hydrogen bonding is also strongly influenced by competition with surrounding water molecules, which distinguishes hydrogel environments from less hydrated polymer matrices.

The contribution of hydrogen bonding can vary with cargo concentration and local network organization. Huang et al. investigated molecular interactions of gemcitabine and doxorubicin with a self-healing glycol-chitosan/telechelic PEG hydrogel using full-atomistic simulations [104]. For doxorubicin at relatively low loading, hydrogen bonding represented a dominant component of hydrogel–drug association, with ether-containing PEG segments participating frequently in interactions with the drug. At higher doxorubicin concentrations, however, van der Waals interactions became increasingly important. Gemcitabine exhibited a different interaction profile, with van der Waals adsorption contributing strongly to its association with the same network. These results demonstrate that polymer–cargo interactions cannot always be assigned solely from polymer chemistry: the same dynamic hydrogel may interact differently with different therapeutic molecules, and the predominant interaction may change as loading concentration increases.

Hydrophobic interactions become particularly important for poorly water-soluble drugs that have limited thermodynamic compatibility with the highly hydrated phase of conventional hydrogels. Hydrophobic polymer segments, aromatic peptide residues, amphiphilic assemblies, or nanoparticle surfaces can create relatively nonpolar microdomains that favor partitioning of hydrophobic cargo away from bulk water. Unlike covalent attachment, such association remains reversible because cargo molecules can continuously exchange between hydrophobic domains and the surrounding aqueous phase. Consequently, hydrophobic interactions can simultaneously increase apparent solubility within the hydrogel and decrease the rate at which the drug escapes from the matrix.

Hydrophobic association can therefore produce heterogeneous transport environments even when the hydrogel appears macroscopically homogeneous. A therapeutic molecule may preferentially partition into transient hydrophobic domains and subsequently diffuse only after returning to the aqueous phase. In multicomponent dynamic hydrogels, these local environments may originate from the same associations responsible for network formation. Polymer–nanoparticle hydrogels, for example, can contain hydrophobic nanoparticle-associated domains together with a hydrated continuous phase, enabling cargos with substantially different physicochemical properties to coexist within a single material [18]. From a transport perspective, such systems behave less like uniform porous matrices and more like dynamically exchanging multiphase reservoirs in which partitioning between local domains influences apparent diffusivity.

Host–guest interactions provide a more structurally defined form of reversible affinity. Host molecules such as cyclodextrins contain cavities capable of selectively accommodating compatible guest groups through a combination of hydrophobic inclusion, van der Waals interactions, and molecular recognition. Because host–guest complexes continuously associate and dissociate, they can serve both as reversible network junctions and as transient binding sites for therapeutic molecules. The affinity can be tuned through host and guest chemistry, and the presence of multiple guest groups on a single cargo can introduce multivalent binding that substantially increases retention.

In the dynamic gelatin hydrogel reported by Feng et al., β-cyclodextrin-based host–guest interactions contributed to reversible network formation while unoccupied cyclodextrin cavities also provided sites for incorporation of the hydrophobic drug icaritin. The resulting affinity-mediated retention supported prolonged local delivery rather than rapid diffusion through the highly hydrated matrix. A more explicit demonstration of tunable host–guest-mediated protein transport was provided by D’Elia et al., who developed granular hydrogels containing β-cyclodextrin host groups and proteins modified with adamantane guests [105]. As shown in Figure 5, the platform was constructed from methacrylated cyclodextrin (MeCD) and methacrylated dextran (DexMA), which were photopolymerized to generate cyclodextrin-containing hydrogel networks that subsequently served as affinity-rich building blocks for avidity-controlled biotherapeutic delivery.

Increasing the number of adamantane groups attached to a protein increased binding avidity and prolonged the lifetime of the host–guest complex, producing markedly reduced protein release over prolonged incubation. Thus, cargo residence can be tuned not only through the intrinsic association constant of an individual host–guest pair but also through the valency with which those interactions are presented.

An important feature shared by all of these interactions is that their influence on transport is kinetic as well as thermodynamic. A high equilibrium affinity generally favors cargo retention, but release also depends on how rapidly individual associations dissociate and reform. A molecule interacting weakly but frequently with numerous sites may exhibit substantially different transport from one forming fewer but longer-lived complexes. Moreover, when polymer chains themselves participate in dynamic crosslinking, cargo binding and network dynamics can become coupled. Occupation of polymer functional groups by a drug may reduce their availability for crosslink formation, whereas network rearrangement may expose previously inaccessible binding sites. The effective transport environment can therefore evolve as both the polymer network and the cargo-binding equilibrium reorganize.

Multiple interaction modes may also act simultaneously. Charged aromatic drugs, proteins with heterogeneous surfaces, and amphiphilic therapeutic molecules can engage electrostatic, hydrogen-bonding, hydrophobic, and aromatic interactions at the same time. The dominant mechanism may consequently shift with environmental conditions. Changes in pH can alter ionization and hydrogen bonding; ionic strength can screen electrostatic attraction; temperature can change hydrophobic association; and competitive guest molecules can displace host-bound cargo. Such coupling provides dynamic hydrogels with considerable design flexibility but also complicates prediction of release behavior from a single material parameter. Polymer–cargo affinity should therefore be characterized together with mesh size and network relaxation rather than treated as an independent property.

Overall, polymer–cargo interactions transform dynamic hydrogels from passive diffusion barriers into reversible affinity landscapes in which therapeutic molecules continuously exchange between mobile and network-associated states. Electrostatic interactions provide environmentally sensitive charge-based retention, hydrogen bonding enables cooperative yet rapidly exchangeable association, hydrophobic interactions promote partitioning of poorly water-soluble cargos into nonpolar domains, and host–guest recognition provides comparatively selective and tunable molecular binding. The balance among these interactions determines the fraction of cargo available for diffusion and therefore directly couples molecular affinity to effective transport and release kinetics. This framework provides the mechanistic basis for understanding affinity-controlled and dynamically regulated drug release from hydrogel networks.

### 4.4. Network Relaxation and Molecular Transport

Molecular transport in dynamic hydrogels is inherently coupled to the timescale over which reversible crosslinks exchange and the polymer network relaxes. In permanently crosslinked hydrogels, the average network topology remains largely fixed during diffusion, and molecular mobility can therefore be interpreted primarily in terms of mesh size, steric obstruction, and polymer–cargo interactions. Dynamic networks differ because individual junctions continuously dissociate and reform, allowing polymer chains to rearrange without complete loss of macroscopic network integrity. As a result, the transport pathways experienced by a therapeutic molecule evolve over time, and the effective diffusivity may depend on the relative timescales of molecular motion and network relaxation.

Bond exchange kinetics provide the molecular origin of this behavior. The rate of crosslink dissociation determines how frequently a local junction becomes temporarily unconstrained, whereas reassociation determines how rapidly network connectivity is restored. When bond exchange is slow relative to solute motion, the hydrogel behaves approximately as a quasi-static network and cargo encounters a comparatively persistent steric environment. As exchange becomes faster, transient rearrangements occur more frequently and can increase the configurational freedom of polymer chains. Braegelman et al. demonstrated this relationship using cucurbit[7]uril-based supramolecular hydrogels with similar macromolecular topology but substantially different host–guest exchange dynamics [102]. Stress-relaxation measurements showed relaxation on the scale of seconds for the more dynamic xylitylamine-based network, whereas the higher-affinity adamantanol-based network relaxed over much longer timescales. These differences originated from distinct energetic barriers for crosslink dissociation and were subsequently reflected in macromolecular transport behavior.

Stress relaxation therefore provides a useful macroscopic measure of the network rearrangements generated by molecular-scale bond exchange. Following deformation, a dynamic hydrogel can progressively dissipate stress as reversible junctions break, polymer chains reorganize, and new junctions form in configurations that carry less of the original load. The characteristic relaxation time consequently provides an experimentally accessible link between crosslink kinetics and polymer mobility. Although stress relaxation is measured mechanically, the same underlying rearrangements are relevant to molecular transport because relaxation represents the ability of the network to explore new configurations. Faster relaxation generally produces more frequent structural fluctuations, whereas slowly relaxing networks retain their local architecture for longer periods.

This coupling becomes particularly important when the size of a transported molecule approaches the equilibrium mesh size. Small molecules that can readily move through water-filled network spaces may remain relatively insensitive to individual crosslink rearrangements. Larger cargos, however, may require local polymer displacement or junction exchange before sufficient free volume becomes available for passage. Dynamic mesh rearrangement can therefore generate transient transport pathways that do not exist in the time-averaged network structure. In the supramolecular systems investigated by Braegelman et al., macromolecules with dimensions near the estimated mesh size displayed transport behavior that depended on crosslink dynamics, with the more rapidly exchanging network supporting faster diffusion for the smaller dextran probes. For solutes exceeding the mesh size, the relationship became more complex because network exclusion and structural heterogeneity introduced additional transport pathways [102]. These observations emphasize that relaxation does not simply “increase pore size”; instead, it changes how frequently and for how long molecularly accessible configurations are generated.

The characteristic timescale of cargo transport should therefore be considered relative to the relaxation time of the network. When molecular diffusion occurs much faster than network relaxation, the cargo effectively experiences a nearly frozen polymer environment during each displacement. In contrast, when the two timescales become comparable, molecular movement and network reorganization become coupled. Under this regime, classical descriptions based on a single static mesh size become increasingly incomplete because the molecule samples a continuously evolving distribution of local environments. If network relaxation is considerably faster than the characteristic transport process, the cargo instead experiences an averaged, rapidly fluctuating environment in which transient junction opening may reduce long-lived steric confinement.

An important consequence of this behavior is that diffusivity in dynamic hydrogels can become time- and condition-dependent rather than representing a single intrinsic constant. Changes in temperature, pH, competitive binding, or other environmental parameters can alter bond-exchange kinetics and thereby modify the rate of network rearrangement. Gosecka et al. demonstrated this principle in boronic ester-crosslinked hydrogels, where increasing temperature accelerated molecular transport and increased the measured diffusion coefficient of encapsulated niacin [103]. Incorporation of graphene oxide further enabled near-infrared-induced heating and modulation of diffusivity, illustrating how an external stimulus can regulate molecular transport indirectly by altering the dynamic state of the network. Thus, the effective diffusivity of a therapeutic cargo can evolve even without a permanent change in polymer composition.

From a drug-delivery perspective, relaxation kinetics must therefore be optimized rather than simply accelerated. Very rapid bond exchange can facilitate molecular transport and reduce steric confinement but may also increase erosion or premature cargo loss. Conversely, slowly exchanging networks can provide prolonged retention but may restrict the transport of large therapeutic molecules or limit responsiveness to physiological triggers. The appropriate relaxation timescale consequently depends on cargo dimensions, desired release duration, polymer–cargo affinity, and the stability required at the administration site. Designing dynamic hydrogels for controlled transport requires matching these molecular and network timescales so that structural rearrangement occurs rapidly enough to permit therapeutic mobility while remaining sufficiently slow to preserve depot integrity.

Overall, network relaxation provides the temporal dimension that distinguishes molecular transport in dynamic hydrogels from diffusion through static polymer matrices. Bond exchange kinetics determine the rate of local junction rearrangement, stress relaxation reflects the resulting macroscopic network mobility, and dynamic mesh fluctuations regulate the transient accessibility of transport pathways. Consequently, diffusivity should be regarded as an effective, potentially time-dependent property arising from the interplay between cargo motion and network reorganization. Controlling this relationship provides a powerful route for programming sustained, adaptive, and stimulus-dependent molecular transport in dynamic hydrogel drug-delivery systems.

### 4.5. Degradation- and Erosion-Controlled Transport

The preceding sections considered molecular transport through intact or dynamically rearranging networks; therapeutic release can also become directly coupled to progressive loss of hydrogel structure. Cleavage of polymer backbones or crosslinks decreases network connectivity, while subsequent dissolution of disconnected polymer chains produces erosion and loss of hydrogel mass. These processes progressively reduce steric confinement and can eventually eliminate the physical barrier retaining therapeutic cargo. Degradation-controlled transport is therefore particularly important for macromolecules, proteins, nanoparticles, and strongly retained drugs that cannot readily diffuse through the original network. In dynamic hydrogels, this mechanism becomes more complex because reversible junction dissociation may increase molecular accessibility before irreversible degradation or macroscopic erosion occurs [23].

At the early stages of degradation, cleavage of only a fraction of network junctions can substantially alter transport without producing measurable mass loss. Reduced crosslink density increases polymer-chain mobility and enlarges the distribution of accessible transport pathways, allowing previously confined molecules to diffuse more readily. As degradation progresses toward the gel–sol transition, sufficiently large portions of the network lose connectivity and become soluble, shifting cargo release from diffusion through a weakened matrix toward erosion-associated transport. Wu and Schultz directly quantified this relationship in enzymatically degradable polymer hydrogels and demonstrated that molecular release was closely correlated with bulk degradation of the network [96]. Importantly, cargo size influenced the relationship between degradation and release, indicating that degradation does not simply liberate all encapsulated species at the same rate but progressively modifies size-dependent transport constraints.

The kinetics of bond cleavage are therefore as important as the initial network structure. Carberry et al. investigated thioester-crosslinked hydrogels and showed that degradation kinetics could be systematically regulated through thiol concentration, thiol pK_a, pH, and molecular presentation [107]. These parameters controlled the rate of thiol–thioester exchange and consequently the timescale over which network connectivity was lost. From a drug-delivery perspective, such chemistry provides a direct link between molecular reaction kinetics and the evolution of transport pathways: slowly cleaving junctions preserve steric confinement for extended periods, whereas faster exchange progressively increases network mobility and ultimately promotes dissolution. Thus, degradation rate can function as a programmable transport parameter rather than merely determining the final lifetime of the hydrogel.

Dynamic crosslinks introduce an additional route to network destabilization because structural loss does not always require irreversible polymer-backbone cleavage. Changes in pH, redox state, ROS concentration, competitive binding, or other environmental factors can shift reversible crosslink equilibria toward the dissociated state. At moderate levels of junction dissociation, the primary consequence may be enhanced relaxation and transient mesh rearrangement, as discussed in Section 4.4. Once the fraction of associated junctions decreases below that required to maintain network connectivity, however, soluble polymer chains can escape and the material undergoes macroscopic erosion.

The spatial distribution of degradation further determines whether cargo transport is associated predominantly with surface erosion or bulk erosion. Surface erosion occurs when network disruption near the hydrogel–medium interface proceeds more rapidly than penetration of the degrading species into the interior. Under these conditions, the external boundary gradually recedes while the inner network remains comparatively intact, allowing cargo release to track advancement of the erosion front. Tae et al. demonstrated controlled surface erosion in physically associated PEG hydrogels formed through reversible fluoroalkyl interactions [106]. Networks exhibiting sol–gel coexistence underwent relatively slow surface erosion, whereas related polymers exhibiting single-phase behavior dissolved more rapidly through bulk erosion. Importantly, changes in molecular architecture influenced both relaxation and erosion, demonstrating direct coupling between transient junction properties and macroscopic material loss.

Bulk erosion instead occurs when water or reactive species penetrate the hydrogel faster than network cleavage proceeds, allowing degradation throughout the material before extensive surface loss occurs Bulk degradation frequently produces release profiles in which diffusion and network loss operate simultaneously rather than as independent mechanisms. As crosslinks are progressively removed throughout the hydrogel, swelling and molecular accessibility increase before complete erosion occurs. Wu and Schultz showed that release from enzymatically degradable hydrogels was coupled to the evolving degradation state of the matrix and that the relationship depended on molecular size [96]. Consequently, a therapeutic molecule may initially experience strong steric confinement, subsequently diffuse more rapidly as network connectivity decreases, and finally be released together with soluble polymer fragments. An experimentally observed acceleration in release therefore does not necessarily indicate a change in drug–polymer affinity; it may instead reflect the evolving architecture of a degrading network.

This principle is particularly relevant for biologic therapeutics whose dimensions make diffusion through intact networks intrinsically slow. Gregoritza et al. developed thermoresponsive hydrogels crosslinked through Diels–Alder chemistry for controlled antibody delivery [108]. The system combined initial physical gelation with subsequent covalent network formation and gradual degradation, enabling release of antibody cargo over extended periods. Such approaches illustrate how degradation can be deliberately incorporated as a transport mechanism when long-term retention is required initially but complete liberation of a large therapeutic molecule is ultimately necessary.

The relative rates of molecular diffusion, crosslink cleavage, penetrant transport, and polymer dissolution therefore determine which transport mechanism dominates at a given time. When cargo diffusion is rapid compared with degradation, release remains predominantly diffusion controlled. When molecular motion is strongly restricted but degradation progressively increases network accessibility, release becomes degradation assisted. If polymer dissolution occurs on a timescale comparable to or faster than cargo diffusion, erosion can become the dominant release mechanism. Importantly, these regimes can occur sequentially within the same hydrogel rather than representing mutually exclusive material classifications.

Overall, degradation- and erosion-controlled transport represents the transition from molecular-scale network rearrangement to progressive macroscopic loss of the drug-delivery matrix. Partial crosslink cleavage increases molecular accessibility, continued network dissociation reduces steric confinement, and eventual surface or bulk erosion removes the matrix responsible for cargo retention. Dynamic hydrogel design should therefore coordinate bond-exchange and degradation kinetics with cargo dimensions and the desired therapeutic window. Controlling these coupled timescales enables materials that remain sufficiently stable during administration and early treatment while subsequently increasing permeability or undergoing programmed erosion to achieve sustained, complete, or disease-responsive therapeutic release. Representative studies linking dynamic network characteristics to therapeutic transport and delivery are summarized in Table 3.

**Table 3:** Representative studies linking dynamic network characteristics to therapeutic transport and delivery.

Dynamic Interaction/System	Kinetic or Dynamic Feature	Resulting Network Property	Transport/Release Mechanism	Cargo/Application	Ref.
Imine-crosslinked chitosan/PEG dynamer	Reversible imine formation; crosslinking-dependent retention	Tunable network confinement	Physical entrapment combined with polymer–cargo interactions	5-FU	[109]
Polymer–nanoparticle multivalent interactions	Rapid reversible adsorption and reassociation	Shear-thinning, self-recovery, heterogeneous cargo domains	Partitioning-mediated loading and release	Hydrophilic and hydrophobic therapeutics	[18]
CB[7]-based host–guest network	Crosslink exchange ranging from rapid to more persistent relaxation	Time-dependent mesh accessibility and relaxation	Size- and dynamics-dependent diffusion	FITC–dextran (20–250 kDa)	[104]
Supramolecular peptide assembly	Reversible hydrophobic and noncovalent association	Hydrophobic affinity domains	Affinity-controlled diffusion and retention	Curcumin and antitumor agents	[110]
PEG microhydrogel	Crosslinking-dependent erosion over hours to ~10 days	Tunable network erosion	Erosion-controlled release	Fenofibrate	[111]
Boronic ester-crosslinked PEG	Environment-sensitive crosslink equilibrium	Relaxation and surface erosion	Mass-loss-coupled release	β-Galactosidase	[23]
Schiff-base/boronate ester network	pH- and ROS-responsive crosslink destabilization	Stimulus-induced network disintegration	Degradation-assisted responsive release	Bevacizumab/osteoarthritis	[92]
Disulfide-crosslinked hyaluronic acid	Enzymatic or reductive cleavage	Surface- or bulk-dominated erosion	Degradation-coupled protein release	SDF-1α	[112]

### 4.6. Mathematical Models of Drug-Release Kinetics

Mathematical modeling provides a useful means of distinguishing diffusion-, concentration-, relaxation-, and degradation-dominated release behavior. However, because dynamic hydrogels undergo time-dependent changes in crosslink density, mesh accessibility, and network integrity, conventional release models should be interpreted as mechanistic approximations rather than universally applicable descriptions. Representative models and their relevance to dynamic hydrogel systems are summarized in Table 4.

For dynamic hydrogels, model selection should therefore be guided by the dominant transport mechanism and by whether network relaxation, bond exchange, swelling, or degradation changes significantly during the release period.

## 5. Controlled and Stimuli-Responsive Therapeutic Release

Dynamic hydrogels regulate therapeutic release through coupled diffusion, reversible cargo binding, bond exchange, and degradation [23,108]. Endogenous cues such as pH, redox conditions, ROS, and enzymes, as well as external stimuli including temperature, light, ultrasound, magnetic fields, and electrical signals, can alter network permeability or stability [92]. These mechanisms enable sustained, triggered, sequential, and on-demand release. The following sections compare the major release strategies.

### 5.1. Sustained and Long-Term Drug Release

Sustained therapeutic release from dynamic hydrogels arises when the timescale of cargo escape is extended beyond that of free diffusion in solution while the hydrogel remains sufficiently stable to function as a local depot. Importantly, sustained release is not a single transport mechanism but an emergent consequence of several coupled processes. Molecular diffusion may be slowed by mesh restriction, reversible polymer–cargo interactions may transiently immobilize the therapeutic agent, and progressive degradation may gradually remove the barriers responsible for confinement. In dynamic networks, these mechanisms are further influenced by continuous crosslink exchange and relaxation. The duration of delivery therefore reflects the relative timescales of cargo diffusion, reversible binding, network rearrangement, and matrix degradation rather than any single material parameter.

Diffusion-controlled release provides the simplest route to prolonged delivery when therapeutic molecules are physically entrapped but do not strongly interact with the network. Increasing polymer concentration or reducing effective mesh accessibility increases the path length and steric resistance experienced by the cargo, thereby lowering its effective diffusion coefficient. Dynamic polymer–nanoparticle (PNP) hydrogels provide an illustrative example because transient multivalent interactions between hydrophobically modified polymers and nanoparticles generate a cohesive but rearrangeable network [18]. Gale et al. used this architecture to deliver SARS-CoV-2 receptor-binding-domain (RBD) antigen together with vaccine adjuvants [113]. The PNP hydrogel retained both RBD and CpG and markedly prolonged local antigen availability compared with bolus administration; fluorescently labeled RBD remained detectable at the injection site for approximately three weeks. Thus, reversible network formation can preserve injectability while creating a sufficiently persistent diffusional barrier for prolonged local delivery.

Hierarchical carrier–hydrogel systems can extend diffusion-controlled release further by introducing multiple sequential transport barriers. Instead of allowing the drug to diffuse directly from the hydrogel, therapeutic molecules can first escape from an embedded nanoparticle or microsphere and subsequently migrate through the surrounding polymer network. Li et al. recently incorporated kartogenin-loaded PLGA/PEG nanoparticles into a bioadhesive chitosan hydrogel crosslinked through dynamic imine bonds [114]. Whereas approximately 94% of kartogenin was released from the nanoparticles alone within three weeks, cumulative release from the nanoparticle-containing dynamic hydrogel was only approximately 70% over the same period. The surrounding hydrogel therefore functioned as an additional retention barrier, producing a dual-release architecture in which nanoparticle release and hydrogel transport acted sequentially.

Affinity-controlled release provides a complementary strategy when diffusion alone is too rapid to achieve therapeutically useful retention. Reversible binding sites within the hydrogel decrease the fraction of cargo existing in a freely mobile state, allowing sustained release even from relatively hydrated networks with large effective meshes. Lee et al. demonstrated this concept using adamantane-functionalized Pluronic F127 and polymerized β-cyclodextrin to form a host–guest-crosslinked injectable hydrogel [115]. The β-cyclodextrin groups contributed not only to network stabilization but also to complexation with protein cargos such as insulin and gelatin, allowing their release rates to be regulated through molecular affinity. The resulting hydrogel remained structurally detectable after subcutaneous administration for up to 30 days, demonstrating how reversible molecular recognition can simultaneously enhance depot stability and prolong therapeutic release.

Degradation-controlled release becomes increasingly important when therapeutic molecules are too large or too strongly retained to diffuse efficiently through an intact network. As crosslinks or polymer chains are progressively cleaved, network connectivity decreases and molecular accessibility increases until cargo can diffuse through the weakened matrix or is liberated during erosion. As discussed in Section 4.5, environmentally regulated boronic ester networks can couple biomolecule release directly to network mass loss [23], while degradable Diels–Alder-crosslinked hydrogels have enabled prolonged antibody delivery [108]. Dynamic covalent networks are particularly attractive because the chemistry governing network lifetime can be adjusted independently, at least in part, from the initial mechanical properties.

Jiang et al. demonstrated this principle using cellulose-based hydrogels containing both β-cyclodextrin–adamantane host–guest interactions and dynamic acylhydrazone bonds [116]. The host–guest interactions provided rapid self-recovery and injectability, whereas the acylhydrazone network exhibited slow hydrolytic degradation lasting more than 30 days. When loaded with doxorubicin, this relatively persistent network provided prolonged local drug delivery while retaining pH-responsive release capability. This design highlights an important feature of long-term dynamic hydrogel systems: fast reversible interactions can provide the short-timescale rearrangement needed for administration and self-healing, while more persistent dynamic bonds determine depot lifetime and long-term drug liberation.

The design of long-term delivery systems consequently requires a balance between therapeutic retention and therapeutic availability. Excessively rapid diffusion produces burst release and premature depletion, whereas overly restrictive meshes or excessively strong affinity may leave a large fraction of cargo permanently trapped. Likewise, rapid degradation can shorten depot lifetime, while excessively persistent matrices may outlast the required treatment period or delay complete drug recovery. The optimal system should therefore coordinate diffusion coefficients, binding-site density and lifetime, crosslink-exchange kinetics, and degradation rate with the pharmacological window of the therapeutic agent.

Overall, sustained release from dynamic hydrogels is achieved through the coordinated regulation of diffusion, reversible affinity, degradation, and local depot persistence. Diffusional barriers provide baseline control of molecular escape, affinity interactions extend retention without requiring extremely dense networks, and programmed degradation permits eventual liberation of strongly confined cargos. When these processes are appropriately balanced, dynamic hydrogels can maintain local therapeutic exposure over days to weeks while preserving injectability, conformability, and responsiveness. Such long-term delivery establishes the baseline upon which the stimulus-responsive mechanisms discussed in the following sections can provide additional spatial and temporal control.

### 5.2. Endogenous Stimuli-Responsive Release

Endogenous stimuli-responsive hydrogels exploit biochemical differences between healthy and diseased tissues to regulate therapeutic release without requiring an externally applied trigger. Local variations in pH, redox potential, reactive oxygen species (ROS), and enzyme activity can alter the equilibrium or stability of responsive crosslinks, producing changes in network relaxation, swelling, permeability, or degradation. Unlike sustained-release systems designed to maintain relatively constant transport over time, endogenous-responsive networks remain comparatively stable under physiological conditions and increase cargo mobility when exposed to a disease-associated microenvironment. This behavior is particularly attractive for localized therapy because the molecular signal responsible for disease progression can simultaneously function as the trigger for therapeutic release.

Among endogenous signals, pH is one of the most widely exploited because substantial spatial variations exist across physiological and pathological environments. Dynamic covalent bonds such as imines, hydrazones, and related Schiff-base linkages are particularly useful because their equilibrium and hydrolytic stability depend strongly on proton concentration. Under near-neutral physiological conditions, these junctions can maintain network integrity and restrict cargo transport, whereas acidic conditions increase bond dissociation and promote network relaxation or degradation. Qu et al. demonstrated this concept using injectable hydrogels formed through dynamic Schiff-base linkages between N-carboxyethyl chitosan and dibenzaldehyde-terminated PEG [117]. Doxorubicin was retained within the self-healing network but exhibited enhanced release under acidic conditions, linking the dynamic crosslink chemistry directly to the acidic tumor environment. This approach allowed the hydrogel to function as a relatively stable local depot under physiological conditions while increasing therapeutic availability under conditions relevant to hepatocellular carcinoma.

The magnitude of pH responsiveness must nevertheless be matched to the biological environment of interest. Tumor extracellular pH is only moderately lower than that of normal tissues, so highly acid-labile networks may respond insufficiently to the relatively narrow physiological difference or become unstable before administration. Conversely, the gastrointestinal tract provides much larger pH transitions that can be exploited for site-selective oral delivery. Knipe et al. developed enzyme- and pH-responsive microencapsulated nanogels for oral siRNA delivery using poly(methacrylic acid-co-N-vinyl-2-pyrrolidone)-based responsive networks [118]. The system remained relatively protected under acidic gastric conditions but became hydrated and accessible at the higher pH encountered in the intestine, while enzymatic degradation of the surrounding structure facilitated release of siRNA-containing nanogels. The released siRNA subsequently mediated TNF-α knockdown, demonstrating how physiological pH gradients and local enzyme activity can be combined to protect sensitive cargos during transit and promote their release at the intended gastrointestinal site.

Redox-responsive release provides a complementary mechanism based on differences in reducing potential between biological compartments and pathological tissues. Disulfide and diselenide bonds are particularly attractive because they can function as network-forming junctions while remaining susceptible to cleavage in reducing environments. Intracellular glutathione concentrations and altered redox conditions in tumors can therefore convert otherwise stable crosslinks into degradable molecular switches. Gulfam et al. developed injectable cartilage-acellularized-matrix hydrogels crosslinked using a water-soluble diselenide-containing linker [119]. Under simulated physiological conditions, DOX leakage remained limited, whereas exposure to reducing conditions produced sustained network-responsive release, with more than 90% of the encapsulated DOX released within 96 h. Because cleavage of diselenide junctions simultaneously reduced network integrity and increased drug accessibility, the system illustrates how redox chemistry can translate a biochemical gradient into a change in macroscopic release behavior.

ROS-responsive systems exploit the opposite side of redox imbalance. Elevated concentrations of hydrogen peroxide and other ROS are associated with tumors, inflammatory disorders, diabetic wounds, and several degenerative diseases. Responsive groups such as phenylboronic esters, thioketals, thioethers, and selenium-containing bonds can undergo oxidation that changes polymer solubility or cleaves network junctions. Zhang et al. incorporated H_2_O_2_-cleavable phenylboronic-acid-derived linkers into four-arm PEG networks to generate oxidation-responsive degradable hydrogels [120]. The networks responded to relatively low H_2_O_2_ concentrations, and incorporation of glucose oxidase enabled glucose to be converted enzymatically into an oxidative H_2_O_2_ signal that subsequently promoted network degradation and insulin release. Interestingly, direct H_2_O_2_ exposure produced bulk degradation, whereas glucose-triggered generation of H_2_O_2_ resulted in a more surface-dominated degradation process. This example demonstrates that endogenous responsiveness depends not only on the chemical identity of the trigger but also on where and how rapidly that trigger is generated and transported through the network.

ROS-responsive degradation can also be directly coupled to local cancer therapy. Zhang et al. developed a poly(deca-4,6-diynedioic acid)/pullulan hydrogel in which ROS progressively degraded the polymer backbone, thereby regulating release of a photosensitizer and an immune-checkpoint-blocking antibody [121]. Increasing ROS accelerated hydrogel degradation, while ROS production during photodynamic therapy further promoted antibody and photosensitizer release. Without enhanced ROS generation, only a small fraction of the model antibody escaped over 15 days, whereas ROS-mediated degradation substantially increased release and shortened hydrogel persistence in vivo. The network therefore acted not merely as an ROS-sensitive carrier but as a dynamic depot whose degradation rate evolved with the local oxidative state, enabling sustained yet condition-dependent postoperative cancer therapy. As illustrated in Figure 6, ROS generated during photodynamic therapy simultaneously promotes degradation of the hydrogel depot and therapeutic release, thereby coupling Ce6-mediated photodynamic activity with sustained local delivery of anti-CD47 antibodies for postsurgical cancer treatment.

The distinction between redox- and ROS-responsive mechanisms is not always absolute. Selenium-containing junctions, for example, can respond to both reducing and oxidizing species, and boronic ester chemistry can be affected by ROS while also participating in pH-dependent dynamic equilibria. The pH/ROS dual-responsive hydrogel described by Qin et al. illustrates this coupling particularly well [92]. Dynamic Schiff-base and boronate-ester bonds maintained injectability and self-healing but became increasingly destabilized under acidic and oxidative conditions associated with the osteoarthritic microenvironment. Combined pH and ROS stimulation accelerated network disintegration and bevacizumab release, demonstrating that simultaneous biochemical abnormalities can provide stronger selectivity than reliance on a single endogenous signal [92].

Enzyme-responsive release offers an additional level of biological specificity because the hydrogel can be designed around peptide or polymer sequences selectively cleaved by enzymes associated with particular disease states. Matrix metalloproteinases (MMPs) are especially useful targets because their expression and activity increase during tissue remodeling, inflammation, tumor invasion, and chronic wound healing. Incorporation of MMP-cleavable peptide sequences as hydrogel crosslinkers converts enzymatic recognition directly into a reduction in network connectivity. Noddeland et al. designed PEG hydrogels crosslinked with peptide sequences optimized for MMP responsiveness and loaded them with the anti-inflammatory JAK inhibitor tofacitinib citrate [122]. Exposure to MMP-9 triggered drug release, and repeated enzyme exposure was used to mimic inflammatory flare-ups in skin disease. Importantly, release depended on both the peptide crosslinker and polymer architecture, showing that enzymatic cleavage kinetics must be considered together with the underlying network structure.

Enzyme responsiveness can also be designed against pathogen-derived rather than host-derived enzymes, enabling therapeutic release specifically in the presence of infection. Vera-González et al. developed hydrogels containing peptide crosslinks susceptible to secreted aspartic proteases (Saps) produced by Candida albicans [123]. Exposure to fungal proteases degraded the responsive network and triggered release of encapsulated liposomal antifungal therapeutics. Because the triggering enzyme originated directly from the pathogen, cargo release was coupled to the presence of fungal activity rather than to a constitutive physiological variable. This strategy illustrates an important extension of endogenous-responsive delivery: biochemical signals produced by bacteria, fungi, or other pathogens can act as molecular indicators of disease burden and activate therapeutic release accordingly.

Disease-associated microenvironments rarely contain only one abnormal stimulus. Tumors combine extracellular acidity, elevated intracellular reducing capacity, oxidative stress, and increased protease activity; chronic wounds can exhibit abnormal pH, high ROS levels, bacterial or fungal enzymes, and elevated host proteases; and the gastrointestinal tract presents strong pH gradients together with site-specific digestive and microbial enzymes. Consequently, hydrogel responsiveness observed under isolated in vitro conditions may not translate directly to the more complex in vivo environment. A bond that is nominally pH responsive may also undergo hydrolysis over time, ROS-sensitive junctions may encounter heterogeneous oxidant concentrations, and protease-responsive peptides may be susceptible to more than one enzyme. The most effective systems therefore require response thresholds and degradation kinetics that distinguish pathological conditions from normal physiological fluctuations.

From a transport perspective, endogenous stimuli primarily regulate release by changing the fraction of intact crosslinks and, consequently, the accessibility of the network. Mild stimulation may increase bond exchange and relaxation without destroying the gel, thereby increasing effective diffusivity. Stronger or prolonged stimulation can decrease network connectivity sufficiently to cause swelling, degradation, and eventual erosion. Therapeutic release can therefore evolve from diffusion through a responsive but intact network to degradation-assisted and ultimately erosion-controlled transport. This continuum is particularly important in dynamic hydrogels because the same reversible chemistry can mediate self-healing under one condition and drug liberation under another.

Overall, endogenous stimuli-responsive release transforms pathological biochemical signals into changes in therapeutic transport. pH-sensitive bonds exploit acidity or physiological pH gradients, redox-responsive junctions respond to altered reducing conditions, ROS-sensitive chemistries couple oxidative stress to network degradation, and enzyme-cleavable crosslinks provide molecular recognition of disease-associated proteolytic activity. By tuning the threshold, kinetics, and reversibility of these responses, dynamic hydrogels can remain relatively stable during administration and subsequently increase therapeutic release within tumors, inflammatory tissues, gastrointestinal environments, wounds, or sites of infection. Such disease-adaptive behavior provides spatial control that complements the temporal control achievable with externally applied stimuli.

### 5.3. Temperature- and Externally Triggered Release

Whereas endogenous stimuli depend on biochemical conditions at the disease site, externally triggered hydrogels provide direct temporal control over therapeutic release. Temperature, light, ultrasound, magnetic fields, and electrical stimulation can alter polymer conformation, supramolecular association, crosslink stability, or local network permeability, allowing drug transport to be activated at a selected time after administration. An important advantage of these systems is that the trigger can often be applied repeatedly, making it possible to modulate dose and release frequency without replacing the implanted or injected depot. For dynamic hydrogels, external energy can additionally accelerate bond exchange or transiently disrupt reversible junctions, providing a direct connection between network dynamics and on-demand molecular transport.

Temperature-responsive hydrogels exploit reversible changes in polymer–polymer and polymer–water interactions around a critical solution temperature. In lower critical solution temperature (LCST) systems, increasing temperature promotes polymer association and dehydration, whereas upper critical solution temperature (UCST) behavior generally involves increased polymer solvation or weakening of intermolecular associations upon heating. These transitions can alter swelling, mesh accessibility, and cargo partitioning. Thermosensitive injectable hydrogels are particularly useful when a low-viscosity formulation can be administered at room temperature and subsequently form a more persistent depot near physiological temperature. Kim et al. developed a thermosensitive hydrogel based on Pluronic F127 and gelatin that formed gels at relatively low polymer concentrations and provided sustained delivery of an NO donor together with anti-CTLA-4 antibody [124]. The hybrid system is noteworthy because F127 provides thermally driven association while gelatin contributes UCST-related behavior and biodegradability, allowing temperature-dependent gel formation to be integrated with subsequent therapeutic release.

Temperature can also serve as an intermediate transduction mechanism for other external stimuli. Incorporating photothermal or magnetothermal components enables light or magnetic energy to be converted locally into heat, which subsequently changes the state of a thermoresponsive polymer network. This strategy avoids the need to heat the entire surrounding tissue and allows a relatively small region of the hydrogel to be actuated. The resulting release response may arise from network contraction, increased permeability, accelerated diffusion, or changes in cargo–polymer affinity, depending on the polymer architecture.

Light is particularly attractive because irradiation can be controlled spatially, temporally, and in intensity. Near-infrared (NIR) light is commonly combined with photothermal nanomaterials because conversion of photon energy into heat can actuate temperature-responsive hydrogel networks. Qu et al. incorporated gold nanorods into a NIPAm-containing thermo-responsive hydrogel loaded with doxorubicin [125]. NIR irradiation increased local temperature and induced contraction of the hydrogel network, accelerating DOX release. The system could be repeatedly irradiated in vivo and significantly reduced postoperative breast-tumor recurrence compared with the corresponding nonirradiated hydrogel. Importantly, this example illustrates that the photosensitive component does not necessarily need to form the polymer network itself; instead, nanomaterials can function as transducers that convert an externally delivered optical signal into a change in hydrogel transport properties.

Ultrasound provides a complementary external trigger with greater penetration into deep tissues. Acoustic energy can induce transient mechanical deformation, cavitation, local heating, or disruption of noncovalent network interactions. These effects can increase molecular transport without necessarily causing permanent destruction of the hydrogel. Huebsch et al. provided a representative demonstration using reversibly crosslinked alginate hydrogels in which divalent-ion-mediated junctions could be mechanically disrupted by focused ultrasound [126]. Ultrasound generated discrete bursts of encapsulated mitoxantrone release, after which the reversible network recovered its mechanical integrity. Repeated ultrasound exposure produced repeated release events, demonstrating that reversible junctions can function as mechanically resettable gates for externally controlled drug delivery. This mechanism is particularly relevant to dynamic hydrogels because self-recovery following stimulation enables multiple dosing cycles from the same depot.

Magnetic fields can regulate release through either mechanical actuation of embedded magnetic particles or magnetothermal heating under an alternating magnetic field. The latter approach is particularly compatible with thermoresponsive networks because magnetic nanoparticles convert electromagnetic energy into localized heat. Jalili et al. incorporated magnetic nanoparticles together with poly(N-isopropylacrylamide-co-acrylamide) nanogels into injectable GelMA-based hydrogels [127]. The resulting nanocomposite showed both temperature- and magnetic-field-dependent DOX release. Magnetic stimulation locally heated the network, altered the thermoresponsive state of the nanogel component, and increased therapeutic release, providing an externally controlled delivery platform without direct physical access to the hydrogel. The combination of reversible thermal transitions with remotely actuated magnetic particles illustrates how different material components can be integrated to separate the sensing/transduction function from cargo confinement.

Electrical stimulation offers another route to directly manipulate dynamic network organization. Depending on hydrogel chemistry, an applied electric field can induce ionic redistribution, electrophoretic cargo migration, redox reactions, osmotic swelling, or rearrangement of electrostatic and supramolecular interactions. Zhou et al. developed an electro-responsive dynamic hydrogel in which SDS micelles acted as reversible hydrophobic crosslinking domains [128]. Application of direct-current voltage rearranged the micelles and increased hydrogel mesh size, enabling release of the hydrophobic therapeutic cargo thiostrepton. Because the network was maintained by noncovalent interactions, the hydrogel also exhibited self-healing and malleability. This study provides a particularly direct example of how external electrical input can modify the architecture of a dynamic network rather than simply driving electrophoretic transport of a charged drug.

Electrically triggered systems can also couple drug release with wearable bioelectronic devices. Zhou et al. developed a redox-active viologen-containing polyelectrolyte hydrogel that functioned simultaneously as a drug reservoir and electrode in a Mg battery-powered iontophoresis patch [129]. As shown in Figure 7, the P(AM-co-SV) hydrogel was engineered as a mechanically robust, drug-loadable viologen-containing network, providing the material basis for its subsequent integration into an electrically controlled transdermal delivery platform.

Electrical reduction in viologen weakened electrostatic association with negatively charged dexamethasone, leading to voltage-dependent drug release. Only limited spontaneous release occurred without stimulation, whereas application of −0.6 or −1.0 V substantially increased release. Importantly, intermittent electrical activation generated controlled release over six days, demonstrating that dosage could be regulated through repeated on/off stimulation. Such systems illustrate how reversible electrochemical changes in polymer–cargo interactions can translate electronic signals directly into programmable molecular release.

Despite their advantages, externally triggered systems require careful coordination between stimulus intensity and network response. Excessive photothermal or magnetothermal heating can damage surrounding tissues, strong ultrasound may irreversibly disrupt the network, and high electrical potentials can produce unwanted electrochemical reactions. Conversely, stimulation that is too weak may produce insufficient changes in crosslink dynamics or permeability to substantially alter release. The optimal system should therefore exhibit a sufficiently large response within a biologically acceptable stimulus window. Reversibility is particularly valuable because the network can return toward its initial state after stimulation, reducing baseline leakage between treatment cycles.

Overall, temperature and external energy sources provide a means of converting dynamic hydrogels from environmentally responsive materials into actively controllable therapeutic depots. Thermoresponsive polymers translate temperature into changes in network organization, photothermal and magnetothermal components enable remote heating, ultrasound can transiently disrupt reversible junctions, and electrical signals can regulate network structure or polymer–cargo affinity. By matching the kinetics of external stimulation with bond exchange and network recovery, these systems can provide localized, repeatable, and temporally controlled therapeutic release. Such externally controlled behavior also provides an important foundation for the multistimuli, sequential, and programmable delivery systems discussed in the following section.

## 6. Therapeutic Cargoes and Biomedical Applications

Therapeutic cargoes differ substantially in size, charge, hydrophobicity, structural stability, and susceptibility to degradation; therefore, no single dynamic network architecture is optimal for all payloads. Small molecules, biologics, nucleic acids, and nanoparticle formulations require different balances of confinement, affinity, protection, and release kinetics. Dynamic hydrogels are particularly useful because reversible crosslinks and tunable interactions can be matched to these cargo-specific requirements. This section compares these design needs and their translation into major biomedical applications. The principal cargo-specific challenges and corresponding hydrogel design requirements are summarized in Table 5.

### 6.1. Small-Molecule and Biologic Drug Delivery

Dynamic hydrogels can accommodate therapeutic cargoes spanning several orders of magnitude in molecular size, but effective delivery requires network properties to be matched to the physicochemical characteristics of each cargo. Small-molecule drugs generally benefit from straightforward incorporation and rapid equilibration within hydrated networks, yet these same properties can lead to weak retention and burst release. Proteins, peptides, growth factors, and cytokines present the opposite challenge: their larger dimensions favor physical retention but increase sensitivity to network architecture, while conformational instability and susceptibility to enzymatic degradation place additional constraints on loading chemistry. Dynamic networks provide considerable flexibility because physical confinement, reversible affinity, dynamic covalent attachment, and controlled degradation can be combined without relying exclusively on permanent immobilization.

Anticancer drugs represent one of the most extensively investigated small-molecule cargos in dynamic hydrogel systems. Doxorubicin (DOX), for example, has been incorporated into self-healing imine- and acylhydrazone-crosslinked networks in which local retention can be combined with pH-dependent network destabilization [116,117]. Beyond single-drug delivery, dynamic hydrogels are well suited to co-delivering molecularly dissimilar therapeutics. Wang et al. developed an injectable supramolecular hydrogel that simultaneously delivered DOX and kynureninase (KYNase), a therapeutic enzyme that degrades the immunosuppressive metabolite kynurenine [130]. The two cargos displayed markedly different release timescales: approximately 80% of DOX was released within 24 h, whereas the denser hydrogel formulation sustained KYNase release for approximately 15 days. The authors attributed the slower release of KYNase relative to small molecules to its substantially higher molecular weight. Thus, a single dynamic network can produce intrinsically differentiated release profiles when cargos differ strongly in molecular dimensions, providing a useful basis for combining rapid cytotoxic activity with prolonged biological modulation.

Antibiotic delivery poses a related but distinct requirement. Local antimicrobial therapy generally requires sufficiently high drug availability to suppress microbial growth while retaining the agent at the affected tissue for longer than would be achieved by free administration. Mondal et al. prepared a chitosan–α-naphthaldehyde hydrogel film in which reversible imine linkages generated a biodegradable network capable of encapsulating the fluoroquinolone antibiotics ciprofloxacin and lomefloxacin [131]. The hydrogel exhibited high antibiotic encapsulation and controlled release, while incorporation of pineapple leaf fibers reinforced the otherwise relatively weak chitosan-based network. This example highlights an important cargo-specific design principle: because many conventional antibiotics are small and highly mobile, increasing retention does not necessarily require a very dense permanent network. Instead, dynamic crosslinking, drug–polymer interactions, and controlled network degradation can be combined to extend local exposure while preserving hydration and conformability.

Anti-inflammatory agents similarly benefit from strategies that suppress rapid escape but must often be delivered over considerably longer therapeutic windows. Chauhan et al. used oxidized pullulan and eight-arm PEG-hydrazine to construct injectable self-healing hydrogels through dynamic hydrazone bonds [132]. Rather than simply entrapping dexamethasone (Dex), the drug was covalently linked to PEG through a hydrolyzable hydrazone linkage, converting reversible covalent chemistry into an additional level of cargo retention. The resulting network sustained PEG–Dex conjugate release for 28 days and exhibited faster release at pH 6.5 than at pH 7.4. This architecture illustrates the distinction between manipulating the hydrogel network and manipulating the cargo itself: reversible drug conjugation can suppress premature diffusion even when the surrounding hydrogel remains highly hydrated.

An alternative strategy is to eliminate the distinction between therapeutic cargo and carrier. Zheng et al. demonstrated that the natural anti-inflammatory small molecule rhein could directly self-assemble into a three-dimensional nanofibrous hydrogel through π–π stacking, hydrogen bonding, and other noncovalent interactions [48]. The resulting carrier-free hydrogel exhibited sustained release and reversible responses to shear and temperature while producing longer-lasting anti-neuroinflammatory activity than the corresponding free drug. Because therapeutic molecules themselves constituted the dynamic network, release was governed by gradual dissociation of supramolecular assemblies rather than diffusion from an inert polymer matrix. Such drug-based supramolecular hydrogels are particularly attractive for poorly soluble small molecules because they can simultaneously increase local drug content and minimize the fraction of nontherapeutic carrier material.

Biologic cargoes impose a different set of constraints. Growth factors, cytokines, enzymes, antibodies, and therapeutic peptides generally require aqueous and chemically mild loading conditions to preserve tertiary structure and biological activity. Their larger dimensions often provide better physical retention than small drugs, but simple steric entrapment can still be insufficient when prolonged delivery is required. Moreover, increasing network density to slow protein diffusion may impair injectability or delay complete release. Dynamic hydrogels therefore commonly employ reversible electrostatic, host–guest, or other affinity interactions to decouple protein retention from bulk network stiffness. As demonstrated previously, multivalent electrostatic interactions can regulate protein residence, while host–guest avidity can be tuned to control the lifetime of protein–network association [105]. The relevant design variable is consequently not merely protein size but the combined effect of molecular dimensions, charge distribution, affinity, and the lifetime of the binding interaction.

Growth factors provide a representative example because therapeutic activity commonly depends on sustained local signaling rather than a high initial dose. Yaguchi et al. developed a jigsaw-shaped self-assembling peptide (JigSAP) that forms supramolecular nanofibers through dynamic peptide self-assembly and functions as an artificial extracellular matrix [133]. The system efficiently incorporated vascular endothelial growth factor (VEGF) and provided sustained release while maintaining regenerative activity in mouse stroke models. The design mimicked the ability of natural extracellular matrices to reversibly retain secreted proteins rather than immobilizing VEGF permanently. This type of affinity-mediated supramolecular delivery is advantageous for growth factors because excessive binding would suppress receptor availability, whereas insufficient interaction would result in rapid loss from the treatment site.

Cytokine delivery presents a similar retention problem but additionally requires careful control of local concentration because systemic exposure can produce undesirable biological effects. Chen et al. addressed this challenge using a composite system in which IL-10 was encapsulated within degradable hyaluronic acid microgels that were subsequently dispersed in an injectable adamantane–cyclodextrin guest–host hydrogel [134]. The supramolecular bulk matrix provided shear-thinning injection and rapid self-recovery, whereas the internal microgels independently controlled IL-10 release through their crosslinker-dependent degradation. In a rat myocardial infarction model, IL-10-loaded composites reduced macrophage density and improved several measures of cardiac repair compared with saline treatment. Importantly, the hierarchical structure separated the functions of administration and tissue retention from those of cytokine release, illustrating how dynamic hydrogels can incorporate secondary reservoirs when direct protein loading cannot provide the required release timescale.

Therapeutic proteins further illustrate why cargo-specific behavior must be considered during hydrogel design. In the supramolecular system of Wang et al., encapsulation did not eliminate the catalytic activity of KYNase, and increasing hydrogel polymer content prolonged enzyme release and the associated depletion of kynurenine in the tumor microenvironment [130]. Antibody delivery presents an even more demanding case because immunoglobulins combine large hydrodynamic dimensions with structurally sensitive binding domains; consequently, degradation-controlled [108], affinity-controlled [105], or nanoparticle-assisted delivery [92] may be preferable to simple physical encapsulation. Likewise, therapeutic peptides and peptide hormones may occupy an intermediate transport regime in which molecular size alone is insufficient for retention, making reversible affinity or responsive network interactions important for maintaining therapeutically useful concentrations.

Collectively, these examples demonstrate that cargo identity should be treated as a primary design variable rather than as a component introduced after hydrogel optimization. Hydrophilic small molecules generally require stronger retention mechanisms to prevent rapid diffusion, whereas hydrophobic drugs may benefit from supramolecular or hydrophobic domains that improve both loading and residence. Reversible covalent attachment can further extend small-molecule release when simple encapsulation is insufficient. For proteins and peptides, mild loading, preservation of conformational activity, controlled affinity, and eventual complete release become more important than maximizing initial encapsulation alone. Growth factors and cytokines often require sustained local availability at relatively low concentrations, while therapeutic enzymes and antibodies additionally demand preservation of functional structure over the lifetime of the depot.

Accordingly, the optimal dynamic hydrogel does not necessarily maximize crosslink density or cargo–polymer affinity. Excessively weak interactions lead to premature loss, whereas overly persistent association can reduce the bioavailable fraction and prevent complete therapeutic recovery. Effective delivery instead requires matching cargo size, charge, hydrophobicity, binding affinity, and biological stability to network mesh accessibility, bond-exchange kinetics, degradation, and depot lifetime. These considerations become still more important for nucleic acids, which require protection against extracellular degradation and efficient intracellular transport, motivating the hierarchical nanoparticle–hydrogel strategies discussed in the following section.

### 6.2. Nucleic Acid and Nanoparticle–Hydrogel Delivery Systems

Nucleic acids represent one of the therapeutic classes that most clearly benefit from hierarchical hydrogel-based delivery. Unlike many small molecules and proteins, siRNA, mRNA, plasmid DNA, and gene-editing components must overcome both extracellular and intracellular barriers before exerting their biological function. Rapid nuclease degradation, unfavorable charge and molecular size for membrane transport, inefficient endosomal escape, and limited tissue residence collectively restrict direct administration. Dynamic hydrogels can address the tissue-scale component of these barriers by maintaining therapeutic material at the target site, but they generally cannot provide efficient intracellular delivery by themselves. Consequently, nucleic acids are frequently incorporated into polymeric, lipid, liposomal, or inorganic nanocarriers before being embedded within a hydrogel depot. This creates a hierarchical system in which the nanoparticle protects and transports the genetic cargo at the cellular scale, whereas the hydrogel regulates localization and release at the tissue scale.

For siRNA, electrostatic complexation with cationic polymers provides protection against premature degradation but can also result in rapid dissociation or loss of silencing activity if release is poorly controlled. Cao et al. addressed this limitation by complexing siRNA with chitosan methacrylate and encapsulating the complexes within liposomes to produce crosslinked nanolipogels [135]. Incorporation of a crosslinked nanogel core substantially reduced the initial siRNA burst and extended release for up to 28 days, while gene silencing remained detectable for up to 14 days in vitro. Importantly, the lipid shell facilitated cellular uptake while the internal crosslinked polymer compartment regulated siRNA liberation, illustrating how nanoscale hydrogel and lipid components can perform complementary functions within the same carrier.

At the macroscopic scale, embedding siRNA or RNA nanoparticles within a secondary hydrogel can introduce another level of temporal control. Previous systems discussed in this review have demonstrated sustained siRNA delivery from self-healing networks [136], while host–guest hydrogels have prolonged the local activity of microRNA nanocomplexes. A more explicit nanoparticle–hydrogel architecture was demonstrated by Li et al., who complexed miR-140 with a modified generation-5 polyamidoamine vector (G5-AHP) and subsequently incorporated the resulting nanoparticles into injectable GelMA hydrogel microspheres [137]. The hydrogel microspheres increased local nanoparticle retention and underwent MMP-responsive degradation, whereas the released G5-AHP/miR-140 nanoparticles retained efficient cellular internalization and gene-regulatory activity. Following intra-articular administration, this “nano–micron” system prolonged gene-vector residence and attenuated cartilage degeneration in an osteoarthritis model. As illustrated in Figure 8, this hierarchical “nano–micron” platform integrates miR-140-loaded G5-AHP nanocomplexes with injectable GelMA microspheres, enabling local retention, MMP-responsive release, and subsequent intracellular delivery of the therapeutic nucleic acid.

Messenger RNA presents related but distinct challenges because therapeutic efficacy requires delivery of intact, translation-competent RNA into the cytoplasm. Poly(β-amino ester) (pBAE) nanoparticles have therefore been incorporated into injectable hydrogels to combine intracellular transfection with sustained local delivery. Duran-Mota et al. developed a biodegradable PEG:pBAE hydrogel containing mRNA-loaded pBAE polyplexes for local gene delivery to human dermal fibroblasts [138]. The polymeric nanoparticles protected the mRNA and provided the intracellular delivery function, while the surrounding hydrogel acted as a reservoir that sustained nanoparticle release over therapeutically relevant periods. This separation of functions is particularly useful for wound and regenerative applications, where local gene expression may need to persist longer than the residence time of freely administered nanoparticles.

Lipid-based carriers can be incorporated into hydrogels according to the same principle. Gao et al. developed an injectable thermosensitive chitosan hydrogel containing lipid nanoparticles carrying IRF5 mRNA together with CCL5 siRNA for local pancreatic cancer immunotherapy [139]. The RNA-loaded lipid nanoparticle complex provided intracellular gene regulation, whereas the chitosan hydrogel restricted the formulation to the tumor region and provided local sustained delivery. The combined modulation increased pro-inflammatory M1-like macrophage polarization and promoted antitumor immune responses while avoiding reliance on systemic distribution of the RNA formulation. This system demonstrates an important advantage of hydrogel–LNP integration: the high intracellular delivery efficiency of lipid nanoparticles can be retained while their biodistribution is shifted from systemic circulation toward a localized therapeutic depot.

Hybrid inorganic–lipid nanoparticles provide another means of coordinating nucleic-acid protection with biomaterial functionality. Andrée et al. investigated local mRNA delivery using nanocomposites composed of gelatin and hydroxyapatite nanoparticles and compared mRNA complexed with peptide-, lipid-, and lipid-coated calcium phosphate (LCP)-based transfection systems [140]. In the LCP formulation, calcium phosphate provided an inorganic core for mRNA association, whereas an outer lipid layer containing an ionizable lipid facilitated cellular delivery. Incorporation into the gelatin/hydroxyapatite biomaterial maintained local mRNA delivery and protein expression, illustrating how inorganic nanoparticles can serve simultaneously as structural components of a biomaterial and as nucleic-acid delivery vehicles. Such systems expand nanoparticle–hydrogel design beyond conventional polymeric carriers by allowing inorganic phases to contribute mechanical, regenerative, or ion-releasing functions in addition to cargo transport.

DNA delivery generally tolerates longer extracellular residence than mRNA but introduces additional barriers associated with the large size of plasmids and the requirement for nuclear entry. Nanoparticle complexation is therefore commonly used to condense DNA and enhance cellular uptake, while hydrogels can maintain the vectors within the target tissue. More advanced systems integrate DNA delivery with gene-editing machinery. Chen et al. developed liposome-templated hydrogel nanoparticles (LHNPs) for co-delivery of CRISPR/Cas9 components [141]. These core–shell nanoparticles accommodated Cas9 protein together with nucleic acids and were combined with minicircle DNA technology to deliver CRISPR/Cas9 targeting polo-like kinase 1 (PLK1). The platform produced efficient gene editing and suppressed tumor growth in vivo, including after engineering the particles for brain-tumor targeting. Although this system operates primarily at the nanoscale rather than as a macroscopic depot, it demonstrates how hydrogel-like polymer compartments within lipid carriers can overcome the fundamentally different physicochemical requirements of protein and nucleic-acid components within a single gene-editing formulation.

This distinction becomes increasingly important for CRISPR-based therapies. A gene-editing payload may contain Cas protein, sgRNA, mRNA encoding a nuclease, or DNA encoding one or more editing components, each with different stability and intracellular trafficking requirements. Direct encapsulation of all components within a bulk hydrogel is therefore unlikely to provide optimal cellular delivery. A more rational architecture is to encapsulate the gene-editing machinery within a nanoscale carrier and then use an injectable dynamic hydrogel as a secondary depot. The nanoparticle can be engineered for endosomal escape and intracellular disassembly, whereas the hydrogel controls regional retention, exposure time, and potentially the sequence in which different carriers are released. The CRISPR LHNP system [141], together with RNA nanoparticle–hydrogel systems [138,139,140], provides a conceptual foundation for this multiscale approach.

Nanoparticle–hydrogel systems are not limited to nucleic acids. Polymeric nanoparticles can function as secondary drug reservoirs within dynamic networks, as illustrated by the PLGA nanoparticle-containing chitosan hydrogel described in Section 5.1 [114]. Liposomes and lipid nanoparticles can improve loading of hydrophobic cargos or protect labile biomolecules, whereas inorganic nanoparticles can introduce photothermal, magnetic, imaging, or catalytic functions. In each case, incorporation into the hydrogel adds a second level of retention: cargo must first leave or be released with the nanoparticle and subsequently traverse the surrounding polymer network. Depending on nanoparticle–polymer interactions, the hydrogel may also directly restrict particle mobility, allowing carrier release to occur on a substantially longer timescale than molecular diffusion alone.

Mesoporous nanoparticles are particularly attractive in this context because their internal pores provide a distinct nanoscale loading compartment that can be combined with the adaptive properties of dynamic hydrogels. Mohamed et al. loaded colchicine into mesoporous silica nanoparticles (MSNs) and subsequently incorporated either free or MSN-encapsulated colchicine into a self-healing hydrogel formed from carboxyethyl chitosan and oxidized pullulan [142]. The composite was further integrated into cotton patches for transdermal osteoarthritis therapy and produced extended drug release compared with less hierarchical formulations. Although the cargo in this case was a small molecule rather than a nucleic acid, the architecture illustrates the general principle of serial transport barriers: mesoporous nanoparticles regulate loading and primary release, while the surrounding dynamic hydrogel controls particle localization and secondary transport. This strategy is readily extensible to biomacromolecules when pore dimensions, surface chemistry, and cargo stability are appropriately matched.

The major advantage of such hybrid systems is therefore functional separation across length scales. The nanoparticle can be optimized for cargo complexation, nuclease protection, cellular uptake, endosomal escape, or stimulus-responsive intracellular release without requiring those functions to be encoded into the bulk hydrogel. Conversely, the hydrogel can be optimized for injectability, self-healing, tissue adhesion, local persistence, and gradual nanoparticle release without compromising nanoparticle structure. This modularity is particularly important for nucleic-acid therapeutics, because increasing RNA–polymer affinity sufficiently to prevent extracellular loss can simultaneously hinder intracellular release. Encapsulating RNA within a dedicated carrier and then controlling the carrier rather than the naked nucleic acid through the hydrogel provides a means of avoiding this tradeoff.

However, nanoparticle incorporation does not automatically guarantee sustained therapeutic delivery. Nanoparticles substantially smaller than the hydrogel mesh may diffuse rapidly from the network unless they interact with polymer chains or are physically trapped within secondary domains. Conversely, excessively strong nanoparticle–network association may prevent complete carrier release. The relative rates of cargo release from the nanoparticle, nanoparticle transport through the hydrogel, hydrogel relaxation or degradation, and intracellular cargo release therefore create a cascade of transport timescales. The most effective systems deliberately separate these timescales so that the hydrogel provides prolonged tissue residence while the released nanoparticles retain sufficient mobility and integrity for cellular uptake.

Overall, nucleic-acid and nanoparticle–hydrogel systems illustrate one of the strongest advantages of dynamic hydrogel drug delivery: the ability to construct multiscale therapeutic depots in which distinct material components perform distinct transport functions. Polymeric carriers can protect and condense RNA or DNA, lipid nanoparticles and liposomes enhance membrane transport, inorganic nanoparticles introduce structural and functional properties, and mesoporous carriers provide high-capacity secondary reservoirs. The surrounding hydrogel adds localization, sustained release, mechanical adaptability, and, when dynamic crosslinks are employed, responsiveness to biological or external stimuli. Such hierarchical architectures are particularly promising for siRNA, mRNA, DNA, and gene-editing technologies because they bridge the gap between nanoscale intracellular delivery and macroscale spatiotemporal control, thereby providing a versatile platform for localized and sustained gene therapy.

### 6.3. Major Biomedical Applications

The biomedical value of dynamic hydrogel drug-delivery systems becomes most apparent when their material properties are matched to the specific constraints of a disease or tissue environment. Injectability enables minimally invasive placement into irregular defects or surgical cavities, reversible crosslinks permit adaptation and recovery after deformation, tissue adhesion improves local retention, and degradation or stimulus responsiveness can coordinate therapeutic release with disease progression. These characteristics have enabled dynamic hydrogels to function not merely as passive drug reservoirs but as locally adaptive therapeutic matrices across cancer treatment, wound and infection management, transdermal delivery, and regenerative medicine.

Cancer therapy represents a particularly compelling application because localized hydrogel depots can maintain high therapeutic concentrations near tumors while limiting systemic exposure. As described in previous sections, dynamic hydrogels have been used to deliver cytotoxic agents, therapeutic enzymes, immunomodulators, antibodies, and RNA-based therapeutics [130,140]. Their advantages become especially important after surgical tumor removal, when residual malignant cells may remain within an irregular resection cavity that is difficult to treat using conventional implants. Injectable dynamic networks can conform to these cavities and subsequently provide localized release during the period when recurrence risk remains high.

Zhao et al. demonstrated this concept in postoperative glioma therapy using an injectable matrix-metalloproteinase-responsive hydrogel containing temozolomide (TMZ) and the O^6^-methylguanine-DNA methyltransferase inhibitor O^6^-benzylguanine [143]. The system was designed to respond to elevated MMP activity following glioma surgery, promoting local release of both agents into the resection region. Co-delivery inhibited MGMT-mediated TMZ resistance and improved suppression of residual glioma compared with TMZ treatment alone. This strategy illustrates an important advantage of postoperative dynamic depots: biochemical changes generated by surgery and residual disease can themselves regulate therapeutic availability rather than relying exclusively on predetermined passive release.

Related approaches combine local chemotherapy with externally triggered treatment. The thermoresponsive gold-nanorod-containing hydrogel discussed in Section 5.3 provided NIR-enhanced doxorubicin release and photothermal therapy to suppress postoperative breast-cancer recurrence [125]. Likewise, nanoparticle–hydrogel systems can maintain therapeutic carriers at the tumor site while enabling controlled release and additional photothermal, immune, or gene-regulatory functions [121,130,140]. Collectively, these examples demonstrate that the principal benefit of dynamic hydrogels in cancer is not simply prolonged drug residence; rather, the local depot can integrate cavity filling, therapeutic retention, stimulus responsiveness, and multimodal treatment within a single injectable platform.

Dynamic hydrogels are equally well suited to wound healing and localized infection therapy, where the material must remain in close contact with an irregular and mechanically active tissue surface while simultaneously controlling microbial growth and supporting regeneration. Reversible networks are advantageous because they can deform with the wound, recover after mechanical disruption, and maintain coverage despite movement. Moreover, dynamic coordination or covalent bonds can be combined with antibacterial components and pro-regenerative therapeutics. Chen et al. developed an injectable self-healing hydrogel based on reversible Ag–S coordination between multi-arm thiolated PEG and Ag^+^ [144]. The dynamic coordination network provided injectability and self-recovery, while released silver ions supplied antibacterial activity. Loading the angiogenic agent desferrioxamine further promoted vascularization, and the combined system accelerated repair of diabetic skin wounds in vivo.

This multifunctionality is particularly important in infected wounds, where bacterial suppression alone does not necessarily restore a regenerative microenvironment. Sun et al. developed an injectable mussel-inspired hydrogel based on oxidized konjac glucomannan, dopamine/L-cysteine-modified poly(γ-glutamic acid), and ε-polylysine [145]. Reversible thiol–aldehyde addition and Schiff-base reactions generated a self-healing dynamic network, while ε-polylysine and the overall formulation provided antibacterial and antioxidant functions. The hydrogel inhibited both Pseudomonas aeruginosa and Staphylococcus aureus, adhered to wound tissue, reduced inflammatory responses, and accelerated repair in an infected burn-wound model. In this context, dynamic crosslinking contributes directly to therapeutic function by allowing the dressing to conform to irregular defects and restore network integrity after mechanical damage, thereby maintaining continuous protection during healing.

Localized infection therapy can also be integrated with drug delivery rather than relying on intrinsic antibacterial polymers or ions. Antibiotic-containing dynamic hydrogel systems such as the chitosan-based formulations described in Section 6.1 can prolong antimicrobial exposure [131], while nanoparticle or liposome reservoirs can further separate initial antibacterial action from longer-term tissue repair. This modularity is useful because infection and regeneration often dominate at different stages of wound healing. An effective dynamic dressing can therefore provide rapid antimicrobial activity initially while gradually shifting toward anti-inflammatory, angiogenic, or regenerative functions as the network reorganizes or secondary cargos are released.

Transdermal drug delivery imposes a different design requirement. Here, the hydrogel must maintain intimate contact with the skin while controlling drug partitioning and transport across the stratum corneum. Adhesive and self-healing networks are useful because they maintain conformal contact during bending and movement, whereas electrically, thermally, or chemically responsive networks can provide an additional driving force for molecular transport. Zhou et al. demonstrated electrically controlled transdermal delivery using a dynamic micelle-crosslinked hydrogel in which electrical stimulation reorganized reversible hydrophobic domains and promoted thiostrepton release; transdermal transport was subsequently demonstrated across porcine skin [128]. Thus, dynamic network rearrangement can regulate not only release from the hydrogel but also the amount of drug made available at the skin interface.

Hierarchical hydrogel–nanoparticle systems can further address the poor solubility or limited skin penetration of certain drugs. Mohamed et al. incorporated colchicine-loaded mesoporous silica nanoparticles into a self-healing carboxyethyl-chitosan/oxidized-pullulan hydrogel and subsequently integrated the composite into cotton patches for osteoarthritis treatment [142]. The MSN reservoir and surrounding dynamic hydrogel provided sequential barriers to drug escape, extending release while maintaining a localized transdermal formulation. Such designs are particularly relevant when free small molecules would otherwise diffuse rapidly from a hydrated patch, because the nanoparticle controls primary loading and release while the hydrogel maintains intimate tissue contact and secondary transport control.

In regenerative medicine and tissue repair, the role of dynamic hydrogels extends beyond drug release because network remodeling itself can influence cell migration, vascularization, and extracellular-matrix formation. Unlike permanently crosslinked scaffolds, reversible networks can rearrange as cells exert forces and remodel their surroundings, potentially allowing tissue ingrowth without requiring rapid wholesale degradation. Hsieh et al. developed an injectable chitosan–fibrin self-healing hydrogel that degraded gradually while supporting vascular endothelial-cell organization and capillary formation [146]. Remarkably, injection of the hydrogel without exogenous angiogenic growth factors promoted vascularization and restored blood circulation in an ischemic hindlimb model, demonstrating that the structural and biological composition of a dynamic matrix can itself provide regenerative cues.

Regeneration can also be enhanced by using the dynamic hydrogel as a local reservoir for bioactive carriers. Liu et al. incorporated BMP-2-loaded adhesive liposomes into an injectable PEG hydrogel formed through reversible Ag–S coordination [147]. The dynamic hydrogel provided minimally invasive filling of osteoporotic bone defects, whereas released liposomes adhered to surrounding tissue and maintained BMP-2 activity locally. The composite promoted bone mineralization and reconstruction in an osteoporotic fracture model. This hierarchical design is particularly instructive because the hydrogel and liposome perform distinct functions: the hydrogel provides mechanical adaptability, local retention, and antibacterial activity, whereas the adhesive nanocarrier extends residence of the regenerative protein after leaving the bulk network.

Similar principles extend to cartilage, neural, and soft-tissue regeneration. Growth-factor-containing supramolecular peptide hydrogels can sustain pro-regenerative signaling [133], while dynamic nanoparticle-containing chitosan networks have provided prolonged release of cartilage-regenerative agents [114]. The therapeutic hydrogel should therefore not be viewed solely as a scaffold or solely as a delivery vehicle. Instead, network relaxation, degradation, biological ligand presentation, and therapeutic release collectively determine how cells interact with the material over time. An initially cohesive network may protect the defect and retain cargo, followed by increasing relaxation or degradation that permits cell infiltration, matrix deposition, and eventual replacement of the synthetic depot by regenerated tissue.

Across these biomedical applications, the same dynamic network property can serve different purposes. Rapid self-recovery may maintain coverage in a moving wound, enable syringe delivery into a tumor cavity, preserve skin contact in a transdermal patch, or allow a regenerative scaffold to accommodate tissue deformation. Likewise, controlled degradation may determine drug-release duration in cancer therapy but simultaneously create space for tissue ingrowth during regeneration. The optimal dynamic hydrogel must therefore be designed around the therapeutic context rather than a universally desirable set of material properties.

Overall, dynamic hydrogels provide an adaptable interface between therapeutic cargo and diseased tissue. In cancer and postoperative therapy, they create localized depots that conform to resection cavities and reduce systemic drug exposure; in wound and infection management, they combine physical protection with antimicrobial and regenerative functions; in transdermal systems, they maintain tissue contact while regulating molecular delivery across the skin; and in regenerative medicine, they coordinate therapeutic release with progressive tissue remodeling. These applications demonstrate that the greatest potential of dynamic hydrogel drug delivery lies in integrating localization, adaptability, controlled release, and biological function within a single temporally evolving material.

### 6.4. Biomedical Applications by Administration Route

The therapeutic value of dynamic hydrogels depends not only on the disease being treated but also on the route of administration and the functional requirements imposed by the target tissue. Injectable local depots must combine minimally invasive delivery with rapid structural recovery and prolonged retention, whereas topical and transdermal systems require conformal tissue contact and controlled interfacial transport. Implantable and regenerative systems place greater emphasis on gradual degradation, mechanical adaptation, and coordination between therapeutic release and tissue remodeling. The following sections therefore organize representative applications according to these therapeutic and administration requirements rather than individual disease categories.

#### 6.4.1. Injectable and Local Depot Delivery

Injectable dynamic hydrogels are particularly useful when therapeutics must be retained within irregular tissue defects, surgical cavities, tumors, or other localized sites. The major design requirements are shear-thinning or in situ gelation during administration, rapid recovery after injection, sufficient mechanical persistence to prevent premature clearance, and controlled release over the required therapeutic period. These requirements are especially important in cancer therapy, where local depots can maintain high drug concentrations near the treatment site while limiting systemic exposure. Postoperative applications additionally benefit from the ability of dynamic networks to conform to resection cavities and respond to biochemical or externally applied signals [140,143]. Representative systems illustrate that the principal advantage of injectable dynamic hydrogels is not simply prolonged residence. Responsive networks can couple therapeutic release to local disease-associated signals, whereas nanoparticle-containing hydrogels can combine localized treatment with gene-regulatory or other complementary functions [140,143]. However, these benefits require a balance between dynamicity and persistence. Networks that recover rapidly after injection may still lose structural integrity or cargo too quickly if reversible junctions are excessively labile, whereas overly stable networks can impair injectability, adaptation to the tissue space, or complete therapeutic release. Local-depot design should therefore match network recovery, degradation, and release timescales to the intended duration of treatment.

#### 6.4.2. Topical and Transdermal Delivery

Topical and transdermal applications impose a different set of requirements because the hydrogel must maintain continuous contact with a mechanically active tissue surface. For wound and infection management, adhesion, self-healing, conformability, and resistance to repeated deformation are important for maintaining coverage, while antimicrobial activity and controlled therapeutic release may be required simultaneously. Dynamic networks can therefore combine mechanical recovery with antibacterial and regenerative functions [144]. For transdermal delivery, the hydrogel must additionally regulate drug availability at the skin interface and, where necessary, facilitate transport across the stratum corneum. Stimuli-responsive dynamic networks can modulate drug release and transdermal transport, whereas nanoparticle–hydrogel systems can provide sequential transport barriers that improve retention of rapidly diffusing or poorly soluble cargos [147]. Effective topical and transdermal platforms therefore require a balance among tissue contact, mechanical recovery, cargo retention, and interfacial transport.

#### 6.4.3. Implantable and Regenerative Delivery

Regenerative applications require dynamic hydrogels to function simultaneously as therapeutic depots and temporally evolving matrices. Unlike purely local drug reservoirs, these materials must often permit cell infiltration, vascularization, matrix deposition, and progressive replacement by newly formed tissue. Consequently, network relaxation and degradation must be coordinated with tissue-remodeling timescales rather than optimized solely to maximize drug retention. Dynamic injectable matrices have been shown to support vascularization and tissue repair, while hydrogel–nanocarrier systems can prolong the local activity of regenerative therapeutics [145,146]. The critical design challenge is to coordinate mechanical adaptation, therapeutic retention, and material degradation. Degradation that is too rapid can cause premature loss of mechanical support and therapeutic cargo, whereas overly persistent networks can restrict tissue ingrowth and delay material replacement. Dynamic hydrogel design for regenerative medicine should therefore balance depot persistence with the progressive structural remodeling required for successful tissue repair. Across these administration routes, the same dynamic property can serve different therapeutic functions. Rapid self-recovery supports syringe delivery in local depots, preserves coverage in moving wounds, and accommodates deformation in regenerative matrices. Likewise, controlled degradation may determine drug-release duration in a localized depot while simultaneously creating space for tissue ingrowth during regeneration. Dynamic hydrogel design should therefore be guided by the specific therapeutic requirement and administration route rather than by a universally optimal combination of stiffness, relaxation, degradation, or cargo retention.

## 7. Current Challenges

Despite substantial progress, translation of dynamic hydrogels remains limited by the difficulty of linking molecular exchange kinetics to reproducible mechanical, transport, and in vivo performance [148,149]. Reversible networks are inherently sensitive to formulation, environmental, and manufacturing variables, making predictability and standardization central challenges.

A fundamental limitation is the difficulty of predicting dynamic network behavior across molecular and macroscopic scales. In idealized networks, the kinetics and thermodynamics of reversible junctions can be related quantitatively to macroscopic relaxation. Marco-Dufort et al. demonstrated that dynamic covalent networks provide a framework in which molecular exchange kinetics can be connected with experimentally observed viscoelastic properties [149]. However, practical drug-delivery hydrogels are substantially more heterogeneous than ideal networks. Spatial variations in crosslink density, incomplete functionalization, polymer molecular-weight distributions, local phase separation, entanglements, and competing intra- and intermolecular interactions can all alter the fraction of elastically active junctions. These effects become particularly important in dynamic systems because small changes in association equilibrium or bond lifetime can propagate into large changes in relaxation, yielding, injectability, and transport behavior.

Supramolecular networks illustrate this sensitivity particularly well. Zou et al. systematically altered host–guest affinity while maintaining a related network architecture and demonstrated that crosslink affinity translated directly into markedly different bulk dynamic properties [150]. Stronger interactions produced networks with increasingly persistent, covalent-like behavior, whereas weaker associations facilitated faster rearrangement. More recently, Claiborne et al. demonstrated that competitive molecular interactions can be incorporated into a quantitative framework for predicting the mechanical properties of dynamic boronate-ester hydrogels [151]. Their model related competitor concentration and binding affinity to an apparent crosslink association constant and achieved close agreement between predicted and measured modulus over a broad range of competitor affinities. Such studies are important because they move dynamic hydrogel development from empirical formulation toward predictive network design. Nevertheless, extending these models to heterogeneous polymer networks containing therapeutic cargoes, proteins, salts, nanoparticles, and biological fluids remains considerably more difficult.

Batch-to-batch variability consequently deserves greater attention than is commonly given in proof-of-concept hydrogel studies. For dynamic covalent systems, relatively small differences in the degree of aldehyde oxidation, hydrazide or thiol substitution, boronic-acid functionalization, or polymer molecular weight can change the effective stoichiometry of network formation. Supramolecular materials can additionally be sensitive to host or guest substitution levels and the presence of competing molecules. Such variations may not be evident from polymer mass concentration alone but can substantially alter relaxation and drug retention. Future studies should therefore report not only nominal polymer concentrations but also critical molecular attributes such as functionalization degree, molecular-weight distribution, reactive-group conversion, crosslink stoichiometry, and relevant equilibrium or kinetic parameters whenever possible. Establishing these variables as critical material attributes would substantially improve reproducibility across laboratories and provide a more useful basis for eventual manufacturing control [149].

An equally important challenge is the limited ability to infer drug-release behavior from rheological measurements alone. Storage modulus, loss modulus, yield stress, recovery, and stress-relaxation time provide valuable information regarding network structure and dynamics, but none directly represents therapeutic mobility. As demonstrated in the transport studies discussed in Part 4, cargo diffusion additionally depends on molecular size relative to mesh accessibility [102], reversible cargo–network binding [105], and progressive degradation or erosion [23,96]. Thus, two hydrogels with similar G′ values can potentially exhibit substantially different release profiles if their crosslinks differ in lifetime or if the therapeutic molecule interacts differently with the polymer. Conversely, two materials with different mechanical moduli can produce comparable therapeutic retention when affinity interactions dominate transport. A more predictive approach will require coordinated characterization in which rheology, molecular diffusion, binding kinetics, degradation, and release are measured under comparable conditions rather than treated as independent properties. Key material properties, representative characterization methods, and their relevance to drug-delivery performance are summarized in Table 6.

This issue becomes even more pronounced in vivo. Dynamic networks encounter proteins, salts, enzymes, reducing agents, ROS, competing ligands, mechanical deformation, fluid exchange, immune-cell interactions, and cellular uptake that are absent or poorly represented in conventional buffer-based release experiments. These factors can shift reversible binding equilibria, screen electrostatic interactions, accelerate bond cleavage or degradation, compete for host–guest or metal–ligand binding sites, and alter network relaxation, erosion, and cargo mobility. Immune-cell infiltration and phagocytic activity may further influence local degradation and material clearance. Consequently, in vitro cumulative release curves provide only part of the information needed to predict in vivo pharmacokinetics. Future studies should therefore evaluate network dynamics and therapeutic release under conditions that more closely reproduce the biochemical and mechanical environment of the target tissue and relate these measurements to local residence time, tissue distribution, bioavailable drug concentration, and pharmacodynamic duration rather than evaluating release exclusively by the fraction of cargo recovered in a sink solution [149].

A second major design problem is the intrinsic tradeoff between mechanical stability and dynamicity. Rapidly exchanging junctions facilitate stress relaxation, self-healing, conformability, and stimulus-responsive transport, but sufficiently fast dissociation can also reduce mechanical persistence and accelerate material clearance. Conversely, highly stable crosslinks increase depot lifetime and cargo retention but may suppress relaxation, injectability, or responsiveness. The results of Zou et al. [150] and Marco-Dufort et al. [149] demonstrate that crosslink thermodynamics and kinetics strongly influence macroscopic material behavior, underscoring why stability and dynamicity cannot generally be optimized independently. An ideal injectable drug depot must therefore exhibit fast enough rearrangement to permit injection and adaptation to the tissue environment while maintaining sufficient connectivity to remain localized during the intended therapeutic period.

Importantly, the relevant timescales may differ by several orders of magnitude. Shear thinning during injection occurs over seconds, self-recovery may occur over seconds to minutes, tissue adaptation and stress relaxation may extend over minutes to hours, whereas therapeutic retention can be required for days or weeks. Designing all of these behaviors through a single reversible bond is difficult. One promising strategy is therefore to construct networks containing multiple levels of dynamics, in which rapidly exchanging physical or supramolecular junctions govern short-term mechanical adaptation while slower dynamic covalent bonds or secondary interactions maintain long-term structural integrity. Hierarchical nanoparticle–hydrogel systems described throughout this review provide another route, because drug retention can be controlled by a secondary carrier rather than requiring the bulk network itself to remain highly restrictive. In this manner, mechanical dynamics and therapeutic retention can be partially decoupled rather than optimized through the same structural variable.

The same reasoning applies to burst release, which remains a persistent limitation of hydrogel-based drug delivery. Immediately after implantation or injection, freely dissolved cargo near the hydrogel surface can rapidly partition into surrounding fluid before the network reaches equilibrium. Dynamic networks may be particularly susceptible when transient crosslink rearrangement initially creates highly accessible transport pathways. Network swelling, dilution following contact with tissue fluid, incomplete cargo–polymer association, and early-stage degradation can further amplify this process. A large initial burst may deplete the depot prematurely and produce local or systemic concentrations above the intended therapeutic range.

Strategies for suppressing burst release should therefore focus on reducing the initially mobile cargo fraction rather than merely increasing bulk modulus. Multivalent affinity interactions can prolong protein retention while preserving a hydrated network [105], whereas incorporation of drug-loaded nanoparticles creates serial transport barriers that reduce direct molecular escape [114]. Increasing cargo affinity must nevertheless be balanced against complete eventual release, because excessively strong binding can convert burst suppression into irreversible sequestration. Similarly, increasing crosslink density can reduce initial diffusion but may compromise injectability or degradation. The most robust solution is likely to combine moderate network confinement with reversible affinity and/or a secondary carrier so that no single mechanism must provide the entire retention function.

Biocompatibility presents another critical challenge because the safety of a dynamic hydrogel cannot be inferred solely from the biocompatibility of its base polymer. The complete formulation may contain reactive functional groups, crosslinking catalysts or initiators, residual small molecules, unreacted precursors, incorporated nanomaterials, and, in metal-coordinated systems, free metal ions released through ligand exchange or network degradation. In addition, dynamic bond exchange and hydrogel degradation can generate soluble polymer fragments or reactive intermediates whose biological effects may differ from those of the intact material. These concerns are particularly important for in situ-forming systems because network formation occurs in direct contact with tissue. Therefore, safety evaluation should consider not only acute cell viability but also the cytotoxicity of individual precursors and degradation products, inflammatory and immune responses, local accumulation or systemic distribution of soluble components, and their eventual clearance. Chemistries that proceed efficiently in aqueous media without highly reactive catalysts or harsh conditions are preferable, but even nominally mild dynamic reactions should be evaluated at clinically relevant concentrations and over timescales that reflect the intended duration of implantation [149].

Manufacturing and scale-up introduce additional challenges that are often less apparent during small-scale laboratory preparation. Many dynamic hydrogel systems require multi-step polymer functionalization, precise control over the degree of functionalization and crosslink stoichiometry, and, in some cases, oxygen- or moisture-sensitive reactions, catalysts, or specialized crosslinkers. Each additional synthetic or formulation step can introduce variability in reactive-group conversion, residual reagents, gelation kinetics, and final network properties. Dynamic hydrogels are also frequently formed by mixing two or more precursor solutions immediately before use; therefore, mixing order, precursor concentration, pH, ionic strength, mixing ratio, mixing energy, temperature, and the interval between formulation and administration can become critical process parameters during scale-up. Complex hybrid systems containing nanoparticles, liposomes, or multiple dynamic components introduce further sources of variability, including particle size, loading efficiency, component distribution, particle–polymer association, sedimentation, and aggregation during storage. Practical translation will therefore require simplification of synthetic and formulation steps where possible, definition of critical material attributes and acceptable process ranges, and standardized specifications for gelation, rheological behavior, degradation, and drug release to ensure batch-to-batch reproducibility under GMP-compatible manufacturing conditions.

Sterilization is particularly challenging because methods that reliably remove microbial contamination may also alter polymer molecular weight, crosslink density, drug stability, or network mechanics. The extent of this effect is highly material dependent. Sceglovs et al. systematically examined steam sterilization of covalently crosslinked ε-polylysine/hyaluronic-acid hydrogels and found that appropriately formulated networks could retain much of their physicochemical, viscoelastic, and antibacterial performance following treatment [152]. In contrast, the broader sterilization literature demonstrates that a sterilization process cannot be assumed to be inert toward every hydrogel formulation. Drug-containing systems present an additional complication because the therapeutic molecule itself may be more sensitive than the polymer. Topete et al., for example, compared sterilization strategies for drug-loaded hydrogel-based intraocular lenses and showed that high hydrostatic pressure could sterilize the system while largely preserving material and drug-delivery performance, whereas gamma irradiation could damage drugs under some formulation conditions [153]. These findings emphasize that sterilization must be developed together with the final formulation rather than selected only after hydrogel optimization.

Storage stability raises similar concerns. Dynamic crosslink precursors may undergo hydrolysis, oxidation, aggregation, or gradual loss of reactive functionality before administration, whereas preformed dynamic networks may continue to rearrange during storage. Consequently, a formulation that performs reproducibly immediately after synthesis may not exhibit the same gelation kinetics or release after months of storage. Translation will therefore require shelf-life studies that monitor not only visual stability and drug potency but also molecular weight, functional-group integrity, crosslinking kinetics, rheology, injectability, and release profiles. For multicomponent systems, physically separating reactive precursors until administration, lyophilizing unstable components, or using dual-chamber delivery devices may ultimately be more practical than attempting to store a fully equilibrated dynamic network.

## 8. Future Perspectives

These challenges also define several promising directions for the next generation of dynamic hydrogel therapeutics. One important shift will be from empirically optimized responsive materials toward programmable networks in which molecular inputs generate quantitatively predictable outputs. The feedback-responsive glucose/H_2_O_2_ system described in Section 5.2 [120] already demonstrates how a physiological signal can be chemically converted into network degradation and therapeutic release, whereas ultrasound- and electrically controlled systems provide externally programmable dosing [126,129]. Future materials could integrate multiple thresholds and timescales so that one stimulus alters network permeability, another controls degradation, and a third regulates cargo affinity. Rather than simply being “responsive,” such networks would perform predefined material operations in response to their environment.

Feedback-controlled release represents an especially compelling goal. In an ideal adaptive depot, a disease-associated signal would increase therapeutic availability when treatment is needed and decrease release as the pathological state resolves. Achieving this behavior requires more than incorporating a cleavable bond: the stimulus concentration must be related quantitatively to the magnitude and kinetics of network response, and the hydrogel must avoid complete irreversible depletion during a transient pathological episode. Reversible signal transduction, competing binding equilibria, catalytic reaction networks, and dynamically regenerated junctions may therefore become increasingly important. The quantitative competitive-inhibition framework reported by Claiborne et al. [151] illustrates how dynamic chemistry can already be modeled to predict macroscopic behavior, providing a conceptual step toward networks whose responses are engineered rather than discovered empirically.

Artificial intelligence and machine learning offer another route to address the unusually large design space of dynamic hydrogel systems. Even a relatively simple formulation can vary in polymer identity, molecular weight, functionalization, concentration, crosslinker chemistry, stoichiometry, cargo loading, and environmental conditions, creating a multidimensional parameter space that is inefficient to search experimentally. Kumar et al. demonstrated that machine learning could predict and inversely design poly(2-oxazoline) compositions with user-defined cloud points, experimentally validating 17 previously unreported polymer formulations [154]. More recently, Liao et al. combined data mining, experimentation, and machine learning to optimize hydrogel formulations from a dataset of 180 bioinspired hydrogels, producing materials with substantially improved adhesive performance [155]. These studies were not specifically designed around drug-release kinetics, but they demonstrate that complex polymer and hydrogel structure–property relationships can be explored using data-driven approaches.

For dynamic drug-delivery networks, the greatest value of AI may arise when chemistry, rheology, transport, degradation, and biological response are incorporated into the same structured dataset. Rather than optimizing a single property such as modulus, future models could search for formulations satisfying multiple constraints simultaneously—for example, syringe injectability, relaxation within minutes, depot retention for two weeks, less than a defined initial burst, and complete drug recovery within a specified therapeutic window. Achieving this goal will require standardized experimental descriptors and sufficiently large, interoperable datasets. Negative results and formulations that fail to gel or release appropriately may be particularly valuable because they define the boundaries of the design space.

Data-driven optimization may also support personalized hydrogel therapeutics. Disease-associated pH, ROS levels, enzyme activity, wound exudate, tumor geometry, and required therapeutic duration vary among patients and disease stages. A future formulation platform could therefore select polymer composition, crosslink density, responsive threshold, and cargo dose according to patient-specific biological inputs rather than relying on a single fixed material for every case. Injectable and in situ-forming dynamic hydrogels are particularly compatible with this concept because network properties can often be adjusted by formulation rather than by changing the geometry of a prefabricated implant. However, personalization will be clinically feasible only if formulation algorithms operate within validated composition ranges and manufacturing workflows capable of producing patient-specific materials without sacrificing quality control.

Ultimately, the next stage of dynamic hydrogel drug delivery will depend less on discovering additional reversible crosslink chemistries and more on achieving predictable integration across scales. Molecular bond kinetics must be connected to network relaxation, network relaxation to therapeutic transport, therapeutic transport to local pharmacokinetics, and pharmacokinetics to biological outcome. At the same time, materials must remain manufacturable, sterilizable, stable during storage, and safe after complete degradation. Progress toward these goals will require closer integration of polymer chemistry, rheology, transport modeling, pharmacology, manufacturing science, and data-driven materials design. Dynamic hydrogels have already demonstrated that reversible networks can provide sustained, responsive, and on-demand therapeutic delivery; the principal challenge now is to transform this versatility into reproducible and clinically controllable behavior. If these barriers can be addressed, programmable and adaptive hydrogel depots could evolve from laboratory-scale responsive materials into a broader platform for feedback-regulated, patient-tailored, and precision therapeutic delivery.

## 9. Conclusions

Dynamic hydrogels provide a versatile platform for controlled drug delivery because reversible crosslinks couple molecular-scale interaction dynamics with time-dependent network behavior. A central finding of this review is that hydrogel performance cannot be predicted solely from static parameters such as stiffness, swelling, or nominal crosslink density. Instead, bond-exchange kinetics, network relaxation, mesh accessibility, degradation, and cargo–network interactions collectively determine therapeutic transport and release.

The reviewed studies further demonstrate that drug release from dynamic hydrogels is rarely governed by a single mechanism. Rather, diffusion, reversible affinity, network relaxation, and degradation or erosion act together, with their relative importance depending on both the dynamic network and the physicochemical properties of the therapeutic cargo. Consequently, no single dynamic crosslinking strategy is universally optimal. Small molecules, proteins and peptides, nucleic acids, and nanoparticle-based therapeutics require different balances of confinement, affinity, protection, and release kinetics, while administration routes such as injectable local delivery, topical or transdermal delivery, and regenerative implantation impose additional mechanical and biological requirements.

Overall, effective dynamic hydrogel design requires coordinated control of molecular interaction kinetics, network properties, cargo characteristics, and therapeutic context. At the same time, practical translation will depend on improving predictability, biocompatibility, manufacturing reproducibility, sterilization, storage stability, and scale-up. Establishing quantitative relationships across these levels will be essential for advancing dynamic hydrogels from adaptable laboratory materials toward reliable and clinically relevant drug-delivery systems.

## Figures and Tables

**Figure 1:**
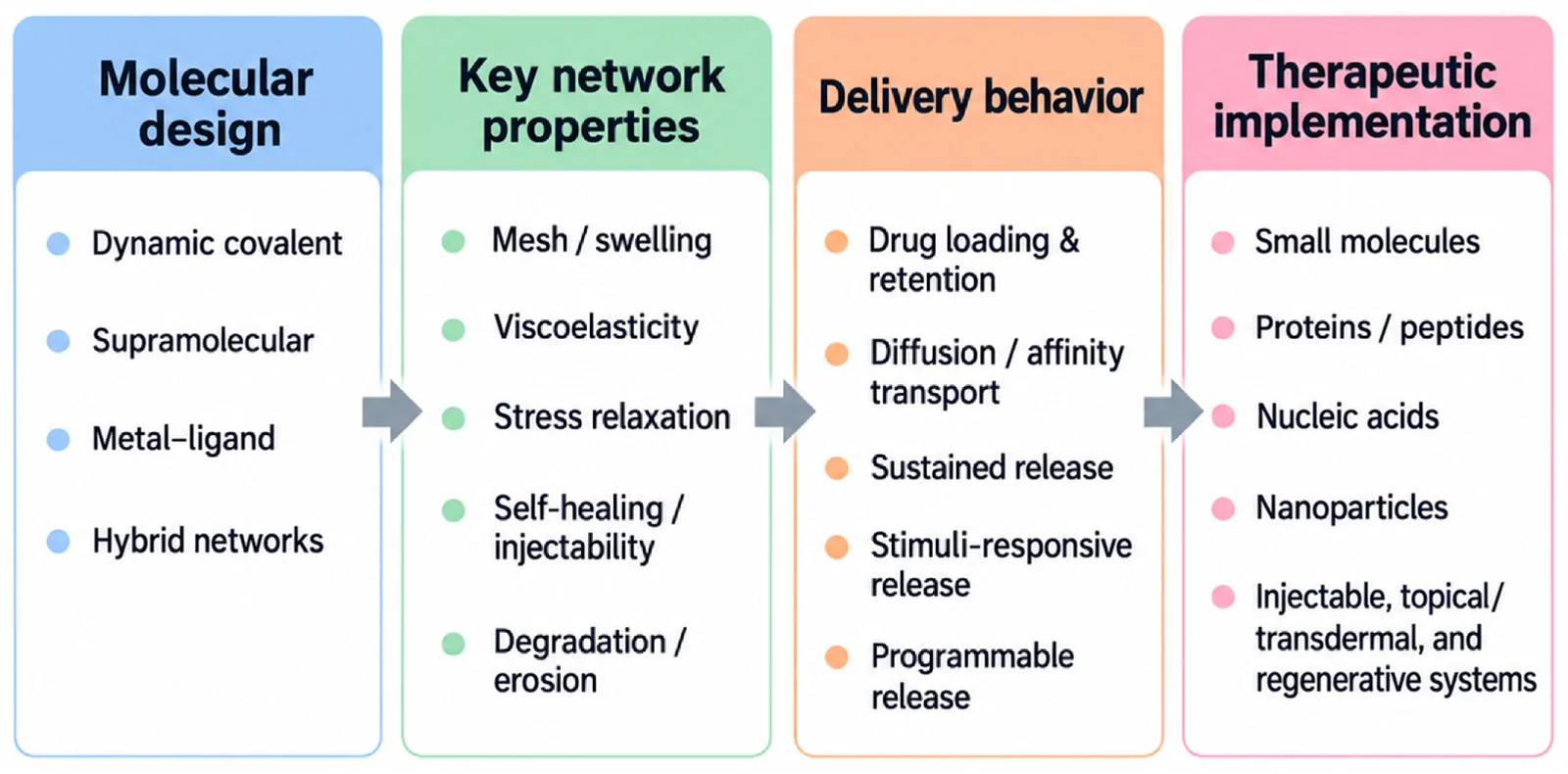
Overview of the design logic of dynamic hydrogel systems for controlled drug delivery. Dynamic hydrogel systems are organized according to molecular design strategy, key network properties, delivery behavior, and therapeutic implementation.

**Figure 2:**
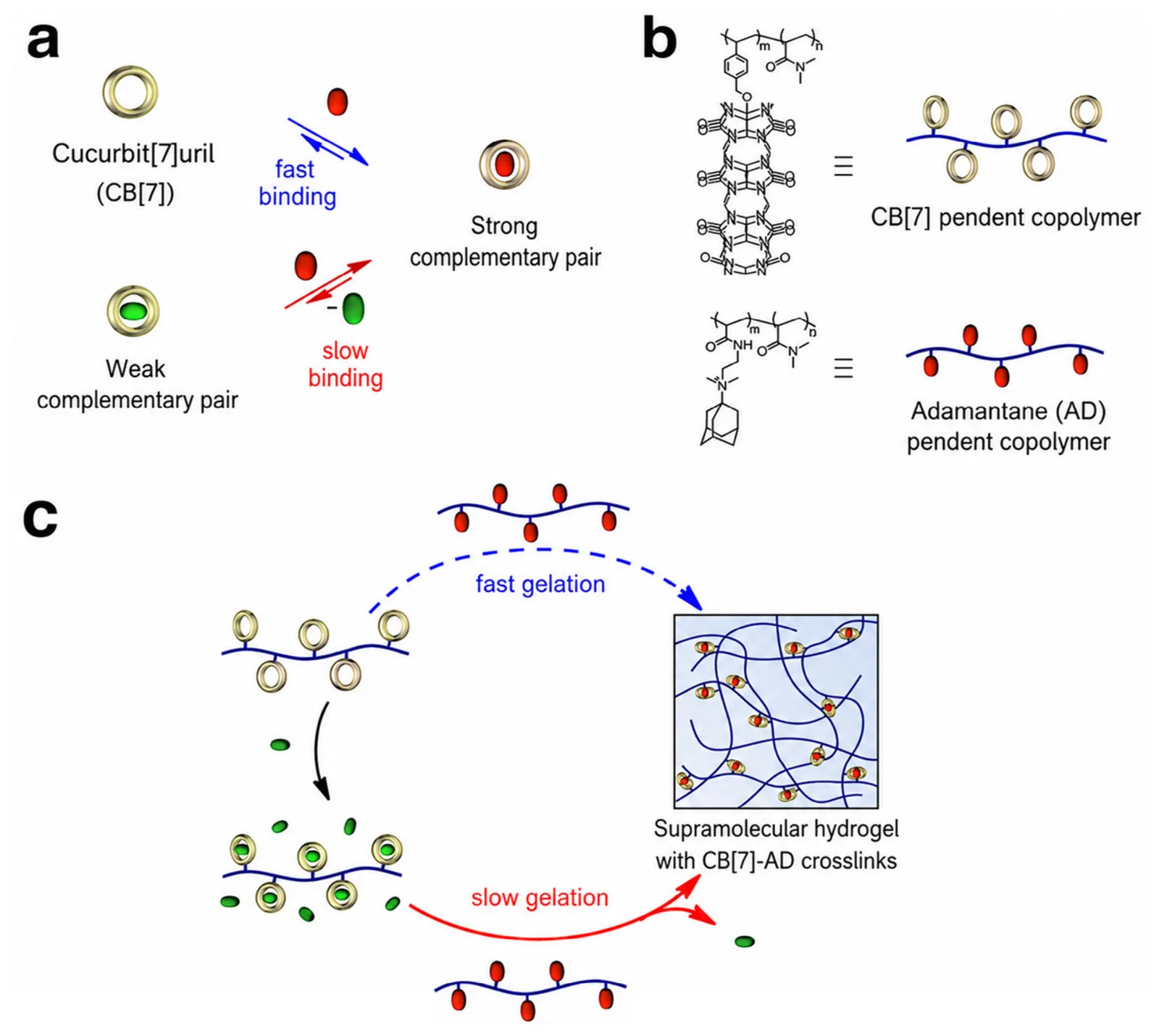
Competitive regulation of gelation kinetics in cucurbit[7]uril (CB[7])–adamantane (AD) supramolecular hydrogels. (**a**) Rapid formation of the strong CB[7]–AD host–guest complex and competitive inhibition by a weaker guest that transiently occupies the CB[7] cavity. (**b**) Chemical structures and schematic representations of the CB[7]-pendent and AD-pendent copolymers. (**c**) Modulation of hydrogel gelation kinetics by pre-complexing CB[7] with weak competing guest molecules, which delays formation of CB[7]–AD crosslinks. Adapted from Ref. [47] under the terms of the Creative Commons Attribution 4.0 International License.

**Figure 3:**
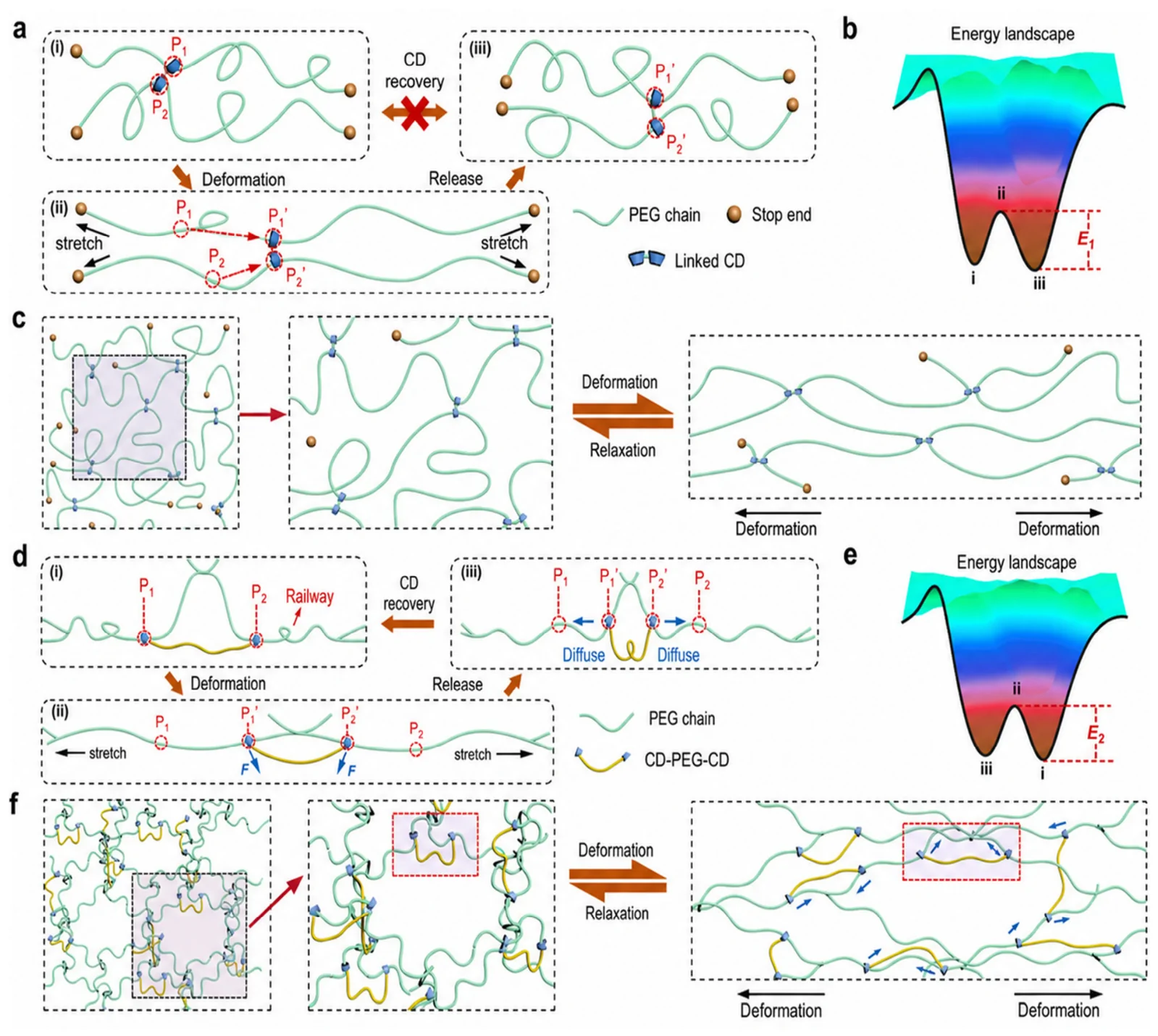
Molecular and network mechanisms underlying rapid damping through chain-walker-mediated molecular friction. (**a**) Schematic representation of cyclodextrin (CD) sliding along end-free PEG chains in a conventional slide-ring hydrogel during deformation and relaxation; the CD rings may not fully recover their original positions after stress relaxation. (**b**) Corresponding free-energy landscape illustrating the energy barrier (*E*_1_) associated with CD displacement. (**c**) Network-level representation of deformation and relaxation in a conventional slide-ring hydrogel. (**d**) Schematic representation of CD chain walkers sliding along end-fixed PEG chains, where molecular sliding dissipates mechanical energy and the chain walkers subsequently recover their positions through diffusion. (**e**) Corresponding free-energy landscape with the characteristic energy barrier (*E*_2_). (**f**) Network-level representation of a covalently crosslinked four-arm PEG hydrogel containing mobile chain walkers during repeated deformation and relaxation, with blue arrows indicating the sliding directions of the chain walkers along the PEG chains. Adapted from Ref. [86] under the terms of the Creative Commons Attribution 4.0 International License.

**Figure 4:**
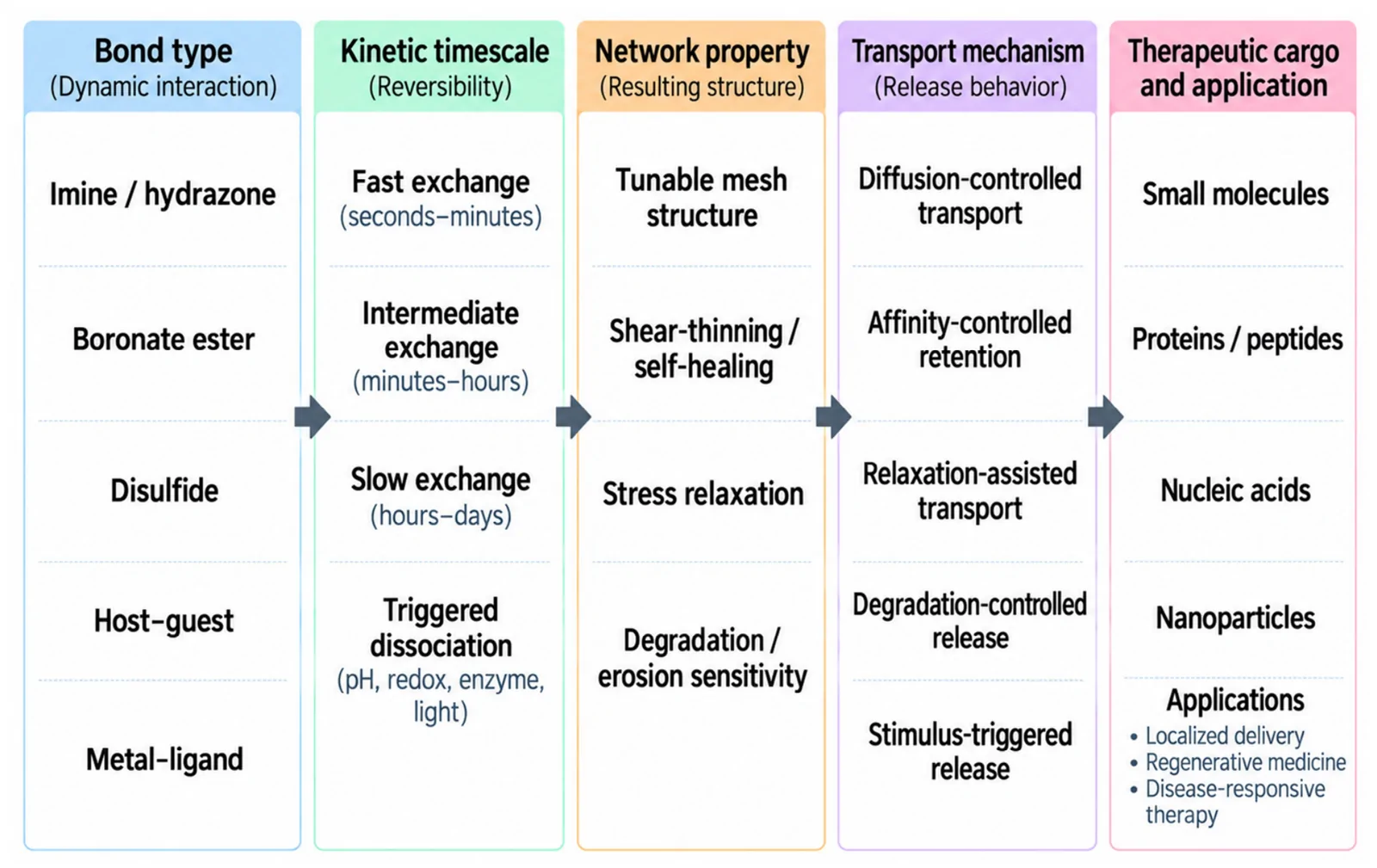
Conceptual framework linking dynamic crosslink chemistry to network properties, molecular transport, and therapeutic applications. Dynamic interactions with different exchange kinetics and reversibility govern network adaptation, including mesh rearrangement, stress relaxation, self-healing, and degradation, which subsequently determine diffusion-, affinity-, relaxation-, degradation-, and stimulus-controlled therapeutic transport.

**Figure 5:**
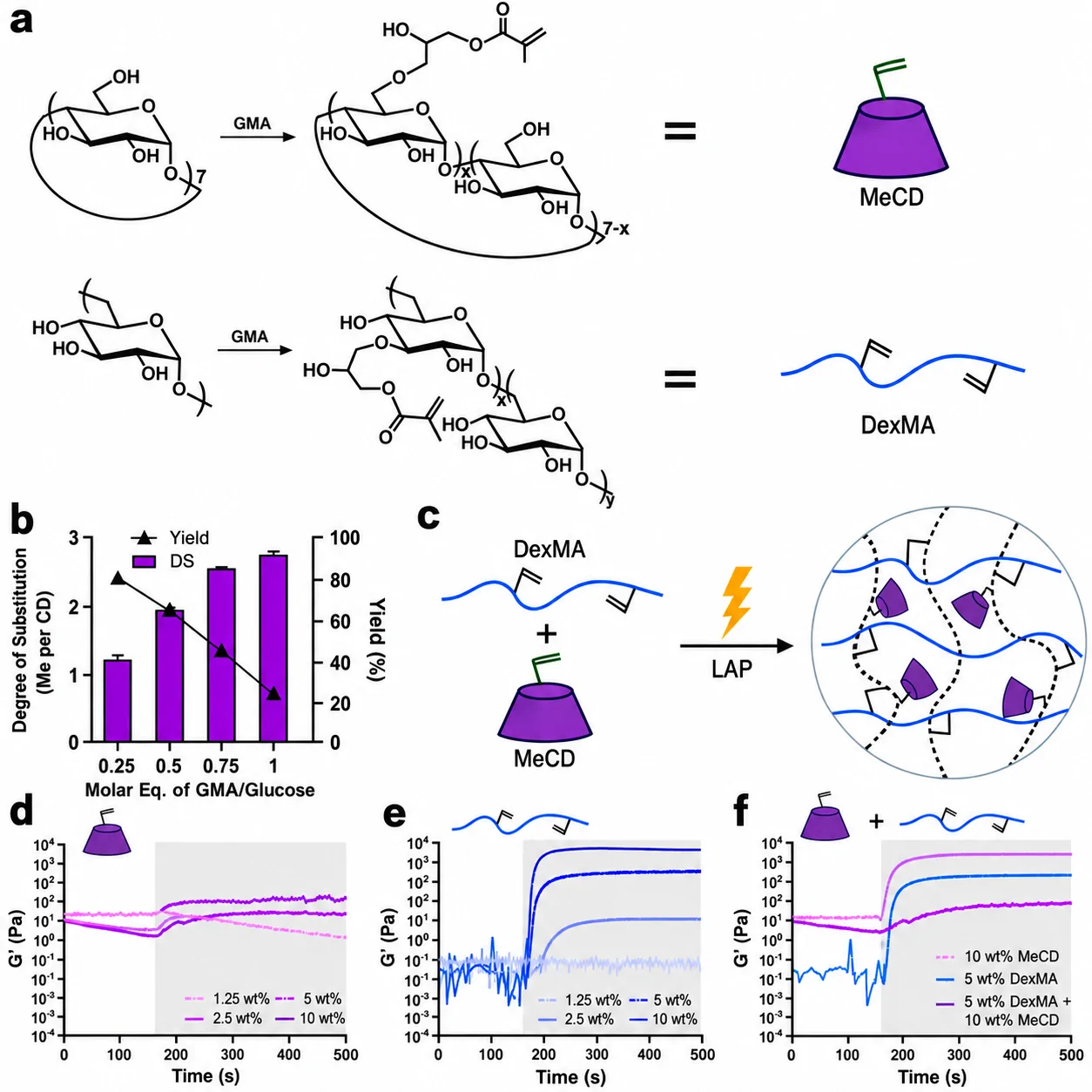
Preparation and characterization of cyclodextrin-containing hydrogel networks for avidity-controlled biotherapeutic delivery. (**a**) Synthesis of methacrylated cyclodextrin (MeCD) and methacrylated dextran (DexMA) through glycidyl methacrylate (GMA)-mediated functionalization. (**b**) Dependence of the degree of substitution (DS) and reaction yield on the molar feed ratio of GMA to glucose repeat units. (**c**) Formation of MeCD/DexMA hydrogels by photopolymerization in the presence of lithium phenyl-2,4,6-trimethylbenzoylphosphinate (LAP). Representative oscillatory time sweeps showing the evolution of storage modulus (*G*′) during photopolymerization of (**d**) MeCD alone, (**e**) DexMA alone, and (**f**) combined MeCD/DexMA formulations. Adapted from Ref. [106] under the terms of the Creative Commons Attribution 4.0 International License.

**Figure 6:**
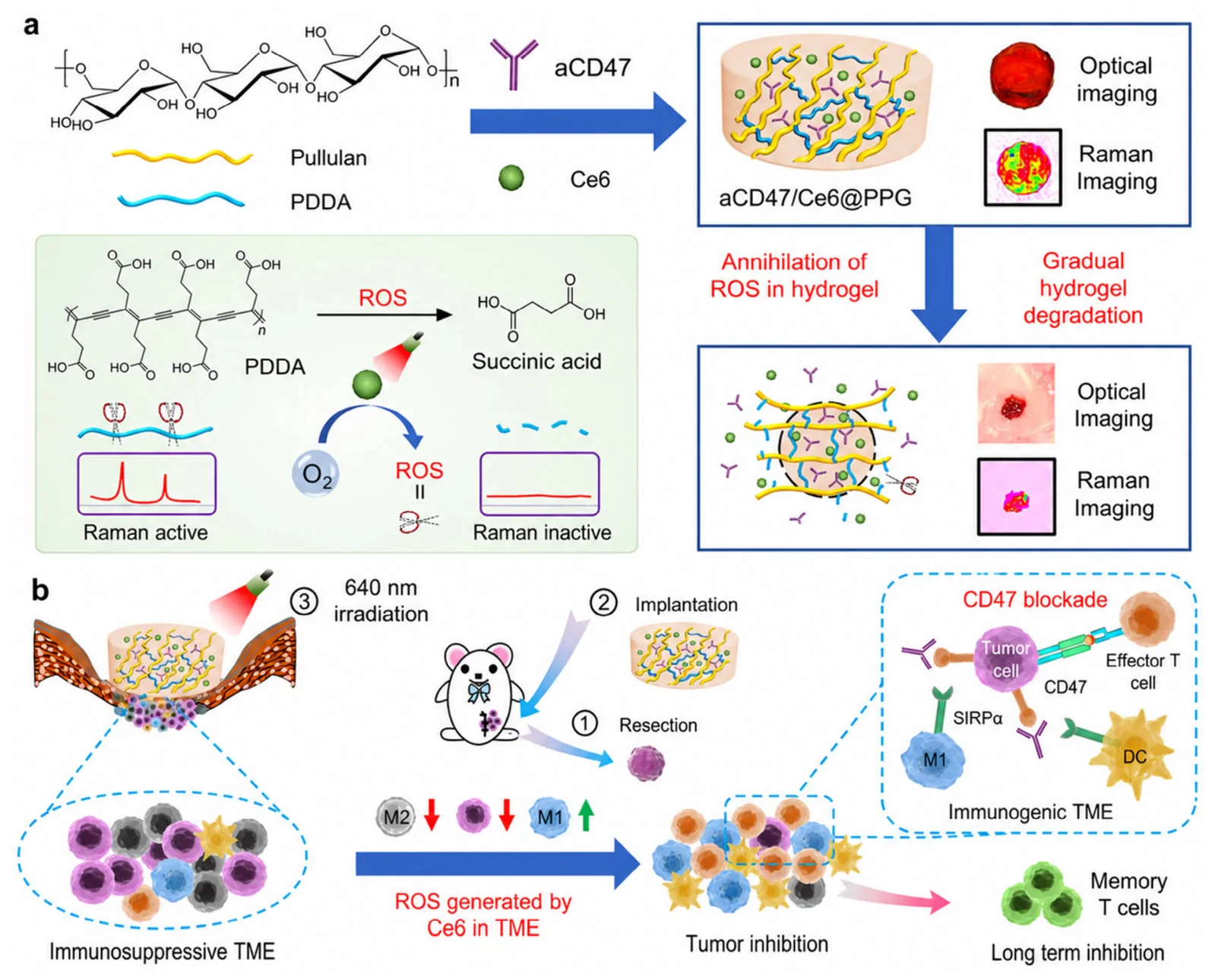
ROS-responsive hydrogel integrating photodynamic and immune therapy for postsurgical cancer treatment. (**a**) Schematic construction of the anti-CD47 antibody (aCD47)- and chlorin e6 (Ce6)-loaded aCD47/Ce6@PPG hydrogel and its ROS-responsive, Raman-traceable degradation. ROS-mediated oxidation of the PDDA component promotes hydrogel degradation while altering its Raman signal, enabling simultaneous monitoring of material degradation. (**b**) Schematic representation of the therapeutic strategy in a postsurgical 4T1 tumor model. Following implantation and 640 nm irradiation, Ce6-generated ROS promotes hydrogel degradation and therapeutic release, while photodynamic therapy and CD47 blockade cooperate to remodel the immunosuppressive tumor microenvironment and enhance antitumor immune responses. Adapted from Ref. [122] under the terms of the Creative Commons Attribution 4.0 International License.

**Figure 7:**
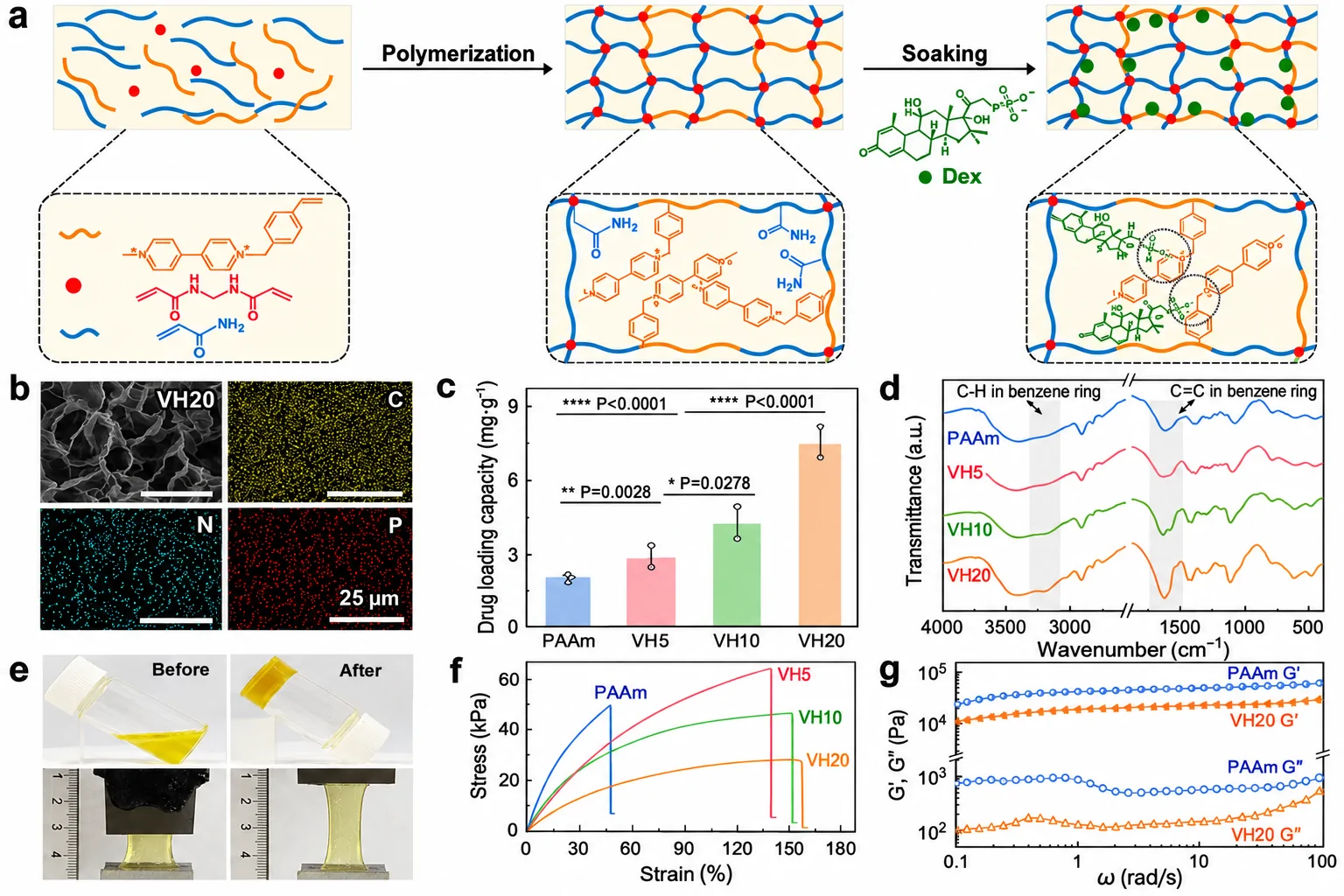
Preparation and characterization of drug-loaded P(AM-co-SV) hydrogels for electrically controlled transdermal drug delivery. (**a**) Schematic representation of hydrogel synthesis, network formation, dexamethasone (Dex) loading, and molecular interactions within the drug-loaded P(AM-co-SV) network. (**b**) Scanning electron microscopy (SEM) image and corresponding energy-dispersive X-ray spectroscopy (EDS) elemental maps of the Dex-loaded VH20 hydrogel. (**c**) Drug-loading capacities of PAAm and P(AM-co-SV) hydrogels containing different amounts of viologen. (**d**) FT-IR spectra of PAAm and P(AM-co-SV) hydrogels with increasing viologen content. (**e**) Representative photographs showing gelation and stretching of the VH20 hydrogel. (**f**) Tensile stress–strain curves of PAAm and P(AM-co-SV) hydrogels. (**g**) Angular-frequency-dependent oscillatory rheological behavior of PAAm and VH20 hydrogels. Statistical significance for panel (c): * *p* = 0.0278, ** *p* = 0.0028, and **** *p* < 0.0001. Adapted from Ref. [130] under the terms of the Creative Commons Attribution 4.0 International License.

**Figure 8:**
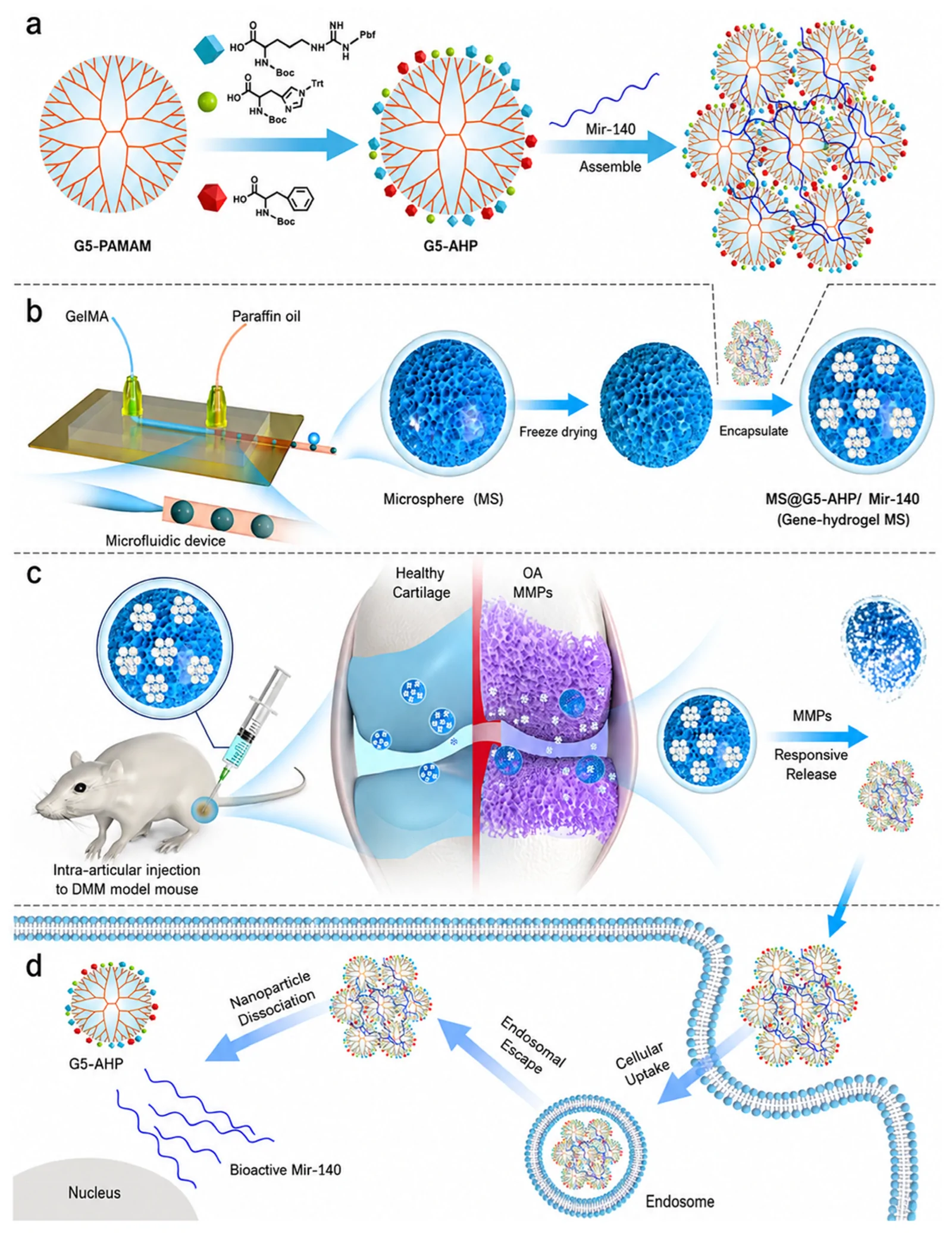
Hierarchical gene–hydrogel microsphere platform for localized miR-140 delivery in osteoarthritis. (**a**) Preparation of modified generation-5 polyamidoamine (G5-AHP) and assembly of G5-AHP/miR-140 nanocomplexes. In (a), cyan diamonds, green circles, and red pentagons represent arginine, histidine, and phenylalanine moieties, respectively, while the blue wavy lines represent miR-140. (**b**) Fabrication of GelMA microspheres using a microfluidic process, followed by incorporation of G5-AHP/miR-140 nanoparticles to generate gene–hydrogel microspheres. (**c**) Intra-articular administration of the gene–hydrogel microspheres and matrix metalloproteinase (MMP)-responsive release of the nanocomplexes within the osteoarthritic joint. (**d**) Cellular uptake and endosomal escape of G5-AHP/miR-140 nanocomplexes, followed by nanoparticle dissociation and intracellular release of bioactive miR-140. Adapted from Ref. [137] under the terms of the Creative Commons Attribution 4.0 International License.

**Table 1:** Representative dynamic network strategies for smart hydrogel-based drug delivery.

Network Type	Dynamic Interaction	Key Characteristics	Relevance to Drug Delivery	Refs
Dynamic covalent	Imine/Schiff base	Rapid formation; pH-sensitive; self-healing	Injectable delivery; acid-responsive release	[28,29,30]
Dynamic covalent	Hydrazone/acylhydrazone	Higher stability; tunable exchange	Sustained and pH-responsive release	[19,24,31,59]
Dynamic covalent	Boronate ester	Rapid reversible exchange; pH/glucose responsive	Dynamic transport; stimuli-responsive release	[23,33]
Dynamic covalent	Disulfide	Thiol–disulfide exchange; reductive cleavage	Redox-triggered/on-demand release	[35,36]
Dynamic covalent	Diels–Alder	Slow reversibility; high network persistence	Long-term and degradation-controlled release	[37,38,39]
Supramolecular	Hydrogen bonding/hydrophobic association	Reversible cooperative interactions	Self-healing; hydrophobic drug retention	[42,43,65]
Supramolecular	Host–guest interaction	Molecular recognition; rapid association/dissociation	Shear-thinning; affinity-controlled release	[17,44,45]
Supramolecular	Ionic/electrostatic interaction	Charge-dependent reversible association	Cargo retention; charge-dependent diffusion	[49,50]
Metal–ligand	Metal coordination	Tunable bond lifetime; self-healing	Adaptive mechanics; sustained/triggered release	[51,52,53]
Hybrid dynamic	Multiple dynamic networks/IPNs	Multiple interaction strengths and timescales	Stability–dynamicity balance; programmable release	[58,59,60]

**Table 2:** Representative quantitative and practical comparison of dynamic hydrogel interaction classes.

Interaction Class	Representative System	Representative Quantitative Feature	Practical Implication	Main Consideration	Ref.
Dynamic covalent	Ketone–hydrazide	Gelation: seconds–hours; degradation: hours–months; mechanics tunable over nearly one order of magnitude	Tunable in situ gelation and long-term degradation	Reaction rate and stability depend strongly on carbonyl chemistry and environment	[31]
Supramolecular	CB[7]–adamantane host–guest	Gelation tunable from seconds to hours; association constant up to ~10^14^ M^−1^	Rapid injectability with tunable assembly and strong molecular recognition	Competing guests and interaction strength can alter gelation and cargo retention	[46]
Metal–ligand	Histidine–metal coordination	Relaxation spans several orders of magnitude; representative modes range from ~0.01 to 100 s depending on metal identity/composition	Wide control of stress relaxation and self-healing without changing polymer architecture	pH, metal concentration, and ion biocompatibility require careful control	[53]
Hybrid dynamic	Boronate ester + acylhydrazone	Immediate gelation; G′ ≈ 0.5 kPa at 300 s and ~2.3 kPa at the first plateau; healing recovery up to ~85.6%	Fast network formation can be combined with slower mechanical reinforcement	Increased network complexity may complicate formulation and reproducibility	[60]

**Table 4:** Common mathematical models used to interpret drug release from hydrogel systems.

Model	Representative Equation	Main Interpretation	Relevance to Dynamic Hydrogels	Main Limitation
Zero-order	M_t_ = M_0_ + k_0_ t	Constant release rate	Useful for near-constant or programmed release	Rarely maintained over the entire release period
First-order	M_t_ /M∞ = 1 − e^−k1 t^	Release rate decreases with remaining releasable drug	Applicable to concentration-dependent release	Does not explicitly account for network remodeling
Higuchi	M_t_ = k_h_ t^1/2^	Diffusion-controlled release	Useful when diffusion through the hydrated matrix dominates	Assumes an approximately static diffusion environment
Korsmeyer–Peppas	M_t_/M∞ = k_kp_t^n^	Empirical distinction between diffusion and anomalous transport	Useful when diffusion and polymer relaxation are coupled	Primarily applicable to the initial portion of release and geometry affects
Weibull	M_t_/M∞ = 1 − exp[−(t/α)^β^]	Flexible empirical description of release-curve shape	Useful for complex, multiphasic, or stimuli-responsive profiles	Good fitting does not identify a unique physical mechanism

**Table 5:** Cargo-specific design considerations for dynamic hydrogel drug delivery.

Cargo Class	Key Cargo Properties/Challenges	Major Hydrogel Design Requirements	Representative Strategies
Small molecules	Small size; rapid diffusion and burst release; variable hydrophobicity	Improve retention without excessive network densification; control diffusion and depot lifetime	Reversible affinity, hydrophobic domains, dynamic covalent conjugation, controlled degradation
Proteins/peptides	Larger size; conformational sensitivity; enzymatic degradation; activity must be preserved	Mild loading conditions; maintain bioactivity; balance retention with complete release	Affinity interactions, mesh-size control, degradable secondary reservoirs
Nucleic acids	Large, negatively charged, nuclease-sensitive; poor cellular uptake and endosomal escape	Protect cargo, prolong local residence, and enable efficient intracellular delivery	Polymeric or lipid nanocarriers embedded within injectable hydrogel depots
Nanoparticle-based therapeutics	Particle size and surface chemistry govern mobility; aggregation or excessive network binding may limit release	Match mesh accessibility and particle–network affinity while maintaining eventual carrier release	Hierarchical nanoparticle–hydrogel systems with controlled network relaxation or degradation

**Table 6:** Key characterization methods for dynamic hydrogels and their relevance to drug delivery.

Material Property	Representative Characterization Methods	Key Information Obtained	Relevance to Drug Delivery
Rheology/viscoelasticity	Oscillatory rheology (G′ and G″), stress-relaxation tests, cyclic strain measurements	Stiffness, relaxation behavior, network recovery	Injectability, depot stability, adaptation after administration
Swelling/mesh accessibility	Equilibrium swelling ratio, water-content measurement, mesh-size estimation	Water uptake and accessibility of transport pathways	Cargo diffusion, retention, and burst-release tendency
Self-healing/recovery	Step-strain or cyclic rheology, cut-and-heal tests	Recovery after network disruption	Structural recovery after injection and maintenance of depot integrity
Degradation/erosion	Time-dependent mass-loss measurements, changes in mechanical or rheological properties during incubation	Rate of network breakdown and material persistence	Depot lifetime and degradation-controlled drug release
Molecular transport/release	FRAP or diffusion measurements, cumulative drug-release studies	Cargo mobility, effective diffusivity, and release kinetics	Prediction of therapeutic retention and release duration

## Data Availability

No new data were created or analyzed in this study. Data sharing is not applicable to this article.

## Abbreviations

The following abbreviations are used in this manuscript:

PEG	Poly(ethylene glycol)
ROS	Reactive oxygen species
NIR	Near-infrared
LNP	Lipid nanoparticle
